# Monotreatment With Conventional Antirheumatic Drugs or Glucocorticoids in Rheumatoid Arthritis: A Network Meta-Analysis

**DOI:** 10.1001/jamanetworkopen.2023.35950

**Published:** 2023-10-06

**Authors:** Louise S. Guski, Gesche Jürgens, Hugo Pedder, Niels K. G. Levinsen, Stig E. Andersen, Nicky J. Welton, Niels Graudal

**Affiliations:** 1 Clinical Pharmacology Unit, Zealand University Hospital, Roskilde, Denmark; 2 Department of Population Health Science, Bristol Medical School, University of Bristol, Bristol, United Kingdom; 3 Center for Rheumatology and Spine Diseases, The Lupus and Vasculitis Clinic, Copenhagen University Hospital Rigshospitalet, Copenhagen, Denmark

## Abstract

**Importance:**

This is the first network meta-analysis to assess outcomes associated with multiple conventional synthetic disease-modifying antirheumatic drugs and glucocorticoid.

**Objective:**

To analyze clinical outcomes after treatment with conventional synthetic disease-modifying antirheumatic drugs and glucocorticoid among patients with rheumatoid arthritis.

**Data Sources:**

With no time restraint, English language articles were searched in MEDLINE, Embase, Cochrane Central, ClinicalTrials.gov, and reference lists of relevant meta-analyses until September 15, 2022.

**Study Selection:**

Four reviewers in pairs of 2 independently included controlled studies randomizing patients with rheumatoid arthritis to mono–conventional synthetic disease-modifying antirheumatic drugs, glucocorticoid, placebo, or nonactive treatment that recorded at least 1 outcome of tender joint count, swollen joint count, erythrocyte sedimentation rate, and C-reactive protein level. Of 1098 assessed articles, 130 articles (132 interventions) were included.

**Data Extraction and Synthesis:**

The review followed the Preferred Reporting Items for Systematic Reviews and Meta-analyses reporting guideline, and data quality was assessed by the Cochrane risk of bias tool RoB 2. Data were extracted by a single author and checked independently by 2 authors. Data were analyzed using a random effect model, and data analysis was conducted from June 2021 to February 2023.

**Main Outcomes and Measures:**

A protocol with hypothesis and study plan was registered before data recording. The most complete of recorded outcomes (tender joint count) was used as primary outcome, with imputations based on other outcomes to obtain a full analysis of all studies. Absolute change adjusted for baseline disease activity was assessed.

**Results:**

A total of 29 interventions in 275 treatment groups among 132 randomized clinical trials (mean [range], 71.0% [27.0% to 100%] females in studies; mean [range] of ages in studies, 53 [36 to 70] years) were identified, which included 13 260 patients with rheumatoid arthritis. The mean (range) duration of RA was 79 (2 to 243) months, and the mean (range) disease activity score was 6.3 (4.0 to 8.8). Compared with placebo, oral methotrexate was associated with a reduced tender joint count by 5.18 joints (95% credible interval [CrI], 4.07 to 6.28 joints). Compared with methotrexate, glucocorticoid (−2.54 joints; 95% CrI, −5.16 to 0.08 joints) and remaining drugs except cyclophosphamide (6.08 joints; 95% CrI, 0.44 to 11.66 joints) were associated with similar or lower tender joint counts.

**Conclusions and Relevance:**

This study’s results support the present role of methotrexate as the primary reference conventional synthetic disease-modifying antirheumatic drug.

## Key Points

**Question:**

Which conventional treatments are associated with the best clinical outcomes among patients with rheumatoid arthritis?

**Findings:**

In this meta-analysis of 29 interventions investigated in 132 randomized clinical trials of 13 260 patients with rheumatoid arthritis, methotrexate was the most thoroughly investigated drug with the most reliable outcome.

**Meaning:**

These findings suggest that methotrexate is the anchor untargeted, conventional treatment for rheumatoid arthritis but that in case of suboptimal outcomes or unacceptable adverse events, several alternative conventional treatments are available.

## Teaser

The meta-analysis investigates the association of conventional antirheumatic drugs and glucocorticoids with clinical outcomes among patients with rheumatoid arthritis.

## Introduction

Since the discovery of treatments for rheumatoid arthritis (RA) using gold in 1932 and glucocorticoid (GC) in 1949,^1,2^ many synthetic disease-modifying antirheumatic drugs (csDMARDs) have been introduced.^3^ The use of targeted DMARDs (tDMARDs), including biologic and targeted synthetic,^4,5^ has increased since 2000.

Previously, outcome measures of RA reflected joint inflammation (morning stiffness, number of tender joints [tender joint count (TJC)]) and swollen joints [swollen joint count (SJC)], and limitation of joint motion), systemic inflammation (erythrocyte sedimentation rate [ESR] and C-reactive protein [CRP] level^6,7^), and joint destruction (radiographic evaluation^8,9^). While the latter still is a separate outcome, clinical and biochemical variables have been integrated in composite disease activity scores and improvement scores,^10,11,12,13^ which may include TJC, SJC, ESR, and CRP.

A 2020 review^14^ of network meta-analyses (NMAs) of DMARDs included 37 NMAs of tDMARDs and no csDMARDs. However, 70% of patients treated for RA receive csDMARDs and 25% receive tDMARDs,^15^ and a combination of 2 to 3 csDMARDs may be associated with similar effectiveness as tDMARDs.^16,17^ Furthermore, tDMARDs are used only by specialists, whereas general practitioners may use csDMARDs. Thus, outcomes associated with csDMARDs may be of general interest. Previously, we estimated the relative effect sizes associated with 10 csDMARDs and GC using the sporadically reported radiographic destruction outcome.^16^ The objective in this study was to estimate relative effect sizes associated with multiple csDMARDS and GC in an integrated NMA of RCTs using frequently reported outcomes. Given that older RCTs of csDMARDs rarely used composite outcomes, we analyzed differences in absolute change of measures of TJC, SJC, ESR, and CRP adjusted for potential effect modifiers.

## Methods

### Protocol and Registration

A protocol for this meta-analysis was registered at Prospero on July 5, 2020 (CRD42020184585). The study was approved by the Faculty of Health and Medical Sciences, University of Copenhagen, and is reported according to the Preferred Reporting Items for Systematic Reviews and Meta-analyses (PRISMA) reporting guideline.

### Eligibility Criteria

We included trials that randomly allocated patients with RA to csDMARD, GC, placebo, or a pharmacologic non–disease-modifying comparator (no treatment; pain-relieving treatment, including nonsteroidal anti-inflammatory drugs; or very-low dose of the investigated DMARD) and that reported at least 1 of the outcome variables (eMethods 1 in Supplement 1).

Information sources* (*eMethods 2 in Supplement 1) included MEDLINE, Cochrane Central, Embase, Clinicaltrials.gov, and reference lists of included studies and previous meta-analyses. The search was conducted on June 13, 2018, and updated on January 12, 2022. On September 15, 2022, we performed a post hoc screening search in PubMed covering September 15, 2021, to September 15, 2022.

### Search, Study Selection, and Data Collection

The electronic search string is shown in eMethods 2 in Supplement 1. Irrelevant studies were eliminated by 1 author (L.S.G.). Exclusion criteria could be not an RCT, study population including patients without RA, no report of defined outcomes, or no use of csDMARDs. Then, 2 pairs of authors (L.S.G./G.J. and S.E.A./N.G.), who were blinded to each other’s decisions, independently repeated the procedure while categorizing studies from the remaining pool according to type of intervention. Data were recorded on a standardized extraction form using Excel spreadsheet software version 16.0.166626.20134 (Microsoft). Data were collected by a single author (L.S.G.). All data were independently controlled by 2 authors (N.K.G.L. and N.G.). Any disagreements were resolved by discussion between 2 authors (L.S.G. and N.G.). See eMethods 3 in Supplement 1.

### Data Items

The study population was defined as the number of patients who were randomized and received at least 1 dose of drug. We estimated factors to convert 1 outcome to another outcome to be able to impute missing outcomes. Thus, the estimated relative effect size of the missing outcome would correspond to the relative effect size of the reported outcome. Missing TJC values for the primary outcome and for the disease activity score based on 28 joints (DAS28) estimation were imputed as explained in eMethods 4 and 5 in Supplement 1. Missing SJC and ESR values for the DAS28 estimation were imputed as explained in eMethods 5 in Supplement 1. If TJC and SJC were assessed on more than 28 joints, TJC and SJC were converted to an assessment of 28 joints (TJC28 and SJC28) based on conversion factors estimated by Fuchs and Pincus^18^ shown in eMethods 5 in Supplement 1. Then DAS28 was calculated by the following formula: DAS28 = (0.56 × sqrt[tjc28] + 0.28 × sqrt(sjc28) + 0.70 × Ln[ESR]) × 1.08 + 0.16. To calculate SE, we used a recently calculated mean SD of 1.1.^19^ See eMethods 4 and 5 in Supplement 1.

### Geometry of the Network

We drew the network using RStudio statistical software version 1.4.1103 (RStudio)^20^ by a code from the develop branch. Chloroquine and hydroxychloroquine were merged into 1 node and combined across doses. Placebo and comparators without systemic anti-inflammatory properties were merged into the placebo node. All other nodes were defined by a single intervention and combined across doses.

### Statistical Analysis

#### Risk of Bias Within Individual Studies

Risk of bias was assessed at study level by the Cochrane risk of bias tool RoB 2.^21^ See eMethods 6 in Supplement 1.

#### Summary Measures

For our primary analysis of all 132 studies (represented by 130 publications^22,23,24,25,26,27,28,29,30,31,32,33,34,35,36,37,38,39,40,41,42,43,44,45,46,47,48,49,50,51,52,53,54,55,56,57,58,59,60,61,62,63,64,65,66,67,68,69,70,71,72,73,74,75,76,77,78,79,80,81,82,83,84,85,86,87,88,89,90,91,92,93,94,95,96,97,98,99,100,101,102,103,104,105,106,107,108,109,110,111,112,113,114,115,116,117,118,119,120,121,122,123,124,125,126,127,128,129,130,131,132,133,134,135,136,137,138,139,140,141,142,143,144,145,146,147,148,149,150,151^) we used the most frequently reported outcome, TJC. This included imputed TJC values for studies that did not measure TJC (eMethods 5 in Supplement 1), denominated TJCi.

For our sensitivity analysis, we used ESR, CRP, SJC, TJC without imputations, estimated TJC28 with imputations (TJC28i) (eMethods 5 in Supplement 1), and estimated DAS28 with imputations (DAS28i) (eMethods 5 in Supplement 1) as efficacy measures. The outcome measure for TJCi, TJC, SJC, ESR, CRP, DAS28i, and TJC28i was defined as the difference between the baseline mean value and the posttreatment mean value. We used absolute changes, which statistically are more efficient than percentage changes.^152^ Treatment outcomes of TJCi were categorized as favorable compared with placebo and methotrexate, favorable compared with placebo and not different from methotrexate, favorable compared with placebo but unfavorable compared with methotrexate, not different from placebo or methotrexate, and not different from placebo but unfavorable compared with methotrexate. Favorable means that the 2.5% credible interval (CrI) was higher than the 97.5% CrI of the compared drug. Unfavorable means that the 97.5% CrI was lower than the 2.5% CrI of the compared drug. Not different means that the CrIs were overlapping. Rankograms were plotted for all treatments. Finally, we assessed dropout rates. For each study the number of dropouts was recorded from the pool of all randomized patients. The sum of dropouts for each treatment group was divided by the sum of randomized patients for each treatment group.

#### Planned Methods of Analyses^153,154,155,156,157,158^

We conducted our network meta-analysis following the method of Lu and Ades^153^ using WinBUGS code taken from NICE Technical Support Document 2,^154^ in which handling of multigroup trials is a built-in function. We ran models using prior 0 to 5 as explained in eMethods 7 in Supplement 1. Post hoc, we reran all models with different priors and created density plots, and we ran all models with a uniform 0 to 20 prior in August 2023.

#### Additional Analyses

Network metaregression models were fitted using WinBUGS^155^ with code from NICE Technical Support Document 3,^158^ assuming a common regression effect size across pooled treatment effect sizes vs methotrexate. For effect modifier regression analyses, we ran univariate regression analyses for possible numerical effect modifiers: duration of study, duration of rheumatoid arthritis, publication year, mean age, percentage female, percentage with rheumatoid-factor positivity, baseline DAS28, and bias score based on the Cochrane risk of bias tool. See eMethods 8 in Supplement 1.

Subgroup analyses (eMethods 8 in Supplement 1) were used to test for the association of categorical variables (type of statistical analysis [intention to treat or completer], concomitant GC during study allowed [yes or no], and previous use of DMARDs (no, yes unspecified, or yes with inadequate response]) with outcomes. To investigate the suitability of merging chloroquine and hydroxychloroquine into 1 node, we ran the main model of TJCi separating the 2 treatments. Data were analyzed from June 2021 to February 2023 and post hoc in August 2023.

## Results

### Study Characteristics

A total of 29 interventions among 132 randomized clinical trials (RCTs) represented by 130 publications^22,23,24,25,26,27,28,29,30,31,32,33,34,35,36,37,38,39,40,41,42,43,44,45,46,47,48,49,50,51,52,53,54,55,56,57,58,59,60,61,62,63,64,65,66,67,68,69,70,71,72,73,74,75,76,77,78,79,80,81,82,83,84,85,86,87,88,89,90,91,92,93,94,95,96,97,98,99,100,101,102,103,104,105,106,107,108,109,110,111,112,113,114,115,116,117,118,119,120,121,122,123,124,125,126,127,128,129,130,131,132,133,134,135,136,137,138,139,140,141,142,143,144,145,146,147,148,149,150,151^ (mean [range], 71.0% [27.0%-100%] females in studies; mean [range] of ages in studies, 53 [36-70] years) were identified, which included 13 260 patients with rheumatoid arthritis. The number of studies with measured outcomes was 118 studies^23,26,28,29,30,34,36,37,38,39,40,41,42,43,44,45,46,47,48,49,50,51,52,53,54,55,56,57,58,59,60,61,62,63,64,66,67,68,69,70,71,72,73,74,75,76,77,79,80,81,82,83,84,85,87,88,89,92,93,94,95,96,97,98,99,100,101,102,103,104,105,106,108,109,110,111,112,113,114,115,116,117,118,119,120,121,122,123,124,125,126,127,128,129,130,131,132,133,134,135,136,137,138,139,140,141,142,143,144,145,146,147,148,149,150,151^ for TJC, 116 studies^22,23,24,25,26,27,28,29,30,32,34,35,36,37,38,39,40,41,42,43,44,45,46,47,48,49,50,51,52,53,54,55,56,57,58,59,60,61,62,63,64,65,66,67,68,69,70,71,72,73,74,75,76,77,78,80,81,82,84,85,87,88,89,90,91,92,93,94,96,97,98,99,100,101,104,105,106,107,108,110,111,112,113,114,115,116,117,118,119,120,121,122,123,124,125,127,128,129,135,136,137,140,141,142,143,144,146,147,148,149,150,151^ for ESR, 97 studies^22,26,29,30,31,32,33,37,39,40,41,44,45,46,50,51,52,54,57,58,60,62,63,65,67,68,69,70,71,72,73,74,75,76,77,78,80,81,82,83,84,86,87,88,92,93,94,96,97,98,100,101,102,103,105,107,108,109,110,111,113,114,115,116,118,119,120,122,123,124,125,127,128,129,130,131,132,133,134,135,137,138,139,140,141,142,143,144,145,146,147,148,149,150,151^ for SJC, and 47 studies^40,45,56,58,64,66,67,79,85,87,95,97,98,100,104,110,111,113,114,115,116,117,122,124,125,126,127,128,129,131,132,133,134,136,137,138,140,141,142,144,146,147,148,149,150,151^ for CRP. The number of study groups and participants and the dropout rate (percentage) for each intervention group appears in the Table. The dropout rate ranged from 36 of 291 participants (12.4%) for iguratimod to 91 of 169 participants (53.5%) for pyritinol. Characteristics and results of individual studies and interventions are presented in eTable 1 in Supplement 1. At baseline, the mean (range) for duration of RA was 79 (2-243) months, TJC was 18.6 (5.3-48.2) joints, SJC was 15.8 (5.0-41.6) joints, baseline ESR was 51.6 (23.8-91.6) mm/h, baseline CRP level was 4.12 (1.10-8.64) mg/dL (to convert to milligrams per liter, multiply by 10), DAS28 was 6.3 (4.0-8.8), and rheumatoid factor positivity was 77% (0%-100%).

**Table.** Primary Outcome and Sensitivity Outcomes With Methotrexate as Reference

Drug	Intervention groups, No.	Participants		Reduction in outcome (95% CrI)^a^						
		Randomized and treated, No.	Dropped out, No./No. randomized (%)	TJCi (n = 132)	TJC (n = 118)	SJC (n = 97)	ESR, mm/h (n = 116)	CRP level, mg/L (n = 47)	DAS28i (n = 132)	TJC28i (n = 132)
Methotrexate	31	2344	539/2549 (21.1)	0 [Reference]	0 [Reference]	0 [Reference]	0 [Reference]	0 [Reference]	0 [Reference]	0 [Reference]
Sulfasalazine	18	762	399/964 (41.4)	−1.90 (−3.15 to −0.57)^b^	−1.91 (−3.20 to −0.54)^b^	−1.39 (−2.50 to −0.25)^b^	1.43 (−2.62 to 5.55)^c^	−4.91 (−14.24 to 4.97)^c^	−0.14 (−0.90 to 0.62)^c^	−1.69 (−2.64 to −0.69)^b^
Leflunomide	8	1321	361/1397 (25.8)	−0.26 (−1.23 to 0.89)^c^	−0.25 (−1.22 to 0.91)^c^	−0.92 (−1.57 to −0.18)^b^	−7.53 (−10.50 to −4.08)^b^	0.90 (−5.10 to 7.57)^d^	−0.26 (−0.76 to 0.24)^c^	−0.35 (−1.04 to 0.51)^c^
Cyclosporine	11	492	147/613 (24.0)	−0.79 (−2.41 to 0.88)^c^	−0.78 (−2.43 to 0.91)^c^	−1.14 (−2.39 to 0.12)^c^	−9.97 (−15.64 to −4.25)^d^	6.52 (−7.98 to 20.64)	−0.32 (−1.28 to 0.66)^d^	−0.86 (−2.04 to 0.34)^c^
Injected gold	25	777	439/1126 (39.0)	−0.71 (−2.10 to 0.61)^c^	−0.72 (−2.13 to 0.62)^c^	−0.80 (−2.07 to 0.45)^c^	0.29 (−4.07 to 4.65)^c^	5.09 (−6.25 to 16.72)	−0.05 (−0.82 to 0.74)^d^	−1.22 (−2.26 to −0.17)^b^
Chloroquine	19	662	133/721 (18.4)	−2.51 (−4.02 to −0.93)^b^	−2.41 (−4.03 to −0.73)^b^	−2.20 (−3.62 to −0.81)^b^	−7.25 (−11.86 to −2.56)^b^	−11.19 (−23.13 to 0.36)^d^	−0.42 (−1.32 to 0.49)^d^	−2.15 (−3.27 to −0.97)^b^
D-penicillamine	28	743	321/974 (33.0)	−2.21 (−3.71 to −0.67)^b^	−2.28 (−3.82 to −0.68)^b^	−0.90 (−2.32 to 0.54)^c^	2.38 (−2.70 to 7.48)^c^	−0.65 (−16.34 to 14.81)^d^	−0.10 (−1.03 to 0.80)^d^	−1.46 (−2.63 to −0.27)^b^
Azathioprine	12	347	153/449 (34.1)	−2.57 (−4.21 to −0.88)^b^	−2.61 (−4.31 to −0.87)^b^	−1.40 (−2.88 to 0.08)^c^	−7.00 (−13.88 to 0.02)^d^	−1.57 (−16.67 to 13.43)^d^	−0.34 (−1.47 to 0.82)^d^	−1.55 (−2.93 to −0.18)^b^
Oral gold	20	1200	462/1417 (32.6)	−2.70 (−4.19 to −1.18)^b^	−3.03 (−4.54 to −1.46)^b^	−2.45 (−3.76 to −1.11)^b^	−6.70 (−10.88 to −2.46)^b^	−16.22 (−30.16 to −2.42)^d^	−0.45 (−1.20 to 0.30)^d^	−2.35 (−3.37 to −1.29)^b^
Cyclophosphamide	3	41	10/51 (19.6)	6.08 0.44 to 11.66)^e^	8.34 (2.06 to 14.61)^e^	8.42 (4.59 to 12.27)^e^	−0.52 (−11.73 to 10.84)^c^	NA	0.12 (−2.14 to 2.32)^d^	3.53 (−0.17 to 7.26)^c^
Levamisole	10	306	146/408 (35.8)	−2.46 (−4.11 to −0.71)^b^	−2.43 (−4.10 to −0.67)^b^	−2.73 (−4.07 to −1.37)^c^	1.37 (−5.31 to 8.20)^c^	10.18 (−6.76 to 26.83)	−0.15 (−1.35 to 1.03)^d^	−1.05 (−2.48 to 0.37)^c^
Tacrolimus	3	275	115/287 (40.1)	1.31 (−1.46 to 4.12)^c^	1.39 (−1.41 to 4.22)^c^	−0.82 (−2.81 to 1.16)^c^	1.73 (−6.26 to 9.74)^c^	0.48 (−12.18 to 13.71)	−0.10 (−1.45 to 1.25)^d^	0.48 (−1.45 to 2.43)^c^
*Tripterygium wilfordii*	2	113	33/129 (25.6)	−1.29 (−5.69 to 3.11)^d^	−1.30 (−5.67 to 3.06)^d^	−0.32 (−2.77 to 2.12)^c^	−1.65 (−11.02 to 7.85)^c^	−0.42 (−14.49 to 14.19)^d^	−0.028 (−1.93 to 1.90)^d^	−1.19 (−4.43 to 2.05)^d^
Iguratimod	3	290	36/291 (12.4)	−1.13 (−2.99 to 0.71)^c^	−1.11 (−2.98 to 0.73)^c^	−0.75 (−2.05 to 0.56)^c^	−0.61 (−7.85 to 6.77)^c^	−0.01 (−9.58 to 9.49)	−0.12 (−1.26 to 1.01)^d^	−0.94 (−2.49 to 0.62)^c^
Timegadine	1	19	12/23 (52.2)	−4.66 (−18.77 to 9.35)^d^	−4.73 (−18.77 to 9.31)^d^	−4.20 (−16.03 to 7.95)^d^	20.17 (−4.11 to 44.84)^c^	NA	0.05 (−4.86 to 4.86)^d^	−2.95 (−11.78 to 5.84)^d^
Bucillamine	1	24	3/24 (12.5)	−0.85 (−12.94 to 11.03)^d^	−0.95 (−13.10 to 11.14)^d^	1.29 (−6.54 to 9.20)^d^	1.95 (−28.56 to 32.39)^d^	10.34 (−27.66 to 48.19)^d^	0.11 (−4.49 to 4.81)^d^	−0.50 (−9.01 to 7.99)^d^
Tiopronin	1	25	9/28 (32.1)	−3.00 (−9.63 to 3.70)^d^	−3.06 (−9.71 to 3.63)^d^	NA	9.27 (−11.76 to 30.36)^c^	NA	0.019 (−3.79 to 3.76)^d^	−2.07 (−6.95 to 2.90)^d^
Pyritinol	1	139	91/169 (53.5)	−2.11 (−6.22 to 2.03)^d^	−2.43 (−6.51 to 1.74)^d^	NA	1.23 (−9.57 to 12.03)^c^	NA	−0.23 (−2.11 to 1.62)^d^	−1.91 (−4.80 to 1.02)^d^
Mycophenolate	2	286	136/291 (46.7)	−3.57 (−7.09 to −0.03)^f^	−3.50 (−7.02 to 0.04)^d^	−3.12 (−6.11 to −0.12)^f^	−12.26 (−20.16 to −4.37)^f^	−13.23 (−27.55 to 1.34)^d^	−0.63 (−2.31 to 1.03)^d^	−2.51 (−5.78 to 0.78)^d^
OM-8980	2	86	23/96 (24.0)	−2.20 (−5.74 to 1.34)^d^	−2.44 (−6.02 to 1.11)^d^	−2.89 (−4.55 to −1.16)^f^	−6.38 (−17.94 to 5.13)^d^	NA	−0.32 (−2.37 to 1.72)^d^	−1.77 (−4.33 to 0.78)^d^
Lobenzarit	1	86	36/115 (31.3)	−3.55 (−7.10 to 0.0074)^d^	NA	−2.82 (−5.89 to 0.29)^d^	−5.42 (−20.82 to 9.911)^d^	NA	−0.55 (−2.63 to 1.51)^d^	−3.11 (−5.34 to −0.88)^f^
Rheumacon	2	80	13/80 (16.3)	−2.82 (−5.24 to −0.32)^f^	−2.96 (−5.39 to −0.46)^f^	−2.84 (−7.96 to 2.20)^d^	−21.84 (−35.67 to −7.72)^d^	−16.39 (−35.70 to 3.29)^d^	−0.70 (−2.73 to 1.28)^d^	−2.36 (−4.05 to −0.63)^f^
Prospidin	1	23	4/27 (14.8)	0.21 (−7.19 to 7.65)^d^	0.17 (−7.28 to 7.68)^d^	3.33 (−4.47 to 11.12)^d^	9.36 (−3.58 to 22.25)^c^	7.28 (−8.41 to 23.23)	0.51 (−3.15 to 4.2)^d^	0.052 (−4.57 to 4.74)^d^
Aminopterin	1	11	2/13 (15.4)	10.37 (−12.97 to 33.61)^d^	5.50 (−17.38 to 28.28)^d^	8.34 (−13.84 to 30.55)^d^	NA	NA	0.92 (−6.44 to 8.37)^d^	8.36 (−8.07 to 24.75)^d^
Eazmov plus	1	18	6/21 (28.6)	−2.84 (−15.14 to 9.72)^d^	−2.43 (−15.08 to 10.12)^d^	−2.64 (−10.33 to 5.01)^d^	−11.03 (−23.63 to 1.52)^d^	NA	−0.35 (−4.80 to 3.99)^d^	−2.26 (−14.32 to 10.24)^d^
Dapsone	2	44	13/57 (22.8)	−1.38 (−5.24 to 2.49)^c^	−0.90 (−5.11 to 3.28)^c^	0.46 (−5.31 to 6.19)^d^	−3.66 (−19.36 to 12.19)^d^	4.27 (−18.95 to 27.47)^d^	−0.17 (−2.88 to 2.49)^d^	−1.38 (−4.05 to 1.35)^c^
Glucocorticoid	4	153	28/122 (23.0)	−2.54 (−5.16 to 0.08)^c^	−3.09 (−6.56 to 0.30)^d^	−3.25 (−5.28 to −1.24)^f^	−1.76 (−10.78 to 7.36)^d^	−12.16 (−32.55 to 7.82)^d^	−0.30 (−1.92 to 1.29)^d^	−1.942 (−4.00 to 0.15)^c^
Injectable methotrexate	2	206	32/214 (15.0)	1.04 (−2.24 to 4.25)^c^	0.98 (−2.67 to 4.51)^c^	−0.80 (−2.97 to 1.36)^c^	−4.77 (−17.23 to 7.60)^d^	NA	0.61 (−1.80 to 2.96)^d^	0.59 (−1.41 to 2.55)^c^
Placebo	60	2900	1042/3253 (32.0)	−5.18 (−6.27 to −4.09)	−4.83 (−6.08 to −3.61)	−4.44 (−5.36 to −3.53)	−13.35 (−16.79 to −9.87)	−13.3 (−21.81 to −4.97)	−0.88 (−1.51 to −0.25)	−4.14 (−4.97 to −3.28)

Abbreviations: CRP, C-reactive protein; DAS28i, disease activity score based on 28 joints with imputations; ESR, erythrocyte sedimentation rate; NA, not applicable (no comparisons of this outcome within this intervention); SJC, swollen joint count; TJC, tender joint count; TJC28i, tender joint score based on 28 joints with imputations;TJCi, tender joint count imputed.

SI conversion factor: To convert CRP to milligrams per deciliter, divide by 10.

^a^ A negative sign indicates that the outcome reduction is less than the reduction associated with methotrexate.

^b^ Favorable vs placebo and unfavorable vs methotrexate.

^c^ Favorable vs placebo and not different vs methotrexate.

^d^ Not different vs placebo and not different vs methotrexate.

^e^ Favorable vs placebo and favorable vs methotrexate.

^f^ Not different vs placebo and unfavorable vs methotrexate.

### Study Selection

**Figure 1.**
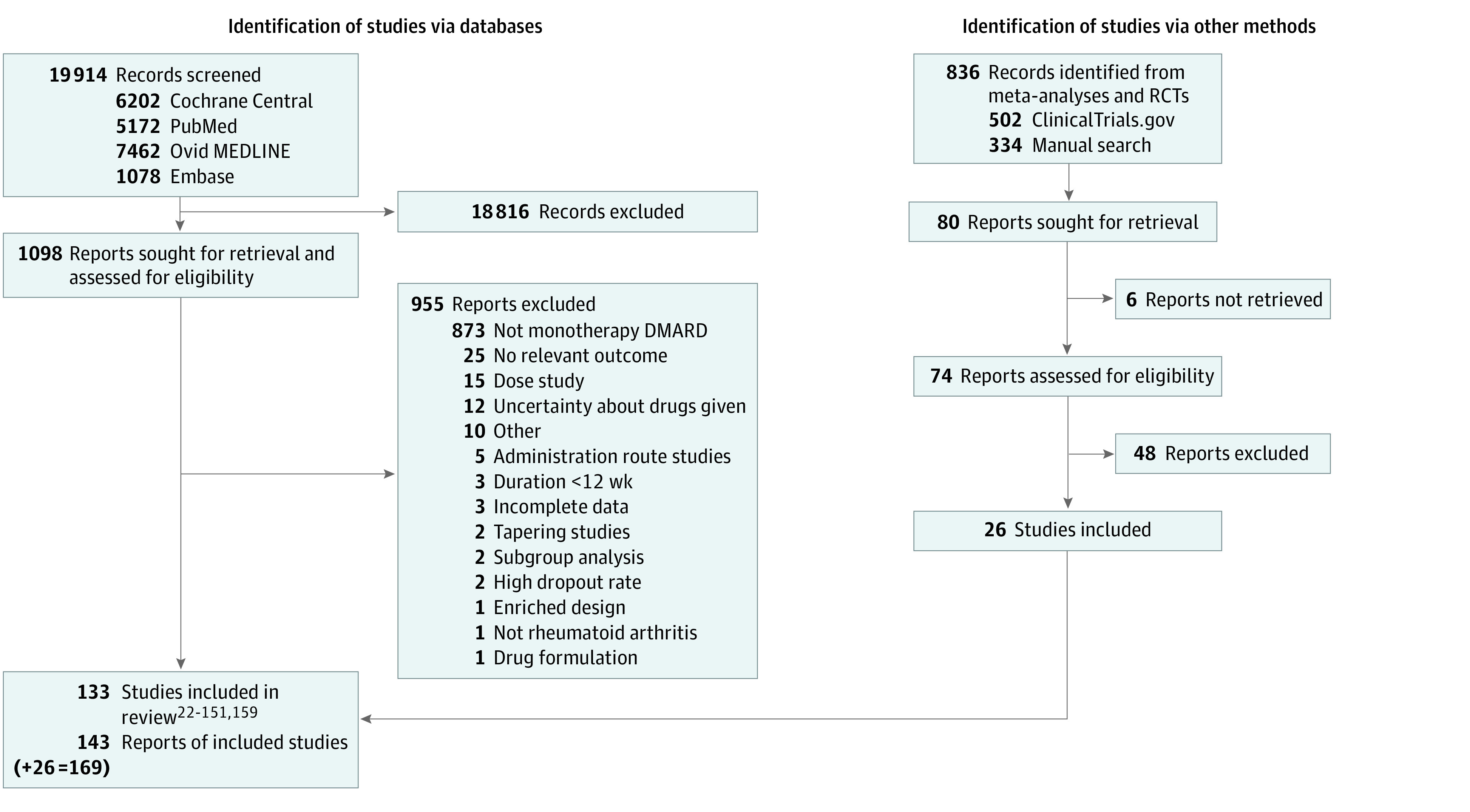
Flowchart of Study Identification DMARD indicates disease-modifying antirheumatic drug; RCT, randomized clinical trial.

See eResults 1 in Supplement 1. Figure 1 shows a flowchart of the identification of the pool of RCTs. We included 131 publications,^22,23,24,25,26,27,28,29,30,31,32,33,34,35,36,37,38,39,40,41,42,43,44,45,46,47,48,49,50,51,52,53,54,55,56,57,58,59,60,61,62,63,64,65,66,67,68,69,70,71,72,73,74,75,76,77,78,79,80,81,82,83,84,85,86,87,88,89,90,91,92,93,94,95,96,97,98,99,100,101,102,103,104,105,106,107,108,109,110,111,112,113,114,115,116,117,118,119,120,121,122,123,124,125,126,127,128,129,130,131,132,133,134,135,136,137,138,139,140,141,142,143,144,145,146,147,148,149,150,151,159^ which represented 133 studies. In 1 study,^159^ the 2 interventions (sulfapyridine and 5-aminosalicylic acid) were not connected to the network. The remaining 132 studies^22,23,24,25,26,27,28,29,30,31,32,33,34,35,36,37,38,39,40,41,42,43,44,45,46,47,48,49,50,51,52,53,54,55,56,57,58,59,60,61,62,63,64,65,66,67,68,69,70,71,72,73,74,75,76,77,78,79,80,81,82,83,84,85,86,87,88,89,90,91,92,93,94,95,96,97,98,99,100,101,102,103,104,105,106,107,108,109,110,111,112,113,114,115,116,117,118,119,120,121,122,123,124,125,126,127,128,129,130,131,132,133,134,135,136,137,138,139,140,141,142,143,144,145,146,147,148,149,150,151^ were included in the NMA. In the post hoc screening in PubMed on September 15, 2022, we did not identify relevant studies (eResults 1 in Supplement 1).

### Network Structure and Geometry

**Figure 2.**
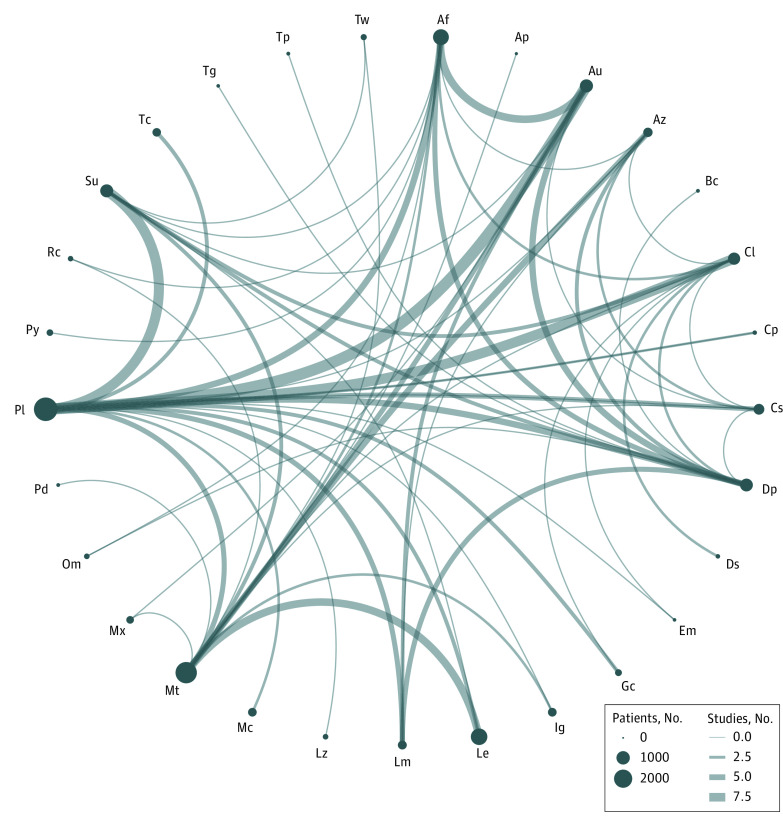
Geometry of the Network The size of the node reflects the number of patients included in the analysis for each drug, and the thickness of the lines between 2 nodes reflects the number of studies comparing the 2 treatments. The interventions included are Mt (methotrexate; 31 study groups, 2344 patients), Su (sulfasalazine; 18 study groups, 762 patients), Le (leflunomide; 8 study groups, 1321 patients), Cs (cyclosporine; 11 study groups, 492 patients), Au (injected gold; 25 study groups, 777 patients), Cl (chloroquine/hydroxychloroquine; 19 study groups, 662 patients), Dp (D-penicillamine; 28 study groups, 743 patients), Az (azathioprine; 12 study groups, 347 patients), Af (oral gold; 20 study groups, 1200 patients), Cp (cyclophosphamide; 3 study groups, 41 patients), Lm (levamisole; 10 study groups, 306 patients), Tc (tacrolimus; 3 study groups, 275 patients), Tw (*Tripterygium wilfordii*; 2 study groups, 113 patients), Ig (T-614, iguratimod; 3 study groups, 290 patients), Tg (timegadine; 1 study group, 19 patients), Bc (bucillamine; 1 study group, 24 patients), Tp (tiopronin; 1 study group, 25 patients), Py (pyritinol; 1 study group, 139 patients), Mc (mycophenolate mofetil; 2 study groups, 286 patients), Om (OM-8980; 2 study groups, 86 patients), Lz (lobenzarit; 1 study group, 86 patients), Rc (reumacon; 2 study groups, 80 patients), Pd (prospidin; 1 study group, 23 patients), Ap (aminopterin; 1 study group, 11 patients), Em (eazmov plus; 1 study group, 18 patients), Ds (dapsone; 2 study groups, 44 patients), Gc (glucocorticoid; 4 study groups, 153 patients), Mx (injected methotrexate; 2 study groups, 206 patients), and Pl (placebo; 60 study groups; 2900 patients).

The network is presented in Figure 2. The network comprises 27 mono-DMARD groups (methotrexate, peroral^70,71,72,78,88,90,93,98,100,101,102,103,113,114,121,123,124,127,129,131,132,133,134,135,136,141,145,147,149,150,151^; sulfasalazine^64,77,80,87,91,99,104,105,107,109,117,121,123,127,128,139,146^; leflunomide^120,128,129,132,133,134,141,149^; cyclosporine^76,84,85,92,96,97,111,112,125,130,135^; gold, injectable^26,32,33,34,39,42,44,46,49,51,52,53,59,61,63,69,80,82,83,88,100,124,125,136^; chloroquine/hydroxychloroquine^23,25,27,28,41,66,79,87,95,107,108,110,111,115,116,118,119,121,143^; D-penicillamine^30,34,35,36,38,43,44,46,49,50,52,55,56,57,58,62,64,67,68,73,75,79,89,91,94,95,96,122^; azathioprine^36,41,48,67,68,78,90,97,98,102,112,114^; gold, peroral^41,51,52,53,56,60,63,69,73,74,75,81,83,86,93,101,104,106,116,126^; cyclophosphamide^29,31,37^; levamisole^40,42,45,47,49,50,54,58,67^; tacrolimus^137,140,142^; *Tripterygium wilfordii*^146,150^; iguratimod^144,147,151^; timegadine^57^; bucillamine^122^; tiopronin^55^; pyritinol^106^; mycophenolate^148^; OM-8980^81,94^; lobenzarit^65^; rheumacon^126,131^; prospidin^113^; aminopterin^103^; eazmov plus^143^; dapsone9^66,110^; and methotrexate, injectable^82,145^), 1 GC group,^22,24,119,138^ and 1 placebo group.^22,23,24,25,26,27,28,29,30,31,32,33,35,37,38,39,40,43,45,47,48,53,54,59,60,61,62,63,65,70,71,72,74,76,77,80,84,85,86,92,99,105,108,109,115,117,118,120,128,129,130,137,138,139,140,142,143,144,148^ We identified 275 treatment groups in 121 two-group studies^22,23,24,25,26,27,28,29,30,31,32,33,34,35,36,37,38,39,40,42,43,44,45,46,47,48,50,51,54,55,56,57,58,59,60,61,62,64,65,66,68,69,70,71,72,73,74,75,76,77,78,79,81,82,83,84,85,86,87,88,89,90,91,92,93,94,95,96,97,98,99,100,101,102,103,104,105,106,107,108,109,110,111,112,113,114,115,116,117,118,119,120,122,123,124,125,126,127,130,131,132,133,134,135,136,137,138,139,140,141,142,144,145,146,147,148,149,150,151^ and 11 three-group studies.^41,49,52,53,63,67,80,121,128,129,143^

### Risk of Bias Within Studies

The assigned risk of bias appears in eTable 1 in Supplement 1. Of 130 RCTs^22,23,24,25,26,27,28,29,30,31,32,33,34,35,36,37,38,39,40,41,42,43,44,45,46,47,48,49,50,51,52,53,54,55,56,57,58,59,60,61,62,63,64,65,66,67,68,69,70,71,72,73,74,75,76,77,78,79,80,81,82,83,84,85,86,87,88,89,90,91,92,93,94,95,96,97,98,99,100,101,102,103,104,105,106,107,108,109,110,111,112,113,114,115,116,117,118,119,120,121,122,123,124,125,126,127,128,129,130,131,132,133,134,135,136,137,138,139,140,141,142,143,144,145,146,147,148,149,150,151^ in the network, 122 trials^22,23,24,25,26,27,28,29,30,31,32,33,35,36,38,39,40,41,42,43,44,45,46,47,48,49,50,51,52,53,54,56,57,58,59,60,62,63,64,65,66,67,68,69,70,71,72,73,74,75,76,77,78,79,80,81,82,83,84,85,87,88,89,90,91,92,93,94,95,96,97,98,99,100,101,102,103,104,105,106,107,108,109,110,111,112,113,114,115,116,117,119,120,121,122,123,124,125,126,127,128,129,130,131,132,134,135,136,137,139,140,141,142,143,144,145,146,147,148,149,150,151^ were assessed as at high risk of bias, 6 trials^34,37,55,61,133,138^ as of some concern, and 2 trials^86,118^ as at low risk of bias.

### Choice of Prior

Based on models using different priors, we chose prior 0 to 5 for TJCi, TJC, SJC, DAS28i, and TJC28i; prior 0 to 6 for ESR; and prior 0 to 200 for CRP level. The density plot and sensitivity analysis with a 0 to 20 prior supported this decision.

### Primary TJCi Outcome, Unadjusted Analysis

We imputed 14 missing TJC values (10.6% of all primary outcomes; eMethods 5 in Supplement 1) to obtain a complete data set of 132 studies^22,23,24,25,26,27,28,29,30,31,32,33,34,35,36,37,38,39,40,41,42,43,44,45,46,47,48,49,50,51,52,53,54,55,56,57,58,59,60,61,62,63,64,65,66,67,68,69,70,71,72,73,74,75,76,77,78,79,80,81,82,83,84,85,86,87,88,89,90,91,92,93,94,95,96,97,98,99,100,101,102,103,104,105,106,107,108,109,110,111,112,113,114,115,116,117,118,119,120,121,122,123,124,125,126,127,128,129,130,131,132,133,134,135,136,137,138,139,140,141,142,143,144,145,146,147,148,149,150,151^ with TJC outcomes (eResults 2 in Supplement 1). Differences in estimates vs methotrexate between fixed- and random-effects models were minimal. For instance, the reduction in TJCi outcome of methotrexate vs placebo was −4.87 joints (95% CrI, −5.74 to −4.00 joints) with the fixed-effects model and −4.90 joints (95% CrI, −6.12 to −3.72 joints) with the random-effects model (eTable 2 in Supplement 1). A random-effects NMA was selected based on deviation information criterion, with a between-study SD of 1.21 (95% CrI, 0.65 to 1.83). A sensitivity analysis of TJC without imputations showed results generally not different from TJCi. For instance, the reduction in TJC outcome of methotrexate vs placebo was −4.90 joints (95% CrI, −6.12 to −3.72 joints) with imputations (TJCi) and −4.83 joints (95% CrI, −6.07 to −3.62 joints) without imputations (TJC) (eTable 3 in Supplement 1).

### Effect Modifier Analysis

See eResults 3 and eFigure 1 in Supplement 1. Apart from mean baseline DAS28 with imputed values (132 studies^22,23,24,25,26,27,28,29,30,31,32,33,34,35,36,37,38,39,40,41,42,43,44,45,46,47,48,49,50,51,52,53,54,55,56,57,58,59,60,61,62,63,64,65,66,67,68,69,70,71,72,73,74,75,76,77,78,79,80,81,82,83,84,85,86,87,88,89,90,91,92,93,94,95,96,97,98,99,100,101,102,103,104,105,106,107,108,109,110,111,112,113,114,115,116,117,118,119,120,121,122,123,124,125,126,127,128,129,130,131,132,133,134,135,136,137,138,139,140,141,142,143,144,145,146,147,148,149,150,151^), covariates did not improve model fit significantly (eTable 4 in Supplement 1).

### Primary TJCi Outcome Adjusted for Baseline DAS28 With Peroral Methotrexate and Placebo as Reference

The primary TJCi outcome adjusted for baseline DAS28 was found among 132 trials^22,23,24,25,26,27,28,29,30,31,32,33,34,35,36,37,38,39,40,41,42,43,44,45,46,47,48,49,50,51,52,53,54,55,56,57,58,59,60,61,62,63,64,65,66,67,68,69,70,71,72,73,74,75,76,77,78,79,80,81,82,83,84,85,86,87,88,89,90,91,92,93,94,95,96,97,98,99,100,101,102,103,104,105,106,107,108,109,110,111,112,113,114,115,116,117,118,119,120,121,122,123,124,125,126,127,128,129,130,131,132,133,134,135,136,137,138,139,140,141,142,143,144,145,146,147,148,149,150,151^ (eResults 4 in Supplement 1). Outcomes with peroral methotrexate as the reference are shown in the Table and eFigure 2 in Supplement 1; eTable 5 in Supplement 1 shows results with placebo as the reference. The methotrexate vs placebo outcome for reduction in TJCi was 5.18 joints (95% CrI, 4.07 to 6.28 joints) (Table), marginally higher than the unadjusted outcome (eTable 3 in Supplement 1). Cyclophosphamide was favorable compared with methotrexate in terms of reduction in TJCi (6.08 joints; 95% CrI, 0.44 to 11.66 joints), while remaining drugs and glucocorticoid (−2.54 joints; 95% CrI, −5.16 to 0.08 joints) were associated with similar or lower tender joint counts (Table). No drugs were unfavorable compared with placebo (eTable 5 in Supplement 1). Leflunomide, cyclosporine, injected gold, tacrolimus, iguratimod, dapsone, glucocorticoid, and injectable methotrexate were favorable compared with placebo and did not differ substantially from peroral methotrexate. Sulfasalazine, chloroquine, d-penicillamine, azathioprine, oral gold, and levamisole were favorable compared with placebo but unfavorable compared with methotrexate. *Tripterygium wilfordii*, timegadine, bucillamine, tiopronin, pyritinol, mycophenolate mofetil, OM-8980, lobenzarit, reumacon, prospidin, aminopterin, and eazmov plus had numerically higher but overlapping outcomes compared with placebo. Comparisons of all interventions vs each other are presented in eTable 6 in Supplement 1.

### Ranking

Rankograms for all treatments are presented in eFigure 3 in Supplement 1, arranged in the order assessed by visual inspection. The most effective treatments were cyclophosphamide, tacrolimus, and oral and injected methotrexate. Placebo was poorest but not worse than lobenzarit, mycophenolate mofetil, rheumacon, timegadine, or eazmov plus.

### Exploration of Inconsistency

See eResults 5 in Supplement 1. We compared estimates in the NMA model with those from an unrelated mean effect sizes model. We did not find meaningful differences in any credible interval. The dev-dev plot is shown in eFigure 4 in Supplement 1. Model fit data are shown in eTable 7 in Supplement 1.

### Sensitivity Analyses for Outcomes Adjusted for DAS28

DAS28-adjusted results of TJC, SJC, ESR, CRP, TJC28i, and DAS28i are shown in the Table (peroral methotrexate as reference) and eTable 5 in Supplement 1 (placebo as reference). For TJC (118 studies,^23,26,28,29,30,34,36,37,38,39,40,41,42,43,44,45,46,47,48,49,50,51,52,53,54,55,56,57,58,59,60,61,62,63,64,66,67,68,69,70,71,72,73,74,75,76,77,79,80,81,82,83,84,85,87,88,89,92,93,94,95,96,97,98,99,100,101,102,103,104,105,106,108,109,110,111,112,113,114,115,116,117,118,119,120,121,122,123,124,125,126,127,128,129,130,131,132,133,134,135,136,137,138,139,140,141,142,143,144,145,146,147,148,149,150,151^ 28 treatments), unlike in the primary analysis, mycophenolate mofetil was no longer unfavorable compared with methotrexate but still not different from placebo. GC was unfavorable compared with methotrexate but still favorable compared with placebo. For SJC (97 studies,^22,26,29,30,31,32,33,37,39,40,41,44,45,46,50,51,52,54,57,58,60,62,63,65,67,68,69,70,71,72,73,74,75,76,77,78,80,81,82,83,84,86,87,88,92,93,94,96,97,98,100,101,102,103,105,107,108,109,110,111,113,114,115,116,118,119,120,122,123,124,125,127,128,129,130,131,132,133,134,135,137,138,139,140,141,142,143,144,145,146,147,148,149,150,151^ 27 treatments), unlike in the primary analysis, *Tripterygium* was favorable compared with placebo and GC was not different from placebo. For ESR (116 studies,^22,23,24,25,26,27,28,29,30,32,34,35,36,37,38,39,40,41,42,43,44,45,46,47,48,49,50,51,52,53,54,55,56,57,58,59,60,61,62,63,64,65,66,67,68,69,70,71,72,73,74,75,76,77,78,80,81,82,84,85,87,88,89,90,91,92,93,94,96,97,98,99,100,101,104,105,106,107,108,110,111,112,113,114,115,116,117,118,119,120,121,122,123,124,125,127,128,129,135,136,137,140,141,142,143,144,146,147,148,149,150,151^ 28 treatments), in contrast to the primary analysis, cyclosporine and azathioprine were not different compared with placebo. Furthermore, *Tripterygium*, timegadine, tiopronin, pyritinol, and prospidin were favorable compared with placebo and not different compared with methotrexate. For CRP (47 studies,^40,45,56,58,64,66,67,79,85,87,95,97,98,100,104,110,111,113,114,115,116,117,122,124,125,126,127,128,129,131,132,133,134,136,137,138,140,141,142,144,146,147,148,149,150,151^ 20 treatments), chloroquine, D-penicillamine, azathioprine, oral gold, dapsone, and GC were not different compared with placebo, whereas prospidin was favorable compared with placebo and not different compared with methotrexate. For DAS28i, injectable methotrexate was favorable compared with oral methotrexate. Mycophenolate mofetil, OM-8980, lobenzarit, reumacon, and eazmov plus were not different from placebo, whereas all other drugs were favorable compared with placebo and not different from or unfavorable compared with methotrexate. For TJC28i,unlike with TJCi, cyclophosphamide, levamisole, and mycophenolate mofetil were not different from methotrexate.

### Additional Analyses

Outcomes performed with a model using prior distribution 0 to 20 are shown in eTable 8 in Supplement 1. A sensitivity analysis separating chloroquine and hydroxychloroquine in 2 nodes showed similar outcomes in the 2 groups (eResults 6 in Supplement 1).

## Discussion

Results from this efficacy meta-analysis support existing guidelines.^5,160^ Study information on reasons for dropping out (eg, toxic effects, lack of effect, violation of study criteria, and no appearance) varied too much to be standardized. Dropout rates, which reflect tolerability and toxic effects, were instead reported.

### Summary of Main Results

GC and well-defined csDMARDs (methotrexate, sulfasalazine, leflunomide, cyclosporine, gold, chloroquine, D-penicillamine, azathioprine, levamisole, and cyclophosphamide) were all favorable compared with placebo, but sulfasalazine, chloroquine, D-penicillamine, azathioprine, peroral gold, levamisole, and GC were unfavorable compared with methotrexate. Outcomes of chloroquine and hydroxychloroquine were similar. Cyclophosphamide was favorable compared with methotrexate. In addition, noncurrent practice drugs tacrolimus, iguratimod, and dapsone had outcomes comparable with those of established csDMARDs.

### Overall Completeness and Applicability

We avoided exclusions of questionable csDMARDs because such procedures may exclude relevant comparators. Cyclophosphamide can be used to treat rheumatoid vasculitis and pulmonary fibrosis, and under such conditions an associated improvement in arthritis is valuable. However, the risk of leukemia increases when the accumulated dose of cyclophosphamide exceeds 36 g.^161^ Thus, the general applicability of cyclophosphamide is limited. Historically, the outcome of gold treatment is interesting, although gold is no longer a current practice drug. Tacrolimus had a high dropout rate and is as expensive as tDMARDs but may be suitable for patients with RA who have had organ transplants. In patients with limited insurance coverage^162^ and those from resource-limited regions with limited access to expensive tDMARDs, csDMARDs are important, especially as a combination of 2 to 3 inexpensive csDMARDs may be as effective as tDMARDs.^16,17,163,164,165^ However, even in such patients, gold, D-penicillamine, azathioprine, and levamisole may have limited, if any, applicability given high dropout rates of 33% to 39% identified in our study.

### Quality of Evidence

Generally, evidence from RCTs is of high quality. Concerning placebo and most previously established treatments (methotrexate, sulfasalazine, leflunomide, ciclosporin, gold, D-penicillamine, azathioprine, chloroquine, and GC), the number of studies and participants was adequate for a robust conclusion. However, outcomes for remaining drugs were based on 1 to 3 studies and therefore less reliable. For instance, the favorable evidence for cyclophosphamide was weak given that 41 participants were included.^29,31,37^ Some drugs may deserve consideration; for instance, iguratimod, which had a dropout rate of 12.4%, may be the most promising of these agents. Models using different priors gave similar results in accordance with robust outcomes.

### Agreement and Disagreements With Other Reviews

Previously, in a conventional meta-analysis^16^ of 33 mono-csDMARD RCTs, we found that injectable gold, sulfasalazine, methotrexate, leflunomide, and cyclosporine were associated with similar radiographic outcomes and that D-penicillamine, chloroquine, oral gold, azathioprine, and cyclophosphamide were associated with inferior outcomes compared with methotrexate but superior outcomes compared with placebo. Over the last 20 years, several conventional meta-analyses have investigated outcomes associated with individual csDMARDs.^166,167,168,169,170,171,172,173,174,175^ All investigated drugs were superior to placebo, but in these analyses, drugs were not compared vs each other.

### Limitations

This study has several limitations. Clinical heterogeneity across studies may have biased outcomes. However, several univariate sensitivity analyses disclosed only 1 factor associated with the outcome (baseline DAS28), and by adjusting the outcome for DAS28, we tried to limit this bias.

The lack of official reporting guidelines^176^ and protocol registers^177^ when most of these studies were performed may explain insufficient reporting of scientific procedures in the studies. This may have contributed to a high, potentially exaggerated risk of bias assessment and to variability in presentation of outcomes, which made imputations necessary in 14 primary outcomes (10.6%).^22,24,25,27,31,32,33,35,65,78,86,90,91,107^ However, the TJC full analysis with imputed values showed similar results as the TJC analysis based on measured values.

A general problem with NMA is that many comparisons are based on an assumption of transitivity, meaning that if an association between a first and second drug holds and it also holds between the second and third drug, it is assumed to necessarily hold between the first and third drug. We tested this by the unrelated mean effect sizes model, which did not reveal important inconsistency. That is, it did not disclose differences between direct and indirect comparisons, suggesting that the assumption of transitivity was acceptable.

Undoubtedly, potential drugs have been tested in unpublished studies,^178^ and we did not include antibiotics, such as tetracycline, associated with potential csDMARD outcomes.^179^ Despite these reservations, to our knowledge this analysis is the largest integrated mono-csDMARD analysis hitherto presented, and we believe that the risk of having overlooked important studies is small. Sensitivity analyses produced fewer significant differences, but numerically they were generally not in opposition to the full analysis.

## Conclusions

This meta-analysis adds a comprehensive analysis comparing outcomes of multiple csDMARDs to each other in 1 integrated analysis. Currently established csDMARDs (methotrexate, leflunomide, cyclosporine, sulfasalazine, and [hydroxy]chloroquine) were found to be associated with disease-modifying outcomes in RA. The significant outcomes of former but now abandoned drugs, such as cyclophosphamide, gold, azathioprine, D-penicillamine, and levamisole, were not sufficient to justify a reintroduction considering high dropout rates. Some unapproved drugs showed outcomes potentially justifying further investigation. Outcomes confirmed the role of methotrexate as primary reference csDMARD.

## Notes

Supplement 1. **eMethods 1.** Eligibility Criteria **eMethods 2.** Information Sources and Search **eMethods 3.** Study Selection and Data Collection **eMethods 4.** Data Items **eMethods 5.** Estimations of Missing Values and Conversion Factors **eMethods 6.** Risk of Bias Within Individual Studies **eMethods 7.** Planned Method of Analysis **eMethods 8.** Effect Modifier Analysis **eResults 1.** Study Selection **eResults 2.** Primary Outcome (Complete Analysis) With Methotrexate as Reference, Unadjusted Analysis **eResults 3.** Effect Modifier Analyses **eResults 4.** Primary Outcome With Methotrexate as Reference, Adjusted for Baseline Disease Activity Score 28 (DAS28) **eResults 5.** Exploration of Inconsistency **eResults 6.** Results of Additional Analyses
eReferences.
**eTable 1.** Baseline Characteristics, Primary Outcome and Bias Score of Included Studies **eTable 2.** Unadjusted Tender Joint Count Imputed (TJCi) Analysis Using Fixed- and Random-Effects Models **eTable 3.** Tender Joint Count Imputed (TJCi) Sensitivity Analysis Comparing Analyses With and Without Imputed Values **eTable 4.** Model Fit Parameters for Tender Joint Count Imputed (TJCi) Analyses Including and Excluding Effect Modifiers in Metaregression Analyses **eTable 5.** Primary Outcome and Sensitivity Outcomes With Placebo as Reference **eTable 6.** Comparisons of Tender Joint Count Imputed (TJCi) Outcome of All Interventions vs All Interventions **eTable 7.** Model Fit Data for Primary Outcome and Sensitivity Outcomes **eTable 8.** Primary and Sensitivity Outcomes Calculated With Prior 0-20 **eFigure 1.** Subgroup Analyses **eFigure 2.** Forest Plot of Primary Tender Joint Count Imputed (TJCi) Outcomes vs Peroral Methotrexate **eFigure 3.** Rankograms of 29 Tested Interventions **eFigure 4.** dev-dev Plot of Unrelated Mean Effects Mean vs Network Meta-Analyses Mean

Supplement 2.
Data Sharing Statement
