# Supplementary material for: Monotreatment With Conventional Antirheumatic Drugs or Glucocorticoids in Rheumatoid Arthritis: A Network Meta-Analysis
Source: JAMA Netw Open. 2023 Oct 6;6(10):e2335950. doi: 10.1001/jamanetworkopen.2023.35950 (PMC10559183; doi:10.1001/jamanetworkopen.2023.35950)
Supplement: Supplement 1. — eMethods 1. Eligibility Criteria eMethods 2. Information Sources and Search eMethods 3. Study Selection and Data Collection eMethods 4. Data Items eMethods 5. Estimations of Missing Values and Conversion Factors eMethods 6. Risk of Bias Within Individual Studies eMethods 7. Planned Method of Analysis eMethods 8. Effect Modifier Analysis eResults 1. Study Selection eResults 2. Primary Outcome (Complete Analysis) With Methotrexate as Reference, Unadjusted Analysis eResults 3. Effect Modifier Analyses eResults 4. Primary Outcome With Methotrexate as Reference, Adjusted for Baseline Disease Activity Score 28 (DAS28) eResults 5. Exploration of Inconsistency eResults 6. Results of Additional Analyses eReferences. eTable 1. Baseline Characteristics, Primary Outcome and Bias Score of Included Studies eTable 2. Unadjusted Tender Joint Count Imputed (TJCi) Analysis Using Fixed- and Random-Effects Models eTable 3. Tender Joint Count Imputed (TJCi) Sensitivity Analysis Comparing Analyses With and Without Imputed Values eTable 4. Model Fit Parameters for Tender Joint Count Imputed (TJCi) Analyses Including and Excluding Effect Modifiers in Metaregression Analyses eTable 5. Primary Outcome and Sensitivity Outcomes With Placebo as Reference eTable 6. Comparisons of Tender Joint Count Imputed (TJCi) Outcome of All Interventions vs All Interventions eTable 7. Model Fit Data for Primary Outcome and Sensitivity Outcomes eTable 8. Primary and Sensitivity Outcomes Calculated With Prior 0-20 eFigure 1. Subgroup Analyses eFigure 2. Forest Plot of Primary Tender Joint Count Imputed (TJCi) Outcomes vs Peroral Methotrexate eFigure 3. Rankograms of 29 Tested Interventions eFigure 4. dev-dev Plot of Unrelated Mean Effects Mean vs Network Meta-Analyses Mean [file jamanetwopen-e2335950-s001.pdf]

Supplemental Online Content

Guski LS, Jürgens G, Pedder H, et al. Mono treatment with conventional antirheumatic drugs or glucocorticoids in rheumatoid arthritis: a network meta-analysis. *JAMA Netw Open*. 2023;6(6):e2335950.
doi:10.1001/jamanetworkopen.2023.35950

eMethods 1. Eligibility Criteria

eMethods 2. Information Sources and Search

eMethods 3. Study Selection and Data Collection

eMethods 4. Data Items

eMethods 5. Estimations of Missing Values and Conversion Factors

eMethods 6. Risk of Bias Within Individual Studies

eMethods 7. Planned Method of Analysis

eMethods 8. Effect Modifier Analysis

eResults 1. Study Selection

eResults 2. Primary Outcome (Complete Analysis) With Methotrexate as Reference, Unadjusted Analysis

eResults 3. Effect Modifier Analyses

eResults 4. Primary Outcome With Methotrexate as Reference, Adjusted for Baseline Disease Activity Score 28 (DAS28)

eResults 5. Exploration of Inconsistency

eResults 6. Results of Additional Analyses

eReferences.

eTable 1. Baseline Characteristics, Primary Outcome and Bias Score of Included Studies

eTable 2. Unadjusted Tender Joint Count Imputed (TJCi) Analysis Using Fixed- and Random-Effects Models

eTable 3. Tender Joint Count Imputed (TJCi) Sensitivity Analysis Comparing Analyses With and Without Imputated Values

eTable 4. Model Fit Parameters for Tender Joint Count Imputed (TJCi) Analyses Including and Excluding Effect Modifiers in Metaregression Analyses

eTable 5. Primary Outcome and Sensitivity Outcomes With Placebo as Reference

eTable 6. Comparisons of Tender Joint Count Imputed (TJCi) Outcome of All Interventions vs All Interventions

eTable 7. Model Fit Data for Primary Outcome and Sensitivity Outcomes

© 2023 Guski LS et al. *JAMA Network Open.*

eTable 8. Primary and Sensitivity Outcomes Calculated With Prior 0-20

eFigure 1. Subgroup Analyses

eFigure 2. Forest Plot of Primary Tender Joint Count Imputed (TJCi) Outcomes vs Peroral Methotrexate

eFigure 3. Rankograms of 29 Tested Interventions

eFigure 4. dev-dev Plot of Unrelated Mean Effects Mean vs Network Meta-Analyses Mean

This supplemental material has been provided by the authors to give readers additional information about their work.

eMethods 1. Eligibility Criteria

1.1 Inclusion criteria

- *Types of studies:* Open, single blind or double blind randomized controlled trials of RA patients published in English.
- *Types of participants*: Patients above 15 years with RA according to the criteria defined by the authors of the article.
- *Types of interventions:* Any csDMARD, unless deregistered due to toxicity, glucocorticoid, placebo or comparator without anti-inflammatory effect, which could be 1) no treatment, 2) pain-relieving treatment including non-steroid anti-inflammatory drugs, or 3) very-low dose of the investigated DMARD. Chloroquine and hydroxychloroquine were merged into one node. No other DMARDs or GC were merged into the same node, but placebo and comparators without systemic anti-inflammatory effects were intended to be merged into the placebo node unless a sensitivity analysis would show different effects between them. Concomitant treatment with drugs with no anti-inflammatory effects are allowed, even if the drugs are not balanced between the intervention group and the comparator group, provided that the drug does not interact with the tested DMARD. Concomitant GC treatment in DMARD studies, which existed at inclusion and was not changed during the study, was allowed.
- *Types of outcome measures:* Relevant studies had to report outcomes on at least one of the following variables: TJC, SJC, ESR and CRP.
- *Duration of intervention:* In relevant studies, the duration of the intervention had to be at least 12 weeks for DMARDs and at least 2 weeks for GC in the randomized phase.

1.2 Exclusion criteria

- Concomitant DMARD treatment in an intervention group randomized to a csDMARD or GC treatment (combination therapy)
- Enriched design, defined as studies where participants were known responders or non-responders to one of the study drugs.

eMethods 2. Information Sources and Search

Electronic sources include MEDLINE, Cochrane Central, Embase and Clinicaltrials.gov. The search was conducted on 13th of June 2018 and updated on the 12th of January 2022 and restricted to published peer-reviewed full-length articles in English but not restricted by any time constraints. We intended to search all RCTs of RA on the effect of any DMARD, GC and combinations of these and from this pool of RCTs to select those dealing with mono DMARD treatment and mono-GC treatment. The electronic search string can be found in the present appendix, section 3. Clinical trials.gov was searched for completed interventional studies of rheumatoid arthritis with results. Finally, we hand searched reference lists of included studies and previous meta-analyses. We conducted a post-hoc screening in PubMed on September 15th, 2022.

Search string

(("antirheumatic agents"[Pharmacological Action] OR ("antirheumatic agents"[MeSH Terms] OR "biological factors"[MeSH Terms])) OR ((((((((("antirheumatic agents"[Pharmacological Action] OR "antirheumatic agents"[MeSH Terms] OR ("antirheumatic"[All Fields] AND "agents"[All Fields]) OR "antirheumatic agents"[All Fields] OR "antirheumatic"[All Fields]) AND agents[All Fields]) OR (("biology"[MeSH Terms] OR "biology"[All Fields] OR "biological"[All Fields]) AND factors[All Fields])) OR (("biology"[MeSH Terms] OR "biology"[All Fields] OR "biologic"[All Fields]) AND agent[All Fields])) OR (("antirheumatic agents"[Pharmacological Action] OR "antirheumatic agents"[MeSH Terms] OR ("antirheumatic"[All Fields] AND "agents"[All Fields]) OR "antirheumatic agents"[All Fields] OR "antirheumatic"[All Fields]) AND agent[All Fields])) OR (("antirheumatic agents"[Pharmacological Action] OR "antirheumatic agents"[MeSH Terms] OR ("antirheumatic"[All Fields] AND "agents"[All Fields]) OR "antirheumatic agents"[All Fields] OR "antirheumatic"[All Fields]) AND ("pharmaceutical preparations"[MeSH Terms] OR ("pharmaceutical"[All Fields] AND "preparations"[All Fields]) OR "pharmaceutical preparations"[All Fields] OR "drugs"[All Fields]))) OR (("antirheumatic agents"[Pharmacological Action] OR "antirheumatic agents"[MeSH Terms] OR ("antirheumatic"[All Fields] AND "agents"[All Fields]) OR "antirheumatic agents"[All Fields] OR "antirheumatic"[All Fields]) AND drug[All Fields])) OR (((((("antirheumatic"[All Fields] AND "agents"[All Fields]) OR ("biological"[All Fields] AND "factors"[All Fields])) OR ("biologic"[All Fields] AND "agent"[All Fields])) OR ("antirheumatic"[All Fields] AND "agent"[All Fields])) OR ("antirheumatic"[All Fields] AND

© 2023 Guski LS et al. *JAMA Network Open.*

"drugs"[All Fields])) OR ("antirheumatic"[All Fields] AND "drug"[All Fields]))) OR (((((("antirheumatic agents"[All Fields] OR "biological factors"[All Fields]) OR "biologic agent"[All Fields]) OR "biological agent"[All Fields]) OR "antirheumatic agent"[All Fields]) OR "antirheumatic drugs"[All Fields]) OR "antirheumatic drug"[All Fields])) OR ((((((("antirheumatic agents"[Pharmacological Action] OR "antirheumatic agents"[MeSH Terms] OR ("antirheumatic"[All Fields] AND "agents"[All Fields]) OR "antirheumatic agents"[All Fields] OR "dmard"[All Fields]) OR bdmard[All Fields]) OR sdmard[All Fields]) OR csdmard[All Fields]) OR tsdmard[All Fields]) OR ("antirheumatic agents"[Pharmacological Action] OR "antirheumatic agents"[MeSH Terms] OR ("antirheumatic"[All Fields] AND "agents"[All Fields]) OR "antirheumatic agents"[All Fields] OR "antirheumatic"[All Fields])) OR ("antirheumatic agents"[Pharmacological Action] OR "antirheumatic agents"[MeSH Terms] OR ("antirheumatic"[All Fields] AND "agents"[All Fields]) OR "antirheumatic agents"[All Fields] OR "antirheumatics"[All Fields])))) AND ("arthritis, rheumatoid"[MeSH Terms] OR ((((rheumatoid[All Fields] AND ("arthritis"[MeSH Terms] OR "arthritis"[All Fields])) OR (("arthritis"[MeSH Terms] OR "arthritis"[All Fields]) AND rheumatoid[All Fields])) OR "rheumatoid arthritis"[All Fields]) OR (("arthritis"[All Fields] AND "rheumatoid"[All Fields]) OR ("rheumatoid"[All Fields] AND "arthritis"[All Fields])))) AND ((Clinical Trial[ptyp] OR Clinical Trial, Phase II[ptyp] OR Clinical Trial, Phase III[ptyp] OR Controlled Clinical Trial[ptyp] OR Pragmatic Clinical Trial[ptyp] OR Randomized Controlled Trial[ptyp]) AND "humans"[MeSH Terms]) AND (English[lang])

eMethods 3. Study Selection and Data Collection

Given the complexity of the interventions being investigated, we categorized the included interventions first at the level of full article assessment according to a predefined procedure according to which studies were excluded with a reason or included and categorized according to the type of intervention. The reasons for exclusion could be

1) the study could not with certainty be classified as an RCT
2) no separate RA-data were reported
3) the intervention could not be classified as a DMARD or glucocorticoid
4) none of the predefined outcomes were measured
5) the study was an extension study in which randomization had been repealed, and/or intervention changes allowed
6) the drug-design was enriched, i.e., one of the drugs to which the patients were randomized was already known to be effective or ineffective in that group of patients

The included studies were categorized according to the type of intervention in the following groups:

1) mono-csDMARD treatment including mono-GC studies
2) combination- csDMARD treatment including combinations with GC
3) TNF inhibitor studies
4) IL-6 inhibitor studies
5) B-cell inhibitor studies
6) T-cell inhibitor studies
7) JAK-inhibitor studies
8) other, which could be new interventions appearing during the procedure or rare interventions not generally used.

Two authors pairwise (LSG/NG and GJ/SEA) independently and blinded to each other´s decisions selected studies. Any disagreements were solved by agreement between the two authors. Remaining disagreements were solved at a final meeting between all 4 authors.

Data were recorded on a standardized extraction form using Excel spreadsheet software from Microsoft. Data were collected by a single author (LSG). All data were independently controlled by two authors (NKGL and NG). Any disagreements were solved by discussion between LSG and NG.

eMethods 4. Data Items

Baseline mean values (SD) and post treatment mean values (SD) of outcomes TJC, SJC, ESR and CRP were recorded. In addition, the following baseline variables for each treatment arm were recorded: mean age, female percentage, percent rheumatoid factor positivity, and time since diagnosis. It was the intention also to extract disease activity score (DAS) and health assessment questionnaire score (HAQ). We also recorded whether GC was allowed during the study and the GC dose, and whether the patients previously had been exposed to DMARDs and the adequacy of a previous DMARD response.

© 2023 Guski LS et al. *JAMA Network Open.*

In case of repeated publications, we used the publication with the most complete data fulfilling the eligibility criteria, but additional publications were used to provide missing data if available.
Methods required to prepare the data for presentation or synthesis, such as handling of missing summary statistics, or data conversions are explained in detail in eMethods 5. Specifically, missing SJC, TJC, ESR or CRP values were imputed. If TJC and SJC were assessed on more than 28 joints, TJC and SJC were converted to on an assessment of 28 joints (TJC28 and SJC28). Then we calculated approximated values for baseline DAS28 in studies, where DAS28 was not reported.

eMethods 5. Estimations of Missing Values and Conversion Factors

5.1 Contact to authors
As 85% of the studies were more than 20 years old, we generally did not contact authors for supplementary information. We did so on 5 occasions; two authors responded with additional information; one author responded but was unable to provide additional information and 2 authors did not respond.

5.2 Estimation of missing SD values
If SD was not reported it was estimated from an SE, a CI, a p-value, a t-value, a 25-75% percentile range divided by1,35 as suggested in the Cochrane handbook (1), or from a full range multiplied by conversion factor from table 1 by Walther and Yao (2). If SD was reported for the baseline value, but not for the post-treatment value and the post-treatment value could not be estimated as described above, we imputed the SD/mean quotient for the baseline value and multiplied this with the post-treatment value to obtain an estimate of the post-treatment SD.
If an sd for one intervention group could be estimated by means of a specified significant p-value, but not for another intervention group because it was reported as "NS" (non-significant), we used the estimated sd from the significant group in the non-significant group.
If an sd estimated by means of a conversion factor gave a significant result for the individual study, which was in contrast to a reported non-significant result, we relatively and pairwise imputed the sd to a level resulting in $p > 0.05$.
Finally, we imputed the average of SD/mean for each study outcome and used this value as a conversion factor to calculate unreported SDs which could not be estimated by one of the mentioned methods. For SJC, we calculated this conversion factor to be 0,51 for baseline values and 0,82 for post treatment values, for TJC be 0,52 for baseline values and 0,77 for post treatment values, for ESR to be 0,53 for baseline values and 0,66 for post treatment values, and for CRP to be 0,89 for baseline values and 1,10 for post treatment values.

5.3 Conversion of median duration to mean duration
If time of duration of RA was reported for both time since diagnosis and time since debut of symptoms, the time since diagnosis was recorded. We recorded mean duration. Some studies reported both mean and median duration. We used these studies to estimate conversion factors, which we could use to translate median duration to mean duration in studies, which only reported median duration
Six studies presented both mean and median duration in 9 treatment groups. In one study with an upper duration limit of 5 years at inclusion (3) there were only small differences in two treatment arms between mean duration (35 and 28 months) and median duration (34 and 31 months). Thus, in studies with a maximum duration of 5 years at inclusion, we translated median duration directly to mean duration (conversion factor 1).In one study with an upper duration limit of 10 years at inclusion (4) there were differences in 3 treatment arms between mean duration (21.6, 19.2 and 22.8 months) and median duration (12, 12 and 12 months). The mean ratio of 21.6/12, 19.2/12 and 22.8/12 was 1,75. Thus, in studies with a maximum duration of 5-10 years at inclusion, we translated median duration to mean duration by a conversion factor of 1,75.In three studies with no upper limit of disease duration at inclusion and a median disease duration of 36 months or less (5-7) there were differences in 7 treatment arms between mean duration (25.9, 21.9, 72,5, 68,2, 64, 55 and 75 months) and median duration (11.5, 11.2, 35.0, 36.0, 36.0, 24.0 and 36.0 months). The mean ratio of 25.9/11.5, 21.9/11.2, 72.5/35, 68.2/36, 64/36, 55/24 and 75/36 was 2.04. Thus, in studies with no maximum duration at inclusion and a median duration of 3 years or less, we translated median duration to mean duration by a conversion factor of 2.0.In one study with no upper limit of disease duration at inclusion and a median disease duration of 72 months (8) there were differences in 2 treatment arms between mean duration (8.3 and 8.5 years) and median duration (6,0 and 6,5 years). The mean ratio of 8,3/6 and 8,5/6,5 was 1,35. Thus, in studies with no maximum duration and a median duration of at least 6 years, we translated median duration to mean duration by a conversion factor of 1,35.

In studies with no maximum duration and a median duration between 3 and 6 years we interpolated conversion factors as shown in the below eTable A.
eTable A: Conversion factor of median to mean duration

© 2023 Guski LS et al. *JAMA Network Open.*

**Duration**	**Median duration**	**Conversion Factor to mean duration**
**Max 5 y**	**0-5**	**1,0**
**Max 6-10 y**	**0-10**	**1,75**
**No max**	**0-3**	**2,0**
**No max**	**3,1-3,5**	**1,9**
**No max**	**3,6-4,0**	**1,79**
**No max**	**4,1-4,5**	**1,68**
**No max**	**4,6-5,0**	**1,57**
**No max**	**5,1-5,5**	**1,46**
**No max**	**5,6-**	**1,35**

Four studies did not give information about duration. One study (9) reported that 68% in one arm and 87% in the other arm had joint erosions and based on this information the mean duration was estimated to be 3 years (36 months) and 5 years (60 months) (10). One study (11) reported that 67% in one arm and 61% in the other arm had joint erosions and based on this information the mean duration was estimated to be 3 years (36 months) and 2,5 years (30 months) (10). In one study (12), all patients had erosions and the mean duration was accordingly estimated to be 7 years (84 months) (10).
One study (13) reported that only 25% of patients had been on DMARDS and 15% of patients had been on GC before inclusion in the study. We therefore estimated that the mean disease duration was relatively short and imputed a value of 12 months.

5.4 Conversion of median age to mean age
Two studies reported both mean and median age. The difference was small, varying between 0 and 2 years in four treatment arms. Consequently, we used the median age as mean age, when only the median age was reported.

5.5 Conversion of Ritchie Index to tender joint count (TJC).
In case that the total number of joints included in the calculation of the Ritchie index was not reported we assumed that it was based on evaluation of 53 joints as in the original definition of the Ritchie index. Considering that the Ritchie index consists of 4 evaluations of 0, 1, 2, and 3, we assumed that the mean Ritchie of an evaluated joint would be 1.5 ((0+1+2+3)/4). In 14 studies, which evaluated both Ritchie index and TJC, the relation between TJC and Ritchie was as shown in eTable B, confirming that a conversion factor of 1.5 was suitable.

eTable B: Relationship between TJC and Ritchie index in 14 studies

5.00	7.50	**1.5**
8.00	9.00	**1.13**
17.00	25.00	**1.47**
16.00	21.00	**1.31**
14.00	20.00	**1.43**
14.00	24.00	**1.71**
14.50	24.00	**1.66**
14.00	22.00	**1.57**
13.00	19.00	**1.46**
12.00	22.00	**1.83**
13.00	20.00	**1.54**
11.00	18.50	**1.68**
8.10	12.60	**1.48**
11.20	14.40	**1.27**
Mean		**1.503**

5.6 Estimation of missing TJC values from SJC or ESR
The most completely recorded outcome was TJC (119 studies), which therefore was chosen as the outcome to be complete. In studies which did not report either TJC directly or Ritche index, we used other efficacy measures to estimate TJC. Primarily, we used SJC values as a direct substitute for missing TJC values (8 studies) and secondarily we transformed ESR values (5 studies). We calculated the TJC/ESR ratio for all studies

© 2023 Guski LS et al. *JAMA Network Open.*

reporting both ESR and TJC to be 0.39. We used this constant to transform ESR values to TJC values for the 5 studies, which had ESR, but no joint count as an outcome.
We used the inverse value (1/0,39 =2,56) to transform TJC values to ESR values for the purpose of estimating DAS28 values.

5.7 Conversion of median Ritchie index, median TJC, and median SJC to mean values
In five treatment arms of two studies (14) median and mean Ritchie were similar, and in four treatment arms of two studies (15, 16) median and mean joint counts were similar, and therefore we used the median value of these variables as mean values, when only the median value was reported.

5.8 Conversion of median ESR to mean ESR
We used ESR data from one of our previous publications ("Denmark 2000")(17) to calculate the relation between median and mean ESR **(eTable C).** The weighted mean value from table C is also shown in table D ("Denmark 2000").
In addition, we used the terms "mean erythrocyte sedimentation rate and median erythrocyte sedimentation and rheumatoid arthritis", which revealed 103 articles in PubMed. We identified 2 studies (18,19), which showed data on both mean and median ESR from 3 populations **(eTable D).**
Consequently, we used the mean weighted value of 1.23 from these 4 populations as a conversion factor of median ESR to mean ESR **(eTable D)**.

eTable C: ESR mean and median values from a cohort of 112 RA patients (Denmark 2000) (17).

Year	N	ESR. mean	ESR Median	Me/Md
0	112	39.20	31.50	1.24
1	112	19.30	14.50	1.33
2	112	20.20	15.00	1.35
3	112	22.30	15.50	1.44
4	109	25.10	17.00	1.48
5	104	26.00	20.00	1.30
6	99	25.40	18.00	1.41
7	90	27.10	23.00	1.18
8	82	31.90	25.50	1.25
9	70	30.60	23.50	1.30
10	62	34.10	30.00	1.14
11	55	31.50	27.00	1.17
12	42	32.50	28.00	1.16
13	36	35.90	31.00	1.16
Weighted mean				1.30

eTable D: ESR mean and median in cohorts from different countries

Country	Study population	Number	ESR, mean	ESR, median	Me/Md	Ref.
Denmark 2000	Rheumatoid arthritis	1197			1.30	17 (Table C)
Finland 1980-2004	Rheumatoid arthritis	1892	36	30	1.20	18
USA 1980-2004	Rheumatoid arthritis	478	35	30	1.17	18
Spain 2012-2015	Random population sample	1472	12,50	9,00	1.39	19
Weighted mean of RA populations					1.23	

© 2023 Guski LS et al. *JAMA Network Open.*

eTable E: ***CRP mean and median in cohorts from different countries***

Country	Study population	Number	CRP, mean	CRP, median	Me/Md	Ref.
Finland 1980-2004	Rheumatoid arthritis	1749	25	12	2.08	18
USA 1980-2004	Rheumatoid arthritis	168	18	6	3.0	18
Finland 2005	Young females	1045	2.01	0.81	2.48	20
Finland 2005	Young males	975	1.43	0.59	2.42	20
Europe 2014	Cardiovascular risk	7565	4.25	2	2.13	21
Weighted mean of RA populations					2.16	

5.9 Conversion of median CRP to mean CRP

We used the terms "mean CRP and median CRP and rheumatoid arthritis", which revealed 88 articles in PubMed. We identified 3 references (18,20,21, which reported data on both mean and median CRP from 5 study populations (e**Table E**).

Consequently, we used the weighted mean of 2.16 from these 5 populations as a conversion factor of median CRP to mean CRP (e**Table E**).

5.10 Estimation of number of joints counted

The classical joint evaluation includes 68 joints for TJC and 66 joints for SJC. If the number of evaluated joints was not reported, we assumed this to be 68 for TJC and 66 for SJC.

5.11 Estimation of DAS28

DAS28 is used both as a regression factor in meta regression and as an efficacy measure. As DAS28 is rarely reported directly, we had to estimate DAS28 form other efficacy measures. Therefore, we had to also estimate these efficacy measures in case they were not reported.

5.12 Estimation of missing baseline SJC, TJC, ESR and CRP for calculation of DAS28.

In studies which reported the effect but did not report the baseline values, we estimated the baseline values. For TJC and SJC we calculated the weighted mean of values in the following categories of baseline total joint count: 16-40, 41-59 and 60-68. For TJC these values were 16, 16 and 23. Thus, for TJC we used 16 in the interval 16-59 and 23 in the interval 60-68. For SJC these numbers were 14 and 17.

For ESR and CRP we calculated the weighted mean of all reported baseline values. For ESR this value was 48,6 and for CRP it was 43,0

5.13 DAS28 conversion

Very few studies informed about DAS28 but did not report whether it was based on 3 or 4 items or on CRP or ESR. According to a systematic comparison of composite measures (22) DAS28 values based on 3 or 4 items including ESR or CRP are similar at the 50th centile (i.e., the median) and we therefore recorded the DAS28 values as published in the original articles. A few studies published DAS44 values (based on 44 joints). These were changed to estimated DAS28 values by means of a previously published nomogram (22).

As there were very few studies, which estimated DAS28, we decided to estimate DAS28 based on 3 values from available values of TJC, SJC and ESR or CRP. 20 study arms were estimated from 1 value, 97 study arms from 2 values and 158 study arms from 3 values. As these DAS values were calculated it was not possible to calculate corresponding sd-values. Instead, we used a mean sd of 1.1 estimated by Leil et al on the basis of 130 RCTs, which investigated the effect of 7 different biological DMARDS on DAS28 in 27355 patients with RA (23). We used this sd value for all studies. We divided this sd by sqrt n for each study and thus obtained a se value for each study.

5.14 Estimation of missing SJC values for DAS28

We used TJC values as a direct substitute for missing SJC values (36 studies)

© 2023 Guski LS et al. *JAMA Network Open.*

5.15 Estimation of missing ESR values for DAS28
ESR was available in 116 studies. We calculated the ESR/CRP ratio for each value and then calculated the mean to be 1,37, which was used as a conversion factor of CRP to ESR in 5 studies. If CRP was not available for estimation of a missing ESR, we used the TJC conversion factor (2,56) to estimate ESR in 11 studies.

5.16 Conversion of SJC 29-68 to SJC 28 and of TJC 29-68 to TJC 28
Fuchs and Pincus (24) investigated systematically the individual participant data from three randomized trials, which published SJC based on 28 and 66 joints and found the percentage of SJC28/SJC66 to be 68%, 69%, and 83% (mean value 73%). We chose to use the mean value (73%) as a conversion factor between SJC28 and SJC66 and constructed a nomogram to translate SJC values between 28 and 66 to SJC 28 **(eTable F).**
Similarly, Fuchs and Pincus also investigated TJC and found the percentage of TJC28/TJC66 to be 57%, 60%, and 71% (mean value 63%). We chose to use this mean value (63%) as a conversion factor between TJC28 and TJC66 and constructed a nomogram to translate TJC values between 28 and 66 to TJC 28 **(eTable F).**

eTable F Conversion of SJC 29-68 and TJC 29-68 to SJC28 and TJC 28

Joint Count	SJC 28 conversion factor	TJC 28 conversion factor
22-32	1.0	1.0
33-36	0.97	0.95
37-40	0.94	0.91
41-44	0.91	0.87
45-48	0.88	0.83
49-52	0.85	0.79
53-56	0.82	0.75
57-60	0.79	0.71
61-64	0.76	0.67
65-	0.73	0.63

5.17 Calculation of DAS28 effect
We estimated mean DAS28 values from mean TJC, SJC, ESR and CRP values at baseline and after treatment, but as it was impossible reliably to estimate S.D. values, we could not estimate a DAS28 effect weighted by both S.D. and number of participants. However, we used a recently calculated mean S.D. of 130 studies, which was 1.1 (22), and successively calculated S.E. for each study by dividing with the square root of the number of participants. We then calculated a DAS28 effect, which consequently was weighted by number of participants.

5.18 Estimation of use Glucocorticoids (GC)
Most studies reported whether the use of GC was allowed or not. We recorded this information as a yes/no recording. In studies which did not mention that GC was allowed during the trial, we assumed that GC was not allowed (25-28). Some studies mentioned that patients using GC were excluded. We assumed that GC was not allowed in these studies during the trial (29-34)
We also estimated the dose of GC in the treatment arms, but due to insufficient information in many studies this recording is less reliable.
For all studies, which reported the number of patients receiving GC in each treatment arm, we calculated the mean fraction of patients to receive GC per treatment arm to be 36%. We used this percentage to estimate the number of patients receiving GC in studies reporting GC to be allowed, but not reporting the fraction of patients receiving GC.
For all studies, which reported the doses of GC given to the patients receiving GC, we calculated the mean dose to be 5.64 mg per day per GC treated patient. We used this dose to estimate the dose in studies not reporting the GC dose.
The mean dose per treatment arm including patients not being treated with GC in studies which reported these doses sufficiently was 2,10 mg. The estimated mean dose of all treatment arms based on the premises mentioned above was 2.11 mg.

5.19 Transformation of pl-viscosity to ESR
Viscosity vsl. ESR can be transformed from pl-viscosity by the following formula:
$y = 107{,}08x – 162{,}47$, where y = ESR in mm at one hour and x = PV in centipoise at 25°C (35).

© 2023 Guski LS et al. *JAMA Network Open.*

5.20 Transformation of Lansbury index to TJC and SJC
The Lansbury articular index is an index of total possible inflammation by adding the scores for the severity of inflammation of each joint, weighted for joint surface area. It is based on evaluation of all joints including sternoclavicular joints. We identified two studies (36,37), which measured both TJC, SJC and Lansbury articular index and based on 12 estimations we calculated conversion factors to convert Lansbury articular index to TJC and SJC based on evaluation of 28 joints, as shown in eTable G

eTable G; Conversion factor of Lansbury articular index to TJC and SJC

reference	TJC	Lansbury Art Index(L)	TJC/L	Correction	Final
1	18	64	0.28	0.94	0.26
1	20	71	0.28	0.94	0.26
1	12	34	0.35	0.94	0.33
1	11	29	0.38	0.94	0.36
2	18	91	0.20	1	0.20
2	12	69	0.17	1	0.17
2	19	93	0.20	1	0.20
2	11	63	0.17	1	0.17
2	20	90	0.22	1	0.22
2	10	60	0.17	1	0.17
2	20	87	0.23	1	0.23
2	12	52	0.23	1	0.23
**Mean conversion factor of Lansbury articular index to TJC**					**0,23**

reference	SJC	Lansbury Art Index(L)	TJC/L	Correction	Final
1	14	64	0.22	0.94	0.21
1	14	71	0.20	0.94	0.19
1	9	34	0.26	0.94	0.25
1	7	29	0.24	0.94	0.23
2	12	91	0.13	1	0.13
2	7	69	0.10	1	0.10
2	12	93	0.13	1	0.13
2	7	63	0.11	1	0.11
2	13	90	0.14	1	0.14
2	7	60	0.12	1	0.12
2	14	87	0.16	1	0.16
2	6	52	0.12	1	0.12
**Mean conversion factor of Lansbury articular index to SJC**					**0.16**

eMethods 6. Risk of Bias Within Individual Studies
We assessed the risk of bias at study level by means of the Cochrane risk of bias tool, RoB 2 (38). According to this system, bias is estimated by means of 5 domains: 1, bias arising from the randomization process; 2, bias due to deviations from intended interventions; 3, bias due to missing outcome data; 4, bias in measurement of the outcome; and 5, bias in selection of the reported result. We only evaluated the first 3 domains. We did not evaluate domain 4 because we did our bias assessment on study level instead of outcome level, and domain 5 because most of these older studies were from a time where publication of pre-study plans was not common practice. Each of the domains is classified into three levels ("low risk", "some concern", "high risk"). We

© 2023 Guski LS et al. *JAMA Network Open.*

intended to assign each level with a number, 0 for "low risk", 1 for "some concern" and 2 for "high risk". For each study these numbers were intended to be added to a sum representing the total risk of bias to be used as a potential effect modifier in a meta-regression analysis to explore the effect of bias on the outcome. The bias assessments of all included studies were performed by a single author (LSG). The assessments were controlled by 3 authors (SEA, GJ and NG), who each controlled 1/3 of the bias assessments.

eMethods 7. Planned Method of Analysis

We chose noninformative priors, with a normal distribution with a mean of zero and a precision of 0.0001 for treatment effects, trial baselines, and regression parameters, and a uniform distribution between 0 and 5 for between trial S.D.. We investigated fitting both random and fixed effects models. Models were run in WinBUGS (39) for 20.000 burn-in iterations followed by 30.000 monitored iterations with a thin of 10. Convergence was assessed using traceplots and Brooks-Gelman-Rubin (BGR) plots (40). Model fit was assessed using the deviance information criterion (DIC), the effective number of parameters (pD) and the posterior residual deviance. A more complex model was considered more parsimonious (and therefore selected) if the DIC was 3-5 points lower than a simpler model.

Assessment of Inconsistency: For the assessment of inconsistency, we fitted an unrelated mean effects model (UME) and compared this to the consistency NMA model. For each treatment comparison this allowed comparison between NMA consistency estimates and UME direct estimates, using the posterior median and 95% credible intervals (95%CrI). Disagreements were indicative of potential inconsistency, defined as comparisons in which the 95%CrIs did not overlap. We also plotted a dev-dev plot comparing the residual deviances for each data point in the UME compared to those in the NMA. If the residual deviance in the NMA was 0.5 or more than in the UME (41), we reviewed the study report for an explanation and reasons to exclude the study.

eMethods 8. Effect Modifier Analysis

1.8.1 Effect modifier regression analyses.

When assessing whether a regression parameter was likely to result in meaningful effect modification, we looked at the DIC, the total residual deviance compared to the number of arms, and the pD. We compared these model fit statistics with those from the unadjusted model.

1.8.2 Subgroup analyses.

For each relevant variable, we split the dataset into subgroups, and compared each individual measure of relative treatment effect to the one found in the unadjusted random-effects model.

We performed subgroup analyses of the following categorical variables:

1) Whether or not glucocorticoids were allowed in the study as addition to the study drug.

2) Previous DMARD treatment, with 3 possible categories:

a) treatment-naïve patients
b) patients could have received DMARDS before, but the outcome was not used as inclusion/exclusion criteria
c) included patients were known DMARD non-responders or had stopped the DMARD treatment due to adverse events

3) Analysis type, with two possible categories:

a) intention to treat analysis
b) completer analysis

eResults 1. Study Selection

For the present analysis, we selected 225 reports classified as mono csDMARD (Figure 1: 1098 identified RCTs minus 873 RCTs including other interventions than mono csDMARD treatment) and went through a second level of categorization in which 143 studies were included and 82 excluded. Reasons for exclusion during the search of the 225 identified mono-DMARD studies were the same as during the primary search. In addition, we excluded studies in which relevant outcome data were reported, but presented in a way, which made it impossible to transform them to be integrable in the meta-analysis, and studies in which the treatment groups were randomized to drug tapering or different drug doses without control group. We excluded 82 studies with reasons (Figure 1) and thus included 143 studies by the electronic search. The search in clinicaltrials.gov resulted in 502 references. These dealt with biological drugs, target specific drugs, combination drug treatments, non-DMARD interventions, interventions for comorbidities (diabetes, hypercholesterolemia, gastric ulcer) and analgesics. Some studies were not RCTs, but dose-studies, pharmacokinetic studies, device studies or cohort studies. We found 4 mono-GC studies, one which investigated ultrasound changes and DAS28 (without details on TJC, SJC, ESR or CRP), and 3 slow-release GC studies, which investigated the effect on morning stiffness.

© 2023 Guski LS et al. *JAMA Network Open.*

Additionally, we found two studies investigating Tripterygium Wilfordi Hook, which both were identified by our electronic search (42-43). Accordingly, clinicaltrials.gov did not supply additional studies to be included in the NMA. Additional 26 studies were identified by hand search in reference lists of included studies and meta-analyses. Of these 169 studies (143 + 26), 38 were parallel publications. Thus, we included 131 publications (appendix reference list eR 1-131), which published 133 interventions. One study was included, but the two interventions were not connected to the network (44). The remaining 132 interventions (130 references) were included in the NMA. In our post-hoc screening search we used the term "rheumatoid arthritis" arthritis (6824 hits) filtered by the term "randomized controlled trial" (130 hits). Among the 130 studies we identified 5 drug interventions not involving targeted drugs. Two included non csDMARDs (n-acetyl cysteine and curcumin), one was a combination drug study, one a methotrexate dose-escalation study and one an add-on GC study. Thus, none of these studies were eligible. Consequently, our search is up to date on September 15th, 2022.

eResults 2. Primary Outcome (Complete Analysis) With Methotrexate as Reference, Unadjusted Analysis

The most frequently evaluated outcome was tender joints (118 studies) either as a number (TJC) or as a Ritchie index, which was converted to a number (TJC) as explained in section 4. Thus, to obtain a complete analysis we imputed 14 missing TJC values based on available SJC or ESR values as explained in eMethods 5

In the unadjusted analysis the difference between the FE and RE model was small. Sulfasalazine, mycophenolate, and lobenzarit changed from being outside the credible interval (FE) to be marginally inside (RE) (**eTable 2**). An RE sensitivity analysis of 118 studies (28 treatments) without imputed TJC values showed comparable results to the complete unadjusted analysis regarding the point estimates. The position in the credible interval was unchanged for all drugs (**eTable 3**).

eResults 3. Effect Modifier Analyses

Mean baseline DAS28 with imputed values (n=132) was a significant effect modifier. Also, the model fit parameters improved in the DAS28 model with imputed values. Baseline das28 mean is correlated to the treatment effect. A 1-unit increase in baseline mean DAS28 increases the treatment effect by 2.16 (95%CrI: 0.74, 3.45). The network meta-regression model adjusting for mean baseline DAS28 was therefore our selected model. Network meta-regression models including other covariates did not improve model fit significantly (**eTable 4**).

The subgroup analyses of the categorical variables showed TJCi 95% credible interval overlap in 10 treatment groups with previous inadequate response to a csDMARD, unspecified use of csDMARDs and no use of csDMARDs, and a non-overlapping interval in only one group (placebo) with a previous inadequate response to a csDMARD **(eFigure 1A)**. There was overlap of TJCi CrIs in 12 treatment groups estimated according to statistical method of ITT or completer (**eFigure 1B**). Finally, there was overlap of TJCi CrIs in 11 treatment groups in which GC was allowed or not allowed (**eFigure 1C**).

2.3.1. Risk of bias influence on outcome

Most studies had a high risk of bias. We ran separate NMAs on only 6 studies which had no domains with high risk of bias, but these were not meaningful due to the large lack of interventions. Instead, we assessed the association between bias and estimated effects. We performed a meta-regression analysis where the assigned risk of bias sum was included as a potential effect modifier. This analysis had no impact on the outcome (**eTable 4**)

eResults 4. Primary Outcome With Methotrexate as Reference, Adjusted for Baseline Disease Activity Score 28 (DAS28).

First, we explored potential effect modifiers in meta-regression analyses of TJC outcome (n = 132) versus the potential effect modifier. Some effect modifiers were not reported in all studies. Hence the number of studies and the number of arms was smaller than the complete dataset. In that case, we ran a separate unadjusted model based on only the subset of studies, that reported the regression factor, and compared the model fit statistics between the adjusted and unadjusted model for the same dataset. The following effect modifiers were analyzed one by one: Study year (n=132), study duration (n=132), duration of RA (n=131), mean age (n=126), female% (n=121), RF positivity (n=73), bias estimation (n=132), DAS28 with (n=132) and without imputed values (n=77). None of the effect modifiers without imputations were significantly associated with TJC (**eTable 4**).

eResults 5. Exploration of Inconsistency

Inconsistency was explored by means of an unrelated mean effects model (UME), which was compared to the full model with DAS as a regression factor. An UME model is a model which only uses direct comparisons. If the results from the UME model differ significantly from the NMA model, it suggests there is a problem with inconsistency, that is, the direct and indirect evidence is not in accordance. We looked at both the differences between all treatments, i.e., 406 comparisons (the sum of 28 to1) calculated for both the full model and the

© 2023 Guski LS et al. *JAMA Network Open.*

UME model and a dev-dev plot of the 132 trials. There were no major disagreements between the two models. When comparing the relative treatment effects between individual treatment principles compared in pairs, there was an overlap between the UME and the NMA model in all cases. This suggests they do not differ significantly from each other, hence there is no sign of inconsistency between the direct and indirect model.
eFigure 4 shows the dev-dev plot. This is a comparison of how much each comparison contributes to the total residual deviance. If a certain comparison contributes much more to the total residual deviance in the NMA model compared to the UME model, it suggests inconsistency between the direct and indirect evidence. Trials, where the deviance in the full model was 0.5 or bigger than in the UME model, was checked for any reason for inconsistency. In the present model of 132 trials, the dev-dev plot revealed 11 trials, which had to be checked for inconsistency, but no obvious reason for inconsistency was identified.
Model fit of primary analysis: In the NMA model, the total residual deviance is 276.0 (275 study arms), DIC is 1304.03, between-study S.D. mean is 0.8548 with median 0.8703 and 95%CrI 0.05991 to 1.574. In the UME model, the total residual deviance is 274.5, the DIC is 1327.040, and the between-study S.D. is 0.8066 (median and 95% CrI is 0.7949 (0.06692; 1.622)).

eResults 6. Results of Additional Analyses
Separation of chloroquine and hydroxychloroquine: There were 9 arms for Chloroquine (Cl) and 10 arms for hydroxychloroquine (HCl). There was a large overlap between the credible intervals intervals (CrL) for the two treatments. Neither of the two treatment regimens had CrL that overlapped with placebo (Pl) -3.615 (-5.954;-1.279)for Chloroquine as the reference and -2.367 (-0.9759 ;-3.81) for hydroxychloroquine as the reference. When Chloroquine is used as the reference, the relative effect estimate for hydroxychloroquine is -1.244 (-3.852;1.38).

eReferences

1) The Nordic Cochrane Centre, Higgins JP, Green S, editors (2009) Cochrane handbook for systematic reviews of interventions version 5.0.1, section 7.7.3. The Cochrane Collaboration. Available: www.cochrane-handbook.org.

2) Walter SD, Yao X. Effect sizes can be calculated for studies reporting ranges for outcome variables in systematic reviews. J Clin Epidemiol. 2007; 60:849-52.

3) Sigler JW, Bluhm GB, Duncan H, Sharp JT, Ensign DC, McCrum WR. Gold salts in the treatment of rheumatoid arthritis. A double-blind study. Ann Intern Med 1974;80(1):21–6.

4) Capell HA, Madhok R, Porter DR, et al. Combination therapy with sulfasalazine and methotrexate is more effective than either drug alone in patients with rheumatoid arthritis with a suboptimal response to sulfasalazine: results from the double-blind placebo- controlled MASCOT study. Ann Rheum Dis. 2007; 66:235-41.

5)Weinblatt ME, Kaplan H, Germain BF, Merriman RC, Solomon SD, Wall B, et al. Low-dose methotrexate compared with auranofin in adult rheumatoid arthritis. A thirty-six-week, double-blind trial. Arthritis Rheum 1990;33(3):330–8.

6) Williams HJ, Ward JR, Reading JC, Brooks RH, Clegg DO, Skosey JL, et al. Comparison of auranofin, methotrexate, and the combination of both in the treatment of rheumatoid arthritis. A controlled clinical trial. Arthritis Rheum. 1992;35(3):259–69

7) Rau R, Herborn G, Menninger H, Blechschmidt J. Comparison of intramuscular methotrexate and gold sodium thiomalate in the treatment of early erosive rheumatoid arthritis: 12 month data of a double-blind parallel study of 174 patients. Br J Rheumatol 1997;36(3):345–52.

8) Vischer TL. A double blind multicentre study of OM-8980 and auranofin in rheumatoid arthritis. Ann Rheum Dis 1988;47(7):582–7.

© 2023 Guski LS et al. *JAMA Network Open.*

9) Kim HA, Song YW, HA K, YW S. A comparison between bucillamine and D-penicillamine in the treatment of rheumatoid arthritis. Rheumatol Int 1997;17(1):5–9.

10) Dixey J, Solymossy C, Young A; Early RA Study. Is it possible to predict radiological damage in early rheumatoid arthritis (RA)? A report on the occurrence, progression, and prognostic factors of radiological erosions over the first 3 years in 866 patients from the Early RA Study (ERAS). J Rheumatol Suppl 2004; 69:48-54.

11) Goldbach-Mansky R, Wilson M, Fleischmann R, Olsen N, Silverfield J, Kempf P, et al. Comparison of Tripterygium wilfordii Hook F versus sulfasalazine in the treatment of rheumatoid arthritis: a randomized trial. Ann Intern Med 2009;151(4):229–51.

12) Woodland J, Chaput de Saintonge DM, Evans SJ, Sharman VL, Currey HL. Azathioprine in rheumatoid arthritis: double-blind study of full versus half doses versus placebo. Ann Rheum Dis 1981;40(4):355–9.

13) Zhao L, Jiang Z, Zhang Y, Ma H, Cai C. Analysis of efficacy and safety of treatment of active rheumatoid arthritis with iguratimod and methotrexate. Biomed Res 2017;28(5):2353–9.

14) Capell HA, Hunter JA, Rennie JA, Murdoch RM. Levamisole - a possible alternative to gold and penicillamine in the longterm treatment of rheumatoid arthritis? J Rheumatol 1981;8(5):730–40.

15) Williams HJ, Willkens RF, Samuelson COJ, Alarcon GS, Guttadauria M, Yarboro C, et al. Comparison of low-dose oral pulse methotrexate and placebo in the treatment of rheumatoid arthritis. A controlled clinical trial. Arthritis Rheum 1985;28(7):721–30.

16) Hochberg MC. Auranofin or D-penicillamine in the treatment of rheumatoid arthritis. Ann Intern Med 1986;105(4):528–35.

17) Graudal N, Tarp U, Jurik AG, Galløe AM, Garred P, Milman N, Graudal HK. Inflammatory patterns in rheumatoid arthritis estimated by the number of swollen and tender joints, the erythrocyte sedimentation rate, and hemoglobin: longterm course and association to radiographic progression. J Rheumatol 2000; 27:47-57.

18) Sokka T, Pincus T. Erythrocyte sedimentation rate, C-reactive protein, or rheumatoid factor are normal at presentation in 35%-45% of patients with rheumatoid arthritis seen between 1980 and 2004: analyses from Finland and the United States. J Rheumatol 2009; 36:1387-90.

19) Alende-Castro V, Alonso-Sampedro M, Vazquez-Temprano N, et al. Factors influencing erythrocyte sedimentation rate in adults: new evidence for an old test. Medicine (Baltimore). 2019;98: e16816.

20) Raitakari M, Mansikkaniemi K, Marniemi J, Viikari JS, Raitakari OT. Distribution and determinants of serum high-sensitive C-reactive protein in a population of young adults: The Cardiovascular Risk in Young Finns Study. J Intern Med 2005; 258:428-34.

21) Halcox JP, Roy C, Tubach F et al. C-reactive protein levels in patients at cardiovascular risk: EURIKA study. BMC Cardiovasc Disord 2014;14: 25.

22) Ranganath VK, Yoon J, Khanna D et al. Comparison of composite measures of disease activity in an early seropositive rheumatoid arthritis cohort. Ann. Rheum. Dis. 2007; 66:1633–1640

23) Leil TA, Lu Y, Bouillon-Pichault M, Wong R, Nowak M. Model-Based Meta-Analysis Compares DAS28 Rheumatoid Arthritis Treatment Effects and Suggests an Expedited Trial Design for Early Clinical Development. Clin Pharmacol Ther 2021; 109:517-527.

24) Fuchs H.A., Pincus T. Reduced joint counts in controlled clinical trials in rheumatoid arthritis. Arthritis Rheum 1994; 37:470–475.

25) Mbuyi-Muamba JM, Dequeker J. A comparative trial of timegadine and D-penicillamine in rheumatoid

© 2023 Guski LS et al. *JAMA Network Open.*

arthritis. Clin Rheumatol 1983;2(4):369–74.

26) Clemens LE, Ansell BM, Bernstein RM, Hall MA, McCauley D. A comparison of low dose levamisole and penicillamine in rheumatoid arthritis. Aust N Z J Med 1983;13(6):578–82.

27) Reilly PA, Burns A, Moran CJ. Sulphasalazine in early rheumatoid arthritis. Clin Exp Rheumatol . 1991;9(1):90–1.

28) Landewe RB, Goei The HS, van Rijthoven AW, Breedveld FC, Dijkmans BA. A randomized, double-blind, 24-week controlled study of low-dose cyclosporine versus chloroquine for early rheumatoid arthritis. Arthritis Rheum 1994;37(5):637–43.

29) Woodland J, Chaput de Saintonge DM, Evans SJ, Sharman VL, Currey HL. Azathioprine in rheumatoid arthritis: double-blind study of full versus half doses versus placebo. Ann Rheum Dis 1981;40(4):355–9.

30) Manthorpe R, Horbov S, Sylvest J, Vinterberg H. Auranofin versus penicillamine in rheumatoid arthritis. One-year results from a prospective clinical investigation. Scand J Rheumatol 1986;15(1):13–22.

31)Capell HA, Marabani M, Madhok R, Torley H, Hunter JA. Degree and extent of response to sulphasalazine or penicillamine therapy for rheumatoid arthritis: results from a routine clinical environment over a two-year period. Q J Med 1990;75(276):335–44.

32) Scott DL, Greenwood A, Davies J, Maddison PJ, Maddison MC, Hall ND. Radiological progression in rheumatoid arthritis: do D-penicillamine and hydroxychloroquine have different effects?. Br J Rheumatol . 1990;29(2):126–7.

33) Clark P, Casas E, Tugwell P, Medina C, Gheno C, Tenorio G, et al. Hydroxychloroquine compared with placebo in rheumatoid arthritis. A randomized controlled trial. Ann Intern Med 1993;119(11):1067–71.

34) Dougados M, Combe B, Cantagrel A, Goupille P, Olive P, Schattenkirchner M, et al. Combination therapy in early rheumatoid arthritis: a randomised, controlled, double blind 52 week clinical trial of sulphasalazine and methotrexate compared with the single components. Ann Rheum Dis 1999;58(4):220–5.

35 Hutchinson RM, Eastham RD. A comparison of the erythrocyte sedimentation rate and plasma viscosity in detecting changes in plasma proteins. J Clin Pathol.1977;30:345-9.

36) Rau R, Herborn G, Karger T, Menninger H, Elhardt D, Schmitt J. A double-blind comparison of parenteral methotrexate and parenteral gold in the treatment of early erosive rheumatoid arthritis: an interim report on 102 patients after 12 months. Semin Arthritis Rheum. 1991 Oct;21(2 Suppl 1):13-20.

37) Raeman F, Hanssens F, Delattre M. An European open multicentre trial with auranofin in rheumatoid arthritis. Clin Rheumatol. 1984 Mar;3 Suppl 1:33-8

38)Sterne JAC, Savović J, Page MJ, et al. RoB 2: a revised tool for assessing risk of bias in randomised trials. *BMJ* 2019;366:l4898.

39) Lunn DJ, Thomas A, Best N, Spiegelhalter D. WinBUGS — a Bayesian modelling framework: concepts, structure, and extensibility. *Statistics and Computing* 2000; 10(4): 325–337.

40) Brooks SP, Gelman A. General methods for monitoring convergence of iterative simulations. Journal of Computational and Graphical Statistics 1998; 7(4): 434–455

41) Daly C, Downing BC, Welton NJ: A Practical Guide to Inconsistency Checks in Bayesian Network Meta-Analysis. NICE guidelines technical support unit 2021. https://www.bristol.ac.uk/population-health-bristol.ac.uk/population-health-sciences/centres/cresyda/mpes/nice/tsu-reports/

© 2023 Guski LS et al. *JAMA Network Open.*

42) Goldbach-Mansky R, Wilson M, Fleischmann R, Olsen N, Silverfield J, Kempf P, et al. Comparison of Tripterygium wilfordii Hook F versus sulfasalazine in the treatment of rheumatoid arthritis: a randomized trial. Ann Intern Med 2009;151(4):229–51

43) Lv QW, Zhang W, Shi Q, WJ Z, Li X, Chen H, et al. Comparison of Tripterygium wilfordii Hook F with methotrexate in the treatment of active rheumatoid arthritis (TRIFRA): a randomised, controlled clinical trial. Ann Rheum Dis 2015;74(6):1078–86.

44) Taggart AJ, Neumann VC, Hill J, Astbury C, Le Gallez P, Dixon JS. 5-Aminosalicylic acid or sulphapyridine. Which is the active moiety of sulphasalazine in rheumatoid arthritis? Drugs 1986;32 Suppl 1:27–34.

© 2023 Guski LS et al. *JAMA Network Open.*

4. eTables and eFigures

eTable 1. Baseline Characteristics, Primary Outcome and Bias Score of Included Studies

Ref No	TestDrugs	N patients	Base DAS28	Analysis type*	Base TJC	Outcome TJCi
51	Gc/Ns	44/42	6.18/5.91	C	19.8/17	7.30 [1.80, 12.80]/5.80 [0.93, 10.67]
52	Cl/Pl	34/32	6.44/6.02	C	23.5/19	13.40 [5.51, 21.29]/2.80 [-5.33, 10.93]
53	Gc/Ns	41/36	6.19/5.89	C	16/14	9.00 [6.04, 11.96]/3.50 [0.17, 6.83]
54	Cl/Pl	42/40	5.9/6.01	C	14/14	3.94 [0.90, 6.98]/1.09 [-2.61, 4.79]
55	Au/Pl	90/95	6.53/6.65	C	17.3/19.2	9.50 [7.27, 11.73]/6.60 [3.96, 9.24]
56	Cl/Pl	19/22	6.21/6.66	C	13.7/16.4	2.60 [-1.60, 6.80]/1.90 [-1.05, 4.85]
57	Cl/Pl	53/60	6.56/6.56	C	23/23	7.00 [2.37, 11.63]/3.00 [-1.94, 7.94]
58	Cp/CpL	20/28	6.86/6.77	C	28/28	21.00 [15.11, 26.89]/7.00 [0.67, 13.33]
59	Dp/Pl	30/38	6.73/6.68	C	19/19	7.10 [0.76, 13.44]/4.00 [-0.05, 8.05]
60	Cp/Pl	10/11	6.62/6.53	C	19.2/18.5	6.20 [-2.91, 15.31]/4.00 [-5.18, 13.18]
61	Au/Pl	22/21	7.02/6.8	C	26/25	13.00 [5.89, 20.11]/7.00 [-1.34, 15.34]
62	Au/Pl	13/14	5.52/5.98	C	11.6/14.2	3.80 [-0.94, 8.54]/2.30 [-4.07, 8.67]
63	Au/Dp	40/46	6.26/6.42	ITT	15.7/17.2	6.90 [3.61, 10.19]/7.70 [4.36, 11.04]
64	Dp/Pl	27/39	6.73/6.63	C	19.9/19.1	7.20 [2.12, 12.28]/1.30 [-3.58, 6.18]
65	Dp/Az	29/29	5.63/5.78	C	9.4/10.6	1.70 [-1.10, 4.50]/1.30 [-1.99, 4.59]
66	Cp/Pl	11/11	7.05/6.4	C	29.5/22	13.00 [7.25, 18.75]/0.00 [-7.10, 7.10]
67	Dp/Pl	5/7	5.99/5.48	C	13.8/10.9	5.00 [-11.43, 21.43]/0.60 [-18.43, 19.63]
68	Au/Pl	17/17	5.59/5.17	C	12.4/11.6	1.50 [-2.40, 5.40]/0.80 [-3.74, 5.34]
69	Lm/Pl	13/11	6,35/5,92	C	16.3/13.5	7.20 [0.67, 13.73]/-2.70 [-9.80, 4.40]
70	Af/Cl/Az	10/10/9	7.23/6.80/7.01	C	30/27/29	15.00 [0.10, 29.90]/13.00 [0.03, 25.97]/. 18.00 [0.45, 35.55]
71	Au/Lm	9/6	6.08/6.47	C	16.6/18.7	6.60 [-2.53, 15.73]/8.60 [-1.44, 18.64]
72	Dp/Pl(Ns)	21/16	5.73/6.06	C	10.3/12.9	1.40 [-2.35, 5.15]/-0.30 [-6.28, 5.68]
73	Au/Dp	35/44	5.66/6.05	C	14.6/16.5	5.90 [2.07, 9.73]/6.20 [2.69, 9.71]
74	Lm/Pl	102/101	6.12/6.44	C	13/14.5	3.69 [2.59, 4.79]/0.99 [-0.29, 2.27]
75	Au/Dp	16/10	5.63/6.49	ITT	13/21	3.00 [-2.02, 8.02]/11.00 [2.72, 19.28]
76	Lm/Pl	10/10	6.76/6.34	ITT	19.5/20	3.50 [-2.20, 9.20]/0.50 [-4.37, 5.37]
77	Az/Pl	8/11	5.71/5.79	C	7.23/6.85	4.32 [1.69, 6.95]/2.03 [0.48, 3.58]
78	Au/Dp/Lm	16/18/9	7.02/7.11/6.9	C	21/23.8/21.7	9.20 [7.66, 10.74]/6.40 [5.19, 7.61]/ 12.60 [6.22, 18.98]
78	Au/Lm	15/20	7.17/6.61	C	24.1/21	8.30 [6.56, 10.04]/2.90 [1.44, 4.36]
79	Dp/Lm	17/14	5.61/5.79	C	7.3/5.3	3.00 [-0.03, 6.03]/3.00 [-0.85, 6.85]
80	Au/Af	18/25	7.44/7.94	C	21/26	10.00 [2.34, 17.66]/14.30 [9.20, 19.40]
81	Au/Dp/Af	5/8/11	5.55/5.51/5.55	C	9.7/11.5/11.8	5.20 [-1.00, 11.40]/6.40 [0.72, 12.08]/ 2.90 [-2.53, 8.33]
82	Au/Af/Pl	23/26/17	5.70/5.67/6.10	C	10.7/10/14	9.60 [6.36, 12.84]/5.90 [3.07, 8.73]/ 1.40 [-3.78, 6.58]
83	Lm/Pl	111/50	5.96/6.18	C	11/12	5.50 [4.50, 6.50]/1.90 [0.31, 3.49]
84	Dp/Tp	25/25	6.06/5.95	C	15.1/13.5	7.20 [1.44, 12.96]/6.40 [3.67, 9.13]
85	Dp/Af	16/17	6.14/5.96	C	15.3/14.7	-0.70 [-6.24, 4.84]/6.00 [1.09, 10.91]
86	Dp/Tg	20/19	7.08/7.03	C	32/32	8.40 [-2.60, 19.40]/5.85 [-3.46, 15.16]

© 2023 Guski LS et al. *JAMA Network Open.*

87	Dp/Lm	10/8	6.17/5.99	C	15.3/15.3	11.00 [5.66, 16.34]/13.60 [8.01, 19.19]
88	Au/Ns	8/12	7.26/7.06	C	26.5/24.7	16.40 [5.43, 27.37]/10.40 [0.83, 19.97]
Ref No	TestDrug	N patients	Base DAS28	Analysis type	Base TJC	Outcome TJCi
89	Af/Pl	130/143	6.71/6.53	C	23.4/22.4	8.10 [5.19, 11.01]/5.10 [2.20, 8.00]
90	Au/Ns	16/17	5.74/6.02	C	14/14.7	1.50 [-1.74, 4.74]/3.50 [-0.40, 7.40]
91	Dp/Pl	61/40	7.36/7.55	C	34/36	13.00 [8.66, 17.34]/7.00 [1.67, 12.33]
92	Au/Af/Pl	54/64/43	6.94/7.08/6.84	C	30/32/29	11.00 [4.95, 17.05]/9.00 [3.46, 14.54]/ -1.00 [-7.56, 5.56]
93	Su/Dp	23/22	6.56/6.37	C	18.7/16.5	8.10 [3.47, 12.73]/4.80 [0.23, 9.37]
94	Lz/Pl	86/90	5.55/5.48	C	8.4/8	2.49 [0.60, 4.38]/0.87 [-1.26, 3.00]
95	Cl/Ds	24/21	5.41/5.71	C	7.9/10.2	2.40 [0.04, 4.76]/3.80 [0.70, 6.90]
96	Dp/Az/Lm	15/20/13	6.62/6.98/6.83	C	23.5/29/25	6.30 [0.96, 11.64]/8.30 [2.86, 13.74]/ 7.20 [-0.74, 15.14]
97	Dp/Az	64/70	7.05/7.09	C	32/32	11.00 [5.50, 16.50]/10.00 [4.97, 15.03]
98	Au/Af	52/56	6.28/6.37	C	22.2/23.6	6.50 [1.96, 11.04]/4.30 [-0.75, 9.35]
99	Mt/Pl	57/53	7.2/7.3	C	30/32	11.00 [5.45, 16.55]/3.00 [-2.45, 8.45]
100	Mt/Pl	15/16	7.87/7.36	C	37/36	26.00 [17.82, 34.18]/4.00 [-3.87, 11.87]
101	Mt/Pl	12/12	6.86/6.86	C	26.2/26.2	13.60 [1.07, 26.13]/0.00 [-13.60, 13.60]
102	Dp/Af	28/26	6.13/6.32	C	19.2/19.4	13.00 [8.00, 18.00]/9.10 [3.20, 15.00]
103	Af/Pl	154/149	6.53/6.61	ITT	25/26	7.30 [5.27, 9.33]/4.50 [2.35, 6.65]
104	Dp/Af	19/20	6.53/6.3	ITT	22/28	5.00 [-2.82, 12.82]/3.00 [-7.58, 13.58]
105	Cs/Pl	9/13	6.22/5.74	C	15.7/14.2	8.30 [3.95, 12.65]/2.00 [-1.71, 5.71]
106	Su/Pl	31/31	7.97/7.64	C	34/32	14.00 [7.73, 20.27]/8.00 [1.45, 14.55]
107	Mt/Az	18/19	6.46/6.47	C	22.2/21.6	13.20 [6.02, 20.38]/8.00 [1.45, 14.55]
108	Cl/Dp	18/26	4.96/5.09	ITT	5.3/4.7	2.00 [-1.85, 5.85]/1.40 [-1.81, 4.61]
109	Su/Au/Pl	47/30/32	7.11/7.26/7.44	C	28/30/34	20.00 [15.34, 24.66]/18.00 [12.14, 23.86]/ 15.00 [7.15, 22.85]
110	Af/Om	61/69	6.04/6.08	C	18.2/18.5	4.40 [1.56, 7.24]/6.50 [4.16, 8.84]
111	Mt/Au	18/13	5.89/6	C	20.3/19.3	8.00 [1.47, 14.53]/10.40 [2.71, 18.09
112	Au/Af	40/49	5.84/6.05	C	19.7/20.9	8.40 [4.83, 11.97]/7.10 [3.11, 11.09]
113	Cs/CsL	12/13	7.18/7.25	C	33.6/34	18.80 [12.37, 25.23]/2.80 [-5.17, 10.77]
114	Cs/Pl	22/24	6/5.9	C	12/13.3	1.00 [-2.68, 4.68]/-1.00 [-4.93, 2.93]
115	Af/Pl	64/65	4.78/4.18	ITT	11/8	5.10 [2.42, 7.78]/-1.10 [-4.10, 1.90]
116	Su/Cl	30/30	6.09/6.14	ITT	15/16	4.00 [-0.58, 8.58]/4.00 [-0.08, 8.08]
117	Mt/Au	15/12	5.93/6.05	C	15.3/17.6	3.10 [-4.41, 10.61]/2.70 [-3.90, 9.30]
118	Su/Dp	28/26	6.12/6.34	ITT	16.9/19.1	6.00 [-0.09, 12.09]/5.70 [0.33, 11.07]
119	Mt/Az	18/19	6.8/6.94	C	20.5/21.6	7.80 [-0.14, 15.74]/5.90 [-0.25, 12.05]
120	Su/Dp	61/54	7.93/7.49	C	30.7/26.4	16.31 [12.62, 20.00]/15.35 [11.48, 19.22]
121	Cs/Pl	72/72	6.26/6.16	ITT	20.7/19	6.00 [3.85, 8.15]/0.30 [-2.36, 2.96]
122	Mt/Af	133/137	6.6/6.33	ITT	24.5/22.5	14.40 [12.37, 16.43]/7.67 [5.38, 9.96]
123	Dp/Om	17/17	6.57/6.53	C	19.6/19.1	10.70 [6.80, 14.60]/7.60 [3.66, 11.54]
124	Cl/Dp	17/17	5.91/5.94	C	9.3/9.7	5.30 [2.57, 8.03]/4.40 [1.32, 7.48]
125	Cs/Dp	46/46	5.70/5.66	ITT	13.1/13	2.90 [0.41, 5.39]/-2.70 [-9.80, 4.40]
126	Cs/Az	18/15	5.73/5.67	C	17/16	5.00 [2.31, 7.69]/3.00 [0.27, 5.73]
127	Mt/Az	30/20	6.94/7	C	22.5/23	10.40 [6.30, 14.50]/3.70 [-2.42, 9.82]

© 2023 Guski LS et al. *JAMA Network Open.*

128	Su/Pl	19/10	5.14/4.93	C	8.7/9.1	4.40 [1.88, 6.92]/-0.60 [-6.08, 4.88]
129	Mt/Au	26/21	6.42/6.69	C	17.8/19.7	6.90 [2.84, 10.96]/7.60 [3.78, 11.42]
Ref No	TestDrug	N patients	Base DAS28	Analysis type	Base TJC	Outcome TJCi
130	Mt/Af	75/69	7.03/7.12	C	29/31	14.00 [9.75, 18.25]/15.00 [10.50, 19.50]
131	Mt/Az	67/72	7.16/7.09	ITT	24/24	7.00 [2.01, 11.99]/1.00 [-10.20, 12.20]
132	Mt/Ap	12711	7,88/8.79	C	35.9/48.2	25.30 [12.63, 37.97]/30.80 [9.93, 51.67]
133	Su/Af	82/79	5.41/5.7	C	7.3/9.3	2.00 [-0.91, 4.91]/1.30 [-1.70, 4.30]
134	Su/Pl	29/36	5,02/5.08	C	8.3/9.5	3.40 [0.28, 6.52]/4.60 [2.10, 7.10]
135	Af/Py	142/139	5.69/5.84	C	12.5/12.7	4.90 [2.67, 7.13]/5.50 [2.98, 8.02]
136	Su/Cl	17/23	5,7/5.85	C	8/9	3.00 [-0.15, 6.15]/2.00 [-1.22, 5.22]
137	Cl/Pl	65/61	5.15/5.05	ITT	8.6/7.6	6.15 [4.97, 7.33]/3.90 [2.68, 5.12]
138	Su/Pl	36/40	4.33/4.03	ITT	7.1/6.1	2.10 [0.70, 3.50]/0.40 [-0.79, 1.59]
139	Cl/Ds	24/23	5.42/5.04	C	8.2/6.3	1.20 [-3.29, 5.69]/1.60 [-3.09, 6.29]
140	Cs/Cl	19/20	6.42/6.51	C	14/14	5.00 [2.12, 7.88]/4.00 [-0.04, 8.04]
141	Cs/Az	47/46	6.68/6.54	C	19.9/18.6	5.40 [3.28, 7.52]/5.10 [2.71, 7.49]
142	Mt/Pd	14/23	6.41/6.36	C	21.8/21.9	17.30 [11.53, 23.07]/17.80 [12.96, 22.64]
143	Mt/Az	18/20	6.40/6.32	ITT	16.7/15.3	4.70 [0.98, 8.42]/5.30 [1.64, 8.96]
144	Cl/Pl	124/116	6.66/6.74	ITT	27.85/29.14	11.07 [9.06, 13.08]/9.16 [7.02, 11.30]
145	Cl/Af	35/30	6.54/6.82	C	20.2/22.7	15.40 [11.20, 19.60]/13.80 [7.71, 19.89]
146	Su/Pl	16/16	5.5/4.66	ITT	8/5	4.00 [2.19, 5.81]/-0.50 [-2.98, 1.98]
147	Cl/Pl	59/60	6.61/6.62	ITT	25.3/26.2	2.30 [-2.83, 7.43]/4.20 [-0.88, 9.28]
148	Cl/Gc	28/28	5.89/6.01	ITT	11.3/11.3	5.70 [2.18, 9.22]/4.10 [0.56, 7.64]
149	Le/Pl	103/101	7.2/7.28	ITT	35/37	16.50 [13.22, 19.78]/9.70 [6.06, 13.34]
150	Mt/Su/Cl	29/18/20	6.06/6.08/6.13	C	12.8/13.2/13.9	7.10 [5.06, 9.14]/6.20 [2.90, 9.50]/ 5.60 [2.28, 8.92]
151	Dp/Bc	22/24	7.07/7.24	ITT	25.8/29.1	3.54 [-5.69, 12.77]/4.82 [-3.15, 12.79]
152	Mt/Su	35/34	6,79/6.71	ITT	20.6/20.8	9.30 [6.24, 12.36]/9.00 [5.68, 12.32]
153	Mt/Au	73/53	6,58/6.59	C	18.4/20.3	6.50 [3.45, 9.55]/7.90 [4.40, 11.40]
154	Cs/Au	104/87	5.81/5.7	C	12.3/12.1	5.60 [4.42, 6.78]/5.70 [4.08, 7.32]
155	Af/Rc	30/30	5.64/5.68	ITT	9.3/9.7	3.60 [1.26, 5.94]/4.30 [1.96, 6.64]
156	Mt/Su	69/68	5.68/5.71	ITT	11/11.7	2.80 [0.79, 4.81]/4.70 [2.77, 6.63]
157	Su/Le/Pl	132/130/91	6.78/7.04/6.8	ITT	16.7/18.8/16.3	8.10 [6.89, 9.31]/9.70 [8.44, 10.96]/ 4.30 [2.69, 5.91]
158	Mt/Le/Pl	180/182/118	6.32/6.42/6.52	ITT	15.8/15.5/16.5	6.60 [5.43, 7.77]/7.70 [6.66, 8.74]/ 3.00 [1.53, 4.47]
159	Cs/Pl	93/61	6.29/6.02	ITT	22.3/19.5	6.50 [3.12, 9.88]/0.60 [-3.48, 4.68]
160	Mt/Rc	50/50	5.86/5.68	ITT	9.3/8.1	5.40 [3.82, 6.98]/3.90 [2.43, 5.37]
161	Mt/Le	498/501	6.93/6.85	ITT	17.7/17,2	9.70 [9.05, 10.35]/8.20 [7.53, 8.87]
162	Mt/Le	8/7	7.04/7.39	C	17/18	5.00 [-4.47, 14.47]/12.00 [5.22, 18.78]
163	Mt/Le	19/16	7.18/7.34	C	19/21	7.00 [1.80, 12.20]/11.00 [5.07, 16.93]
164	Mt/Cs	50/50	6.35/6	ITT	26/22	7.10 [2.27, 11.93]/7.40 [2.71, 12.09]
165	Mt/Au	69/72	5.33/5.31	ITTR	7.3/8	2.60 [1.03, 4.17]/4.00 [2.28, 5.72]
166	Tl/Pl	64/71	6.8/6.82	ITT	28.4/28.5	12.90 [9.44, 16.36]/1.00 [-3.14, 5.14]
167	Gc/Pl	40/41	4.83/5.32	ITTR	8.9/8.6	2.00 [-0.24, 4.24]/0.00 [-2.16, 2.16]
168	Su/Ns	59/54	5.68/6.04	ITTR	13.1/15.8	0.80 [-1.29, 2.89]/1.00 [-1.19, 3.19]

© 2023 Guski LS et al. *JAMA Network Open.*

169	Tl/Pl	153/157	6.55/6.5	ITT	26.6/26.3	7.25 [4.57, 9.93]/1.86 [-1.02, 4.74]
170	Mt/Le	213/291	5.65/5.78	C	13/13.6	5.96 [5.09, 6.83]/6.73 [5.93, 7.53]
Ref No	TestDrug	N patients	Base DAS28	Analysis type	Base TJC	Outcome TJCi
171	Tl/Pl	58/64	6.23/6.19	ITT	13.7/11.8	5.30 [2.52, 8.08]/0.40 [-1.58, 2.38]
172	Cl/Em/Pl	17/18/16	6.56/6.54/6.59	C	16.2/15.4/16.2	2.80 [-8.72, 14.32]/3.10 [-7.14, 13.34]/ 1.30 [-8.93, 11.53]
173	Ig/Pl	93/95	6.17/6.61	ITT	14/17	6.00 [3.66, 8.34]/2.00 [-0.68, 4.68]
174	Mt/Mx	187/188	6.66/6.51	ITT	24/23	18.00 [15.93, 20.07]/19.50 [17.46, 21.54]
175	Su/Tw	32/44	7.1/7.17	C	33/34	20.20 [12.08, 28.32]/18.70 [12.01, 25.39]
176	Mt/Ig	163/163	6.26/6.25	ITT	14.8/14.3	7.40 [6.15, 8.65]/6.90 [5.71, 8.09]
177	Mp/Pl	147/69	6.77/6.79	ITT	18.4/18.1	5.26 [3.01, 7.51]/2.23 [-1.41, 5.87]
177	Mp/Pl	139/69	6.83/6.83	ITT	19/18.7	2.85 [0.21, 5.49]/2.80 [-0.89, 6.49]
178	Mt/Le	89/91	6.97/6.84	C	18/17	10.00 [8.48, 11.52]/8.00 [6.42, 9.58]
179	Mt/Tw	69/69	6.52/6.19	ITT	16.2/13.8	9.10 [6.44, 11.76]/8.90 [6.61, 11.19]
180	Mt/Ig	33/34	7.34/7.25	C	18.9/17.3	14.88 [12.49, 17.27]/11.97 [9.97, 13.97]
181	Sp/As	15/15	6.54/5.97	C	17.3/14	12.00 [7.73, 16.27]/6.70 [1.55, 11.85]

*Analysis type: C: Completer; ITT: Intention to treat; ITTR: Intention to treat, rescue allowed

eTable 2. Unadjusted Tender Joint Count Imputed (TJCi) Analysis Using Fixed- and Random-Effects Models

	Fixed effect model			Random effect model		
	median	0,025	0,975	median	0,025	0,975
Oral Methotrexate (Mt) (1)	Ref	Ref	Ref	Ref	Ref	Ref
Sulfasalazine (Su)(2)	-1,64	-2,65	-0,64	-1,40	-2,78	0,02
Leflunomide (Le)(3)	-0,33	-0,92	0,27	-0,01	-1,22	1,29
Cyclosporine (Cs)(4)	-0,44	-1,83	0,95	-0,45	-2,23	1,34
Injectable gold (Au) (5)	0,08	-1,01	1,17	-0,30	-1,79	1,11
Chloroquine (Cl)(6)	-2,26	-3,53	-0,98	-2,11	-3,77	-0,43
D-penicillamine (Dp) (7)	-1,90	-3,18	-0,60	-1,78	-3,40	-0,16
Azathioprin (Az)(8)	-2,64	-4,10	-1,20	-2,56	-4,34	-0,76
Oral gold (Af)(9)	-2,36	-3,64	-1,07	-2,24	-3,82	-0,66
CycloPhosphamide (Cp)(10)	6,40	0,92	11,86	6,28	0,54	11,96
Levamisole (Lm)(11)	-2,21	-3,50	-0,92	-2,00	-3,85	-0,07
Tacrolimus (Tl)(12)	1,50	-0,95	3,96	1,67	-1,32	4,67
Tripterygium wilfordii (Tw)(13)	-0,64	-4,73	3,43	-0,67	-5,29	3,89
Iguratimod (Ig)(14)	-1,04	-2,43	0,34	-1,26	-3,39	0,83
Timegadine (Tg)(15)	-4,42	-18,26	9,50	-4,29	-18,47	9,91
Bucillamin (Bc)(16)	-0,63	-12,50	11,32	-0,54	-12,71	11,61
Tiopronin (Tp)(17)	-2,70	-9,02	3,63	-2,57	-9,48	4,34
Pyritinol (Py)(18)	-1,75	-5,31	1,85	-1,65	-6,13	2,82
Mycophenolate (Mc)(19)	-3,24	-6,46	-0,03	-3,30	-7,05	0,44
OM89801 (Om)(20)	-1,76	-4,96	1,43	-1,83	-5,63	1,95
Lobenzarit (Lz)(21)	-3,25	-6,18	-0,28	-3,29	-7,25	0,65
Rheumacon (Rc)(22)	-1,55	-3,37	0,26	-1,51	-4,14	1,11
Prospidin (Pd)(23)	0,53	-6,68	7,79	0,53	-7,12	8,18
Aminopterin (Ap)(24)	5,53	-17,27	28,31	5,59	-17,19	28,57
Eazmow plus (Em)(25)	-2,53	-14,80	9,78	-2,52	-15,09	9,98
Dapsone (Ds)(26)	-1,11	-4,62	2,38	-1,02	-5,10	3,09
Glucocorti-coide (Gc) (27)	-2,27	-4,56	0,02	-2,24	-5,06	0,55
Incectable methotrexate (Mx) (28)	1,19	-1,57	3,96	0,98	-2,59	4,51
Placebo (Pl)(29)	-4,87	-5,74	-4,00	-4,90	-6,12	-3,72
totresdev	313,80			270,80		
total number arms	275,00			275,00		
pd	159,90			185,11		
DIC	1324,30			1306,46		

eTable 3. Tender Joint Count Imputed (TJCi) Sensitivity Analysis Comparing Analyses With and Without Imputated Values

	A (n=132)			B (n=118)		
	median	0.025	0.975	median	0.025	0.975
Oral Methotrexate (Mt) (1)	ref			ref		
Sulfasalazine (Su)(2)	-1,40	-2,78	0,02	-1,40	-2,82	0,05
Leflunomide (Le)(3)	-0,01	-1,22	1,29	0,02	-1,21	1,33
Cyclosporine (Cs)(4)	-0,45	-2,23	1,34	-0,43	-2,23	1,38
Injectable gold (Au) (5)	-0,30	-1,79	1,11	-0,29	-1,81	1,13
Chloroquine (Cl)(6)	-2,11	-3,77	-0,43	-1,98	-3,77	-0,18
D-penicillamine (Dp) (7)	-1,78	-3,40	-0,16	-1,84	-3,50	-0,16
Azathioprin (Az)(8)	-2,56	-4,34	-0,76	-2,56	-4,43	-0,70
Oral gold (Af)(9)	-2,24	-3,82	-0,66	-2,53	-4,16	-0,90
CycloPhosphamide (Cp)(10)	6,28	0,54	11,96	8,69	2,27	15,04
Levamisole (Lm)(11)	-2,00	-3,85	-0,07	-1,95	-3,82	-0,01
Tacrolimus (Tl)(12)	1,67	-1,32	4,67	1,74	-1,24	4,76
Tripterygium wilfordii (Tw)(13)	-0,67	-5,29	3,89	-0,69	-5,34	3,90
Iguratimod (Ig)(14)	-1,26	-3,39	0,83	-1,24	-3,41	0,86
Timegadine (Tg)(15)	-4,29	-18,47	9,91	-4,31	-18,52	9,91
Bucillamin (Bc)(16)	-0,54	-12,71	11,61	-0,47	-12,56	11,58
Tiopronin (Tp)(17)	-2,57	-9,48	4,34	-2,65	-9,52	4,20
Pyritinol (Py)(18)	-1,65	-6,13	2,82	-1,93	-6,42	2,57
Mycophenolate (Mc)(19)	-3,30	-7,05	0,44	-3,21	-7,01	0,59
OM89801 (Om)(20)	-1,83	-5,63	1,95	-2,05	-5,85	1,72
Lobenzarit (Lz)(21)	-3,29	-7,25	0,65			
Rheumacon (Rc)(22)	-1,51	-4,14	1,11	-1,63	-4,28	1,03
Prospidin (Pd)(23)	0,53	-7,12	8,18	0,48	-7,21	8,13
Aminopterin (Ap)(24)	5,59	-17,19	28,57	5,49	-17,54	28,18
Eazmow plus (Em)(25)	-2,52	-15,09	9,98	-2,44	-15,15	10,09
Dapsone (Ds)(26)	-1,02	-5,10	3,09	-0,87	-5,08	3,30
Glucocorti-coide (Gc) (27)	-2,24	-5,06	0,55	-3,08	-6,56	0,33
Incectable methotrexate (Mx) (28)	0,98	-2,59	4,51	0,98	-2,64	4,49
Placebo (Pl)(29)	-4,90	-6,12	-3,72	-4,83	-6,07	-3,62

eTable 4. Model Fit Parameters for Tender Joint Count Imputed (TJCi) Analyses Including and Excluding Effect Modifiers in Metaregression Analyses

					A				B			
**Effect modifier**	**median**	**0.25**	**97.5**	**Study arms**	**DIC**	**pD**	**totresdev**	**sd**	**DIC**	**pD**	**totresdev**	**sd**
Age	-0.029	-0.25	0.2106	263	1235.8	177.2	260.3	1.25	1234.6	176.5	259.9	1.24
Gender	-0.033	-0.163	0.0915	253	1182.7	172.5	245.5	1.27	1182.0	171.0	246.3	1.23
Duration RA	-0.02	-0.045	0.0054	273	1295.2	186.2	265.4	1.28	1296.5	184.0	268.9	1.22
Studydur.	-0.012	-0.068	0.0463	275	1307.5	185.9	270.9	1.23	1306.5	185.1	270.8	1.21
Bias	-0.311	-1.035	0.3711	271	1282.9	182.8	264.5	1.20	1282.8	181.4	265.8	1.16
Year	0.087	-0.059	0.2381	275	1306.1	186.5	268.9	1.25	1306.5	185.1	270.8	1.21
RFactor	-0.051	-0.167	0.0537	152	709.6	113.7	153.9	1.34	709.5	111.5	156	1.22
Das28	-0.486	-1.418	0.4411	162	525.3	102.8	105.5	0.13	524.8	102.1	105.7	0.14

© 2023 Guski LS et al. *JAMA Network Open.*

eTable 5: Primary Outcome and Sensitivity Outcomes with Placebo as Reference

Drug	TJCi (n=132)	Tjc (n=118)	SJC (n=97)	ESR (n=116)	CRP (n=47)	DAS28i (n=132)	Tjc28i (n=132)
Methotrexate	-5.18 (-6.27 to -4.09)	-5.11 (-6.22 to -3.99)	-4.44 (-5.36 to -3.53)	-13.34 (-16.73 to -9.87)	-13.26 (-21.84 to -4.98)	-0.88 (-1.51 to -0.25)	-4.14 (-4.97 to -3.28)
Sulfasalazine	-3.28 (-4.40 to -2.21)	-3.19 (-4.37 to -2.09)	-3.05 (-4.08 to -2.04)	-14.77 (-18.48 to -11.07)	-8.34 [a] (-18.72 to 1.27)	-0.74 (-1.40 to -0.065)	-2.44 (-3.25 to -1.67)
Leflunomide	-4.92 (-6.26 to -3.71)	-4.87 (-6.22 to -3.64)	-3.52 (-4.54 to -2.57)	-5.85 (-9.98 to -1.87)	-14.19 (-23.64 to -5.46)	-0.61 [a] (-1.32 to 0.074)	-3.80 (-4.78 to -2.89)
Cyclosporine	-4.39 (-5.88 to -2.94)	-4.33 (-5.82 to -2.85)	-3.29 (-4.45 to -2.16)	-3.36 [a] (-8.74 to 1.97)	-19.73 (-34.30 to -5.23)	-0.56 [a] (-1.44 to 0.31)	-3.27 (-4.30 to -2.26)
Gold, inj	-4.47 (-5.62 to -3.24)	-4.38 (-5.59 to -3.12)	-3.64 (-4.82 to -2.45)	-13.65 (-17.54 to -9.72)	-18.35 (-31.93 to -5.27)	-0.83 (-1.54 to -0.13)	-2.92 (-3.84 to -2.01)
Chloroquine	-2.67 (-3.94 to -1.44)	-2.70 (-4.11 to -1.34)	-2.24 (-3.36 to -1.12)	-6.13 (-9.58 to -2.77)	-2.12 [a] (-12.22 to 8.22)	-0.46 [a] (-1.19 to 0.27)	-1.98 (-2.89 to -1.13)
D-Penicillamine	-2.97 (-4.32 to -1.66)	-2.83 (-4.24 to -1.46)	-3.54 (-4.85 to -2.25)	-15.72 (-20.29 to -11.04)	-12.65 [a] (-27.82 to 2.51)	-0.77 [a] (-1.58 to 0.04)	-2.68 (-3.67 to -1.67)
Azathioprine	-2.61 (-4.29 to -0.98)	-2.50 (-4.20 to -0.82)	-3.04 (-4.52 to -1.55)	-6.30 [a] (-13.16 to 0.65)	-11.70 [a] (-27.89 to 4.50)	-0.54 [a] (-1.69 to 0.60)	-2.58 (-3.87 to -1.30)
Gold, oral	-2.47 (-3.87 to -1.12)	-2.08 (-3.56 to -0.65)	-1.99 (-3.23 to -0.77)	-6.66 (-10.39 to -2.93)	2.94 [a] (-10.86 to 16.46)	-0.43 [a] (-1.09 to 0.25)	-1.78 (-2.71 to -0.89)
Cyclophosphamide	-11.25 (-16.73 to -5.72)	-13.45 (-19.61 to -7.27)	-12.86 (-16.60 to -9.14)	-12.76 (-23.62 to -2.22)	NA	-0.98 [a] (-3.14 to 1.17)	-7.67 (-11.29 to -4.08)
Levamisole	-2.71 (-4.18 to -1.36)	-2.67 (-4.15 to -1.307)	-1.71 (-2.80 to -0.64)	-14.67 (-20.72 to -8.76)	-23.43 (-39.03 to -7.89)	-0.73 [a] (-1.77 to 0.35)	-3.08 (-4.26 to -1.89)
Tacrolimus	-6.49 (-9.07 to -3.94)	-6.50 (-9.09 to -3.93)	-3.61 (-5.37 to -1.86)	-15.06 (-22.27 to -7.86)	-13.73 (-24.15 to -4.29)	-0.77 [a] (-1.95 to 0.40)	-4.61 (-6.37 to -2.88)
Tripterygium wilfordii.	-3.87 [a] (-8.38 to 0.59)	-3.80 [a] (-8.27 to 0.67)	-4.13 (-6.71 to -1.52)	-11.52 (-21.26 to -1.84)	-12.89 [a] (-28.87 to 2.36)	-0.85 [a] (-2.83 to 1.14)	-2.94 [a] (-6.24 to 0.35)
Iguratimod	-4.05 (-6.07 to -2.01)	-3.99 (-6.02 to -1.94)	-3.69 (-5.18 to -2.21)	-12.68 (-20.45 to -5.06)	-13.3 (-24.43 to -2.33)	-0.75 [a] (-1.98 to 0.47)	-3.20 (-4.89 to -1.49)
Timegadine	-0.51 [a] (-14.51 to 13.56)	-0.36 [a] (-14.39 to 13.63)	-0.24 [a] (-12.37 to 11.58)	-33.35 (-57.63 to -9.46)	NA	-0.94 [a] (-5.69 to 3.96)	-1.19 [a] (-9.96 to 7.62)
Bucillamine	-4.32 [a] (-16.19 to 7.71)	-4.14 [a] (-16.23 to 7.98)	-5.73 [a] (-13.60 to 2.10)	-15.51 [a] (-45.61 to 15.56)	-23.56 [a] (-61.39 to 14.28)	-0.99 [a] (-5.63 to 3.61)	-3.64 [a] (-12.07 to 4.88)
Tiopronin	-2.179 [a] (-8.82 to 4.41)	-2.05 [a] (-8.69 to 4.57)	NA	-22.22 (-43.14 to -1.76)	NA	-0.89 [a] (-4.61 to 2.89)	-2.07 [a] (-6.98 to 2.78)
Pyritinol	-3.07 [a] (-7.18 to 1.00)	-2.68 [a] (-6.81 to 1.38)	NA	-14.53 (-25.06 to -3.96)	NA	-0.64 [a] (-2.48 to 1.22)	-2.23 [a] (-5.09 to 0.63)

© 2023 Guski LS et al. *JAMA Network Open.*

Mycophenolate	-1.61 [a] (-4.97 to 1.75)	-1.62 [a] (-4.98 to 1.76)	-1.31 [a] (-4.17 to 1.52)	-1.13 [a] (-8.19 to 5.89)	-0.060 [a] (-12.10 to 11.40)	-0.24 [a] (-1.80 to 1.31)	-1.62 [a] (-4.82 to 1.53)
Drug	TJCi (n=132)	Tjc (n=118)	SJC (n=97)	ESR (n=116)	CRP (n=47)	DAS28i (n=132)	Tjc28i (n=132)
OM 89801	-2.98 [a] (-6.47 to 0.49)	-2.66 [a] (-6.17 to 0.86)	-1.55 [a] (-3.18 to 0.013)	-7,00 [a] (-18.36 to 4.40)	NA	-0.55 [a] (-2.57 to 1.46)	-2.37 [a] (-4.85 to 0.13)
Lobenzarit	-1.63 (-5.02 to 1.74)	NA	-1.62 [a] (-4.59 to 1.32)	-7.89 [a] (-22.64 to 7.27)	NA	-0.33 [a] (-2.32 to 1.67)	-1.02 [a] (-3.08 to 1.05)
Rheumacon	-2.36 [a] (-4.92 to 0.14)	-2.15 [a] (-4.74 to 0.37)	-1.59 (-6.71 to 3.58)	8.71 [a] (-5.83 to 22.76)	3.1 [a] (-17.48 to 23.17)	-0.18 [a] (-2.21 to 1.89)	-1.78 (-3.52 to -0.028)
Prospidin	-5.39 [a] (-12.91 to 2.09)	-5.28 [a] (-12.86 to 2.25)	-7.78 [a] (-15.6 to 0.08)	-22.52 (-35.93 to -9.55)	-20.56 (-38.66 to -2.88)	-1.38 [a] (-5.08 to 2.32)	-4.19 [a] (-8.92 to 0.52)
Aminopterin	-15.56 [a] (-38.83 to 7.85)	-15.55 [a] (-39.22 to 8.00)	-12.77 [a] (-34.97 to 9.44)	NA	NA	-1.79 [a] (-9.33 to 5.62)	-12.52 [a] (-28.92 to 4.01)
Eazmowplus	-2.34 [a] (-14.87 to 9.95)	-2.35 [a] (-14.86 to 9.99)	-1.80 [a] (-9.39 to 5.82)	-2.36[a] (-14.49 to 9.92)	NA	-0.52 [a] (-4.84 to 3.86)	-1.89 [a] (-14.33 to 10.19)
Dapsone	-3.79 (-7.57 to -0.03)	-3.81 (-7.64 to -0.036)	-4.90 [a] (-10.56 to 0.81)	-9.87[a] (-25.03 to 5.70)	-17.65 [a] (-40.15 to 4.99)	-0.70 [a] (-3.32 to 1.94)	-2.76 (-5.38 to -0.20)
Glucocorticoid	-2.64 (-5.02 to -0.25)	-1.72 [a] (-4.71 to 1.26)	-1.20 [a] (-3.00 to 0.62)	-11.63 (-20 to -3.22)	-1.11 [a] (-19.48 to 17.24)	-0.58 [a] (-2.07 to 0.94)	-2.20 (-4.11 to -0.30)
.Methotrexate inj..	-6.20 (-9.59 to -2.79)	-6.14 (-9.52 to -2.69)	-3.64 (-1.34 to -5.94)	-8.58 [a] (-20.80 to 3.53)	-13.26 (-21.84 to -4.98)	-1.49 [a] (-3.79 to 0.86)	-4.72 (-6.84 to -2.56)
Placebo	ref	Ref	Ref	Ref	Ref	Ref	Ref

NA: Not applicable (no comparisons of this outcome within this intervention

[a] Not different from placebo

(-) indicates that the outcome associated with the intervention is greater than the outcome associated with placebo

eTable 6. Comparisons of Tender Joint Count Imputed (TJCi) Outcome of All Interventions vs All Interventions

g	Mt	Su	Le	Cs
Su	-1.902 (-3.152 ; -0.5672)			
Le	-0.262 (-1.231 ; 0.8935)	1.65 (0.2421 ; 3.073)		
Cs	-0.7853 (-2.413 ; 0.8824)	1.116 (-0.6342 ; 2.827)	-0.5353 (-2.38 ; 1.248)	
Au	-0.7105 (-2.1 ; 0.6134)	1.185 (-0.3692 ; 2.604)	-0.455 (-2.15 ; 1.03)	0.07328 (-1.578 ; 1.614)
Cl	-2.507 (-4.022 ; -0.9309)	-0.6106 (-2.115 ; 0.8959)	-2.258 (-3.968 ; -0.596)	-1.723 (-3.512 ; 0.08238)
Dp	-2.208 (-3.71 ; -0.6683)	-0.3086 (-1.85 ; 1.215)	-1.964 (-3.653 ; -0.301)	-1.424 (-3.098 ; 0.2827)
Az	-2.565 (-4.21 ; -0.8829)	-0.6711 (-2.542 ; 1.191)	-2.319 (-4.205 ; -0.478)	-1.784 (-3.573 ; 0.02538)
Af	-2.702 (-4.193 ; -1.179)	-0.8105 (-2.393 ; 0.7587)	-2.458 (-4.156 ; -0.791)	-1.921 (-3.789 ; -0.04197)
Cp	6.079 (0.4291 ; 11.67)	7.967 (2.327 ; 13.58)	6.31 (0.6182 ; 11.95)	6.852 (1.121 ; 12.53)
Lm	-2.46 (-4.112 ; -0.7107)	-0.5603 (-2.261 ; 1.159)	-2.209 (-4.033 ; -0.396)	-1.677 (-3.58 ; 0.2793)
Tc	1.312 (-1.464 ; 4.123)	3.21 (0.4226 ; 5.983)	1.56 (-1.31 ; 4.394)	2.097 (-0.8525 ; 5.056)
Tw	-1.294 (-5.687 ; 3.108)	0.598 (-3.897 ; 5.088)	-1.052 (-5.565 ; 3.437)	-0.5173 (-5.167 ; 4.131)
Ig	-1.131 (-2.99 ; 0.7073)	0.7711 (-1.478 ; 2.9)	-0.8754 (-3.055 ; 1.15)	-0.35 (-2.769 ; 2.037)
Tg	-4.661 (-18.77 ; 9.354)	-2.774 (-16.88 ; 11.24)	-4.424 (-18.57 ; 9.595)	-3.893 (-18.02 ; 10.14)
Bc	-0.8508 (-12.94 ; 11.03)	1.041 (-11.04 ; 12.89)	-0.621 (-12.71 ; 11.28)	-0.06885 (-12.13 ; 11.82)
Tp	-2.998 (-9.63 ; 3.699)	-1.111 (-7.751 ; 5.586)	-2.755 (-9.453 ; 3.97)	-2.211 (-8.881 ; 4.509)
Py	-2.108 (-6.217 ; 2.033)	-0.207 (-4.371 ; 3.936)	-1.856 (-6.077 ; 2.303)	-1.324 (-5.589 ; 2.955)
Mc	-3.567 (-7.09 ; -0.03043)	-1.667 (-5.216 ; 1.84)	-3.318 (-6.927 ; 0.2383)	-2.78 (-6.456 ; 0.8911)
Om	-2.201 (-5.737 ; 1.343)	-0.3042 (-3.903 ; 3.255)	-1.951 (-5.607 ; 1.652)	-1.414 (-5.104 ; 2.26)
Lz	-3.551 (-7.1 ; 0.007404)	-1.653 (-5.233 ; 1.862)	-3.3 (-6.953 ; 0.2795)	-2.773 (-6.458 ; 0.9094)
Rc	-2.82 (-5.239 ; -0.3234)	-0.9187 (-3.523 ; 1.681)	-2.567 (-5.187 ; 0.0209)	-2.04 (-4.822 ; 0.793)
Pd	0.2135 (-7.19 ; 7.651)	2.104 (-5.424 ; 9.648)	0.4617 (-7.041 ; 7.958)	0.9921 (-6.579 ; 8.603)
Ap	10.37 (-12.97 ; 33.61)	12.27 (-11.19 ; 35.56)	10.61 (-12.75 ; 33.86)	11.15 (-12.29 ; 34.48)
Em	-2.841 (-15.14 ; 9.718)	-0.9412 (-13.23 ; 11.59)	-2.594 (-14.93 ; 9.949)	-2.051 (-14.4 ; 10.52)
Ds	-1.383 (-5.239 ; 2.49)	0.5106 (-3.371 ; 4.367)	-1.137 (-5.09 ; 2.772)	-0.5988 (-4.591 ; 3.366)
Gc	-2.543 (-5.164 ; 0.07902)	-0.6442 (-3.286 ; 1.938)	-2.295 (-5.024 ; 0.368)	-1.76 (-4.557 ; 1.022)
Mx	1.035 (-2.244 ; 4.247)	2.93 (-0.6158 ; 6.355)	1.286 (-2.196 ; 4.619)	1.825 (-1.862 ; 5.388)
Pl	-5.179 (-6.272 ; -4.089)	-3.278 (-4.407 ; -2.218)	-4.923 (-6.264 ; -3.712)	-4.394 (-5.879 ; -2.943)
	Au	Cl	Dp	Az
Cl	-1.795 (-3.367 ; -0.1045)			
Dp	-1.496 (-2.835 ; -0.04423)	0.2995 (-1.325 ; 1.926)		
Az	-1.853 (-3.653 ; 0.04856)	-0.06138 (-2.022 ; 1.91)	-0.3591 (-2.192 ; 1.474)	
Af	-1.991 (-3.5 ; -0.3991)	-0.1997 (-1.941 ; 1.542)	-0.4971 (-2.159 ; 1.159)	-0.1412 (-2.143 ; 1.864)
Cp	6.796 (1.159 ; 12.41)	8.57 (2.883 ; 14.2)	8.272 (2.598 ; 13.91)	8.639 (2.841 ; 14.37)
Lm	-1.752 (-3.278 ; -0.04474)	0.04743 (-1.759 ; 1.879)	-0.2485 (-1.939 ; 1.448)	0.1103 (-1.952 ; 2.192)
Tc	2.028 (-0.7662 ; 4.9)	3.817 (0.9713 ; 6.68)	3.517 (0.638 ; 6.411)	3.879 (0.8238 ; 6.938)
Tw	-0.583 (-5.094 ; 3.991)	1.212 (-3.406 ; 5.808)	0.9108 (-3.693 ; 5.501)	1.273 (-3.404 ; 5.944)
Ig	-0.4253 (-2.603 ; 1.838)	1.374 (-0.9855 ; 3.662)	1.074 (-1.287 ; 3.364)	1.433 (-1.028 ; 3.831)
Tg	-3.945 (-18.05 ; 10.03)	-2.163 (-16.3 ; 11.87)	-2.455 (-16.5 ; 11.43)	-2.11 (-16.21 ; 11.93)
Bc	-0.1388 (-12.17 ; 11.73)	1.629 (-10.41 ; 13.54)	1.336 (-10.61 ; 13.13)	1.686 (-10.39 ; 13.64)
Tp	-2.281 (-8.876 ; 4.408)	-0.4828 (-7.146 ; 6.203)	-0.7982 (-7.251 ; 5.733)	-0.4267 (-7.136 ; 6.369)
Py	-1.394 (-5.483 ; 2.792)	0.4018 (-3.823 ; 4.599)	0.1066 (-4.086 ; 4.288)	0.4625 (-3.881 ; 4.794)
Mc	-2.854 (-6.39 ; 0.7453)	-1.059 (-4.665 ; 2.508)	-1.361 (-4.987 ; 2.248)	-0.9984 (-4.745 ; 2.769)
Om	-1.483 (-4.982 ; 2.083)	0.3066 (-3.325 ; 3.922)	0.01564 (-3.482 ; 3.48)	0.3758 (-3.392 ; 4.129)
Lz	-2.843 (-6.387 ; 0.7817)	-1.046 (-4.67 ; 2.539)	-1.343 (-4.997 ; 2.267)	-0.9871 (-4.764 ; 2.769)
Rc	-2.116 (-4.631 ; 0.5627)	-0.3058 (-3.034 ; 2.413)	-0.6088 (-3.307 ; 2.085)	-0.2508 (-3.147 ; 2.658)
Pd	0.9219 (-6.595 ; 8.513)	2.712 (-4.851 ; 10.31)	2.412 (-5.155 ; 10.01)	2.781 (-4.817 ; 10.39)
Ap	11.09 (-12.34 ; 34.41)	12.89 (-10.55 ; 36.16)	12.58 (-10.86 ; 35.9)	12.95 (-10.44 ; 36.25)
Em	-2.117 (-14.43 ; 10.45)	-0.3351 (-12.63 ; 12.18)	-0.6422 (-12.98 ; 11.93)	-0.281 (-12.64 ; 12.34)
Ds	-0.6681 (-4.556 ; 3.259)	1.121 (-2.437 ; 4.668)	0.8284 (-3.092 ; 4.706)	1.186 (-2.903 ; 5.223)
Gc	-1.831 (-4.452 ; 0.8593)	-0.0324 (-2.623 ; 2.514)	-0.3417 (-3.06 ; 2.36)	0.02235 (-2.885 ; 2.915)
Mx	1.746 (-1.702 ; 5.208)	3.541 (-0.09157 ; 7.076)	3.242 (-0.3734 ; 6.765)	3.598 (-0.08312 ; 7.186)
Pl	-4.465 (-5.62 ; -3.242)	-2.672 (-3.947 ; -1.443)	-2.971 (-4.316 ; -1.659)	-2.612 (-4.291 ; -0.9787)
	Af	Cp	Lm	Tc
Cp	8.779 (3.057 ; 14.41)			
Lm	0.2461 (-1.592 ; 2.108)	-8.525 (-14.19 ; -2.833)		
Tc	4.015 (1.112 ; 6.926)	-4.753 (-10.82 ; 1.348)	3.768 (0.8386 ; 6.676)	
Tw	1.405 (-3.179 ; 5.999)	-7.358 (-14.48 ; -0.2103)	1.158 (-3.517 ; 5.806)	-2.613 (-7.764 ; 2.526)
Ig	1.575 (-0.7733 ; 3.851)	-7.208 (-13.08 ; -1.316)	1.328 (-1.153 ; 3.698)	-2.443 (-5.765 ; 0.8122)
Tg	-1.967 (-16.09 ; 12.04)	-10.69 (-25.96 ; 4.276)	-2.216 (-16.34 ; 11.8)	-5.979 (-20.3 ; 8.266)
Bc	1.835 (-10.22 ; 13.73)	-6.937 (-20.12 ; 6.207)	1.58 (-10.49 ; 13.49)	-2.191 (-14.47 ; 9.965)
Tp	-0.2953 (-6.995 ; 6.439)	-9.067 (-17.6 ; -0.4095)	-0.5406 (-7.241 ; 6.165)	-4.318 (-11.4 ; 2.775)
Py	0.602 (-3.251 ; 4.459)	-8.173 (-15.02 ; -1.279)	0.3575 (-3.925 ; 4.584)	-3.412 (-8.242 ; 1.374)
Mc	-0.8587 (-4.503 ; 2.755)	-9.633 (-16.08 ; -3.146)	-1.111 (-4.79 ; 2.518)	-4.874 (-9.131 ; -0.6651)
Om	0.5105 (-2.857 ; 3.845)	-8.262 (-14.8 ; -1.724)	0.2606 (-3.445 ; 3.924)	-3.51 (-7.859 ; 0.8026)
Lz	-0.8485 (-4.519 ; 2.781)	-9.615 (-16.04 ; -3.133)	-1.092 (-4.795 ; 2.536)	-4.861 (-9.128 ; -0.6408)

© 2023 Guski LS et al. *JAMA Network Open.*

Rc	-0.1131 (-2.607 ; 2.386)	-8.885 (-14.91 ; -2.786)	-0.3578 (-3.187 ; 2.427)	-4.135 (-7.717 ; -0.5234)
Pd	2.907 (-4.641 ; 10.49)	-5.833 (-15.13 ; 3.449)	2.659 (-4.947 ; 10.28)	-1.106 (-9.04 ; 6.847)
Ap	13.08 (-10.34 ; 36.36)	4.372 (-19.74 ; 28.22)	12.83 (-10.58 ; 36.12)	9.064 (-14.39 ; 32.5)
Em	-0.1432 (-12.47 ; 12.43)	-8.889 (-22.4 ; 4.764)	-0.3898 (-12.77 ; 12.21)	-4.146 (-16.68 ; 8.634)
Ds	1.322 (-2.649 ; 5.251)	-7.447 (-14.11 ; -0.7539)	1.075 (-2.955 ; 5.033)	-2.704 (-7.269 ; 1.841)
Gc	0.1612 (-2.584 ; 2.889)	-8.615 (-14.59 ; -2.566)	-0.08458 (-2.871 ; 2.64)	-3.862 (-7.388 ; -0.3557)
Mx	3.742 (0.1337 ; 7.243)	-5.039 (-11.51 ; 1.411)	3.489 (-0.2142 ; 7.088)	-0.2834 (-4.592 ; 3.957)
Pl	-2.473 (-3.863 ; -1.117)	-11.25 (-16.73 ; -5.716)	-2.713 (-4.18 ; -1.357)	-6.491 (-9.072 ; -3.937)
	Tw	Ig	Tg	Bc
Ig	0.1648 (-4.632 ; 4.905)			
Tg	-3.351 (-18.11 ; 11.25)	-3.518 (-17.75 ; 10.6)		
Bc	0.4649 (-12.42 ; 13.07)	0.2651 (-11.92 ; 12.3)	3.756 (-14.53 ; 22.14)	
Tp	-1.696 (-9.657 ; 6.303)	-1.861 (-8.696 ; 5.035)	1.703 (-13.62 ; 17.07)	-2.147 (-15.54 ; 11.46)
Py	-0.8042 (-6.803 ; 5.18)	-0.9719 (-5.441 ; 3.534)	2.565 (-11.94 ; 17.18)	-1.227 (-13.74 ; 11.43)
Mc	-2.271 (-7.866 ; 3.348)	-2.432 (-6.332 ; 1.5)	1.097 (-13.29 ; 15.59)	-2.702 (-15.09 ; 9.741)
Om	-0.8987 (-6.486 ; 4.706)	-1.064 (-5.017 ; 2.906)	2.469 (-11.86 ; 16.9)	-1.333 (-13.66 ; 11.14)
Lz	-2.262 (-7.88 ; 3.347)	-2.421 (-6.359 ; 1.539)	1.131 (-13.23 ; 15.58)	-2.675 (-15.01 ; 9.809)
Rc	-1.517 (-6.488 ; 3.435)	-1.688 (-4.717 ; 1.425)	1.856 (-12.33 ; 16.14)	-1.936 (-14.07 ; 10.32)
Pd	1.525 (-7.114 ; 10.14)	1.351 (-6.284 ; 9.014)	4.912 (-10.92 ; 20.77)	1.101 (-12.95 ; 15.3)
Ap	11.68 (-12.07 ; 35.42)	11.52 (-11.88 ; 34.81)	15.05 (-12.08 ; 42.2)	11.27 (-14.86 ; 37.55)
Em	-1.523 (-14.61 ; 11.68)	-1.7 (-14.12 ; 10.98)	1.836 (-16.64 ; 20.67)	-1.965 (-18.93 ; 15.42)
Ds	-0.09677 (-5.903 ; 5.709)	-0.2472 (-4.481 ; 3.996)	3.293 (-11.17 ; 17.89)	-0.5 (-12.93 ; 12.03)
Gc	-1.244 (-6.319 ; 3.808)	-1.411 (-4.518 ; 1.729)	2.093 (-12.03 ; 16.43)	-1.686 (-13.78 ; 10.54)
Mx	2.328 (-3.152 ; 7.772)	2.16 (-1.589 ; 5.885)	5.721 (-8.674 ; 20.13)	1.88 (-10.47 ; 14.38)
Pl	-3.874 (-8.377 ; 0.5854)	-4.045 (-6.07 ; -2.005)	-0.5146 (-14.51 ; 13.56)	-4.324 (-16.19 ; 7.707)
	Tp	Py	Mc	Om
Py	0.8868 (-6.877 ; 8.592)			
Mc	-0.5714 (-8.003 ; 6.809)	-1.46 (-6.766 ; 3.826)		
Om	0.8059 (-6.579 ; 8.12)	-0.09363 (-5.222 ; 4.968)	1.367 (-3.457 ; 6.203)	
Lz	-0.557 (-8.019 ; 6.839)	-1.445 (-6.779 ; 3.815)	0.002666 (-4.729 ; 4.777)	-1.352 (-6.218 ; 3.479)
Rc	0.1936 (-6.864 ; 7.188)	-0.7066 (-5.291 ; 3.878)	0.7525 (-3.423 ; 4.979)	-0.6227 (-4.72 ; 3.502)
Pd	3.222 (-6.767 ; 13.19)	2.319 (-6.156 ; 10.81)	3.789 (-4.375 ; 11.99)	2.402 (-5.816 ; 10.66)
Ap	13.39 (-10.91 ; 37.61)	12.51 (-11.18 ; 36.15)	13.93 (-9.655 ; 37.34)	12.56 (-10.99 ; 35.97)
Em	0.1739 (-13.96 ; 14.35)	-0.7298 (-13.64 ; 12.42)	0.7225 (-12.01 ; 13.63)	-0.6479 (-13.39 ; 12.28)
Ds	1.612 (-5.941 ; 9.189)	0.7263 (-4.785 ; 6.222)	2.181 (-2.864 ; 7.232)	0.8089 (-4.27 ; 5.891)
Gc	0.456 (-6.586 ; 7.457)	-0.4371 (-5.19 ; 4.267)	1.015 (-3.108 ; 5.165)	-0.3492 (-4.57 ; 3.866)
Mx	4.02 (-3.386 ; 11.38)	3.131 (-2.133 ; 8.315)	4.595 (-0.2116 ; 9.368)	3.229 (-1.574 ; 7.988)
Pl	-2.179 (-8.82 ; 4.41)	-3.073 (-7.18 ; 0.9956)	-1.611 (-4.974 ; 1.746)	-2.981 (-6.465 ; 0.4935)
	Lz	Rc	Pd	Ap
Rc	0.7359 (-3.447 ; 4.967)			
Pd	3.778 (-4.454 ; 12.02)	3.032 (-4.761 ; 10.82)		
Ap	13.92 (-9.686 ; 37.45)	13.2 (-10.43 ; 36.64)	10.13 (-14.28 ; 34.59)	
Em	0.7084 (-12.03 ; 13.61)	-0.01049 (-12.53 ; 12.72)	-3.016 (-17.45 ; 11.38)	-13.18 (-39.5 ; 13.49)
Ds	2.161 (-2.853 ; 7.252)	1.432 (-3.065 ; 5.887)	-1.6 (-9.985 ; 6.773)	-11.75 (-35.3 ; 11.88)
Gc	1.014 (-3.138 ; 5.126)	0.2782 (-3.219 ; 3.702)	-2.755 (-10.64 ; 5.107)	-12.94 (-36.32 ; 10.55)
Mx	4.589 (-0.2565 ; 9.337)	3.853 (-0.2791 ; 7.847)	0.8075 (-7.328 ; 8.924)	-9.368 (-32.76 ; 14.17)
Pl	-1.625 (-5.024 ; 1.745)	-2.357 (-4.919 ; 0.1352)	-5.389 (-12.91 ; 2.089)	-15.56 (-38.83 ; 7.846)
	Em	Ds	Gc	Mx
Ds	1.461 (-11.54 ; 14.25)			
Gc	0.2948 (-12.42 ; 12.83)	-1.156 (-5.542 ; 3.2)		
Mx	3.858 (-9.097 ; 16.59)	2.418 (-2.638 ; 7.396)	3.577 (-0.6157 ; 7.706)	
Pl	-2.344 (-14.87 ; 9.952)	-3.794 (-7.565 ; -0.02971)	-2.637 (-5.015 ; -0.250)	-6.206 (-9.586 ; -2.787)

Comparison between individual treatment principles in the NMA with DAS28 regression. Median, 2,5% fractile, 97,5 % fractile. Rows used as reference., so a negative value with a 95% CrI not including zero means the treatment principle in the row is significantly superior to the one in the column, while a positive value means the treatment principle in the row is significantly inferior to the one in the column.

© 2023 Guski LS et al. *JAMA Network Open.*

© 2023 Guski LS et al. *JAMA Network Open.*

eTable 7. Model Fit Data for Primary Outcome and Sensitivity Outcomes

Model	Total number of arms	Total residual deviance	DIC	Between-study S.D. (median, 95% CrI)
TJCi NMA	275	276	1304.03	0.8703 (0.05991; 1.574)
TJCi UME	275	274.5	1327.04	0.7949 (0.06692; 1.622)
Tjc NMA	247	251.2	1167.5	0.8706 (0.08874; 1.588)
Sjc NMA	202	187.5	848.091	0.328 (0.01545; 0.9122)
ESR NMA	249	226.4	1714.28	1.762 (0.1094; 3.857)
CRP NMA	97	94.65	697.201	4.006 (1.019; 4.955)
DAS28i NMA	275	284.6	90.284	0.2465 (0.1805; 0.3231)
TJC28i NMA	275	263.6	969.572	0.4476 (0.02678;0.98)

NMA: network meta-analysis

UME, unrelated mean effect (only performed for primary outcome, TJCi)

eTable 8. Primary and Sensitivity Outcomes Calculated With Prior 0-20

Drug	TJCi (n=132)	TJC (n=118)	SJC (n=97)	ESR (n=116)	CRP (n=47)	DAS28 (n=132)	TJC28 (n=132)
Methotrexate (Mt)(1)	Ref	Ref	Ref	Ref	Ref	Ref	Ref
Sulfasalazine (Su)(2)	-1.888 (-3.138;-0.5548)	-1.923 (-3.209;-0.5572)	-1.384 (-2.502;-0.2501)	1.426 (2.559;5.523)	-4.906 (-14.24;4.966)	-0.05761 (0.2639;0.1509)	-1.68 (-2.631;-0.6936)
Leflunomide (Le)(3)	-0.2573 (-1.234;0.9045)	-0.2523 (-1.228;0.9029)	-0.9226 (-1.564;-0.1936)	-7.522 (-10.48;-4.08)	0.8983 (-5.104;7.571)	-0.1235 (0.3339;0.0995)	-0.3473 (-1.038;0.5042)
Cyclosporine (Cs)(4)	-0.7875 (-2.433;0.8821)	-0.7763 (-2.405;0.9239)	-1.138 (-2.38;0.1061)	-9.953 (-15.61;-4.3)	6.517 (-7.976;20.64)	-0.2593 (-0.5124;-0.005)	-0.8583 (-2.038;0.3507)
Inj.gold (Au)(5)	-0.7241 (-2.106;0.6099)	-0.7191 (-2.112;0.6353)	-0.7961 (-2.061;0.4237)	0.3007 (-3.99;4.678)	5.088 (-6.251;16.72)	-0.03821 (-0.2358;0.159)	-1.216 (-2.249;-0.1578)
Chloroquine(Cl)(6)	-2.51 (-4.018;-0.9334)	-2.407 (-4.032;-0.7317)	-2.189 (-3.593;-0.7808)	-7.191 (-11.76;-2.51)	-11.19 (-23.13;0.3581)	-0.3464 (-0.5796;-0.113)	-2.14 (-3.283;-0.9719)
Penicillamine (Dp)(7)	-2.216 (-3.709;-0.6503)	-2.272 (-3.788;-0.6596)	-0.8718 (-2.336;0.524)	2.366 (-2.82;7.534)	-0.6517 (-16.34;14.81)	-0.06524 (-0.2808;0.15)	-1.459 (-2.633;-0.2657)
Azathioprine (Az)(8)	-2.569 (-4.22;-0.8924)	-2.589 (-4.26;-0.8461)	-1.392 (-2.89;0.09376)	-7.035 (-13.97;0.1)	-1.573 (-16.67;13.43)	-0.3361 (-0.5859;-0.087)	-1.553 (-2.914;-0.1647)
Oral gold (Af)(9)	-2.711 (-4.175;-1.189)	-3.023 (-4.51;-1.45)	-2.445 (-3.749;-1.107)	-6.682 (-10.81;-2.51)	-16.22 (-30.16;-2.422)	-0.3922 (-0.5958;-0.189)	-2.35 (-3.363;-1.296)
Cyclophosphamide (Cp)(10)	6.074 (0.4392;11.66)	8.287 (1.973;14.75)	8.433 (4.562;12.39)	-0.5645 (-11.81;10.9)	NA	0.4008 (-0.17;0.9735)	3.543 (-0.1046;7.281)
Levamisole (Lm)(11)	-2.467 (-4.116;-0.6994)	-2.439 (-4.078;-0.6748)	-2.718 (-4.069;-1.356)	1.324 (-5.208;8.16)	10.18 (-6.758;26.83)	-0.05492 (-0.363;0.2566)	-1.049 (-2.477;0.4136)
Tacrolimus (Tl)(12)	1.317 (-1.447;4.148)	1.386 (-1.413;4.234)	-0.8104 (-2.835;1.173)	1.721 (-6.31;9.797)	0.4825 (-12.18;13.71)	-0.01273 (-0.3958;0.37)	0.4741 (-1.428;2.422)
TripterygiumWilf. (Tw)(13)	-1.296 (-5.639;3.096)	-1.315 (-5.61;3.072)	-0.3109 (-2.767;2.138)	-1.821 (-11.05;7.66)	-0.4233 (-14.49;14.19)	0.01686 (-0.457;0.4906)	-1.176 (-4.371;2.048)
Iguratimod (Ig)(14)	-1.134 (-2.998;0.7118)	-1.109 (-2.963;0.7542)	-0.7483 (-2.042;0.5572)	-0.6491 (-7.95;6.758)	-0.005738 (-9.579;9.491)	-0.06914 (-0.4273;0.288)	-0.9421 (-2.475;0.6031)
Timegadine (Tg)(15)	-4.521 (-18.81;9.268)	-4.749 (-19;9.377)	-4.354 (-15.98;8.107)	19.97 (-3.85;44.44)	NA	0.06392 (-0.806;0.9405)	-3.21 (-11.9;5.707)
Bucillamin (Bc)(16)	-1.045 (-12.88;11.03)	-0.9875 (-13.46;11.02)	1.375 (-6.342;9.102)	2.15 (-28.9;32.35)	10.34 (-27.66;48.19)	0.08161 (-0.7557;0.926)	-0.4403 (-8.981;8.148)
Tiopronin (Tp)(17)	-3.022 (-9.645;3.648)	-3.064 (-9.716;3.641)	NA	8.9 (-11.57;30)	NA	0.03639 (-0.7705;0.844)	-2 (-6.978;2.95)
Pyritinol (Py)(18)	-2.108 (-6.195;2.035)	-2.422 (-6.523;1.8)	NA	1.196 (-9.586;11.9)	NA	-0.1605 (-0.7579;0.432)	-1.927 (-4.804;1.036)
Mycophenolate (Mc)(19)	-3.567 (-7.111;0.038)	-3.462 (-7.023;0.0235)	-3.115 (-6.191;-0.112)	-12.19(-19.99;-4.377)	-13.23 (-27.55;1.338)	-0.6221 (-1.068;-0.1733)	-2.513 (-5.784;0.7693)
OM89801 (Om)(20)	-2.208 (-5.735;1.353)	-2.45 (-6.019;1.116)	-2.875 (-4.593;-1.167)	-6.325 (-17.8;5.157)	NA	-0.339 (-0.883;0.1999)	-1.792 (-4.275;0.7638)
Lobenzarit (Lz)(21)	-3.55 (-7.115;0.0215)		-2.78 (-5.904;0.3037)	-5.447 (-21.02;9.73)	NA	-0.5226(-1.138;0.0948)	-3.107 (-5.315;-0.8661)
Rheumacon (Rc)(22)	-2.825 (-5.252;-0.3076)	-2.968 (-5.364;-0.4694)	-2.928 (-7.922;2.094)	-22.01 (-35.77;-7.86)	-16.39 (-35.7;3.285)	-0.5661 (-1.075;-0.0548)	-2.358 (-4.044;-0.64)
Prospidin (Pd)(23)	0.2422 (-7.191;7.688)	0.1066 (-7.372;7.64)	3.361 (-4.369;11.1)	9.2 (-3.32;22.07)	7.277 (-8.407;23.23)	0.5246 (-0.3609;1.4)	0.07493 (-4.585;4.731)
Aminopterin (Ap)(24)	10.37 (-12.68;33.51)	10.26 (-13.44;33.89)	8.488 (-13.31;30.18)	NA	NA	0.6024 (-0.5662;1.761)	8.231 (-8.903;24.19)
Drug	TJCi (n=132)	TJC (n=118)	SJC (n=97)	ESR (n=116)	CRP (n=47)	DAS28 (n=132)	TJC28 (n=132)

© 2023 Guski LS et al. *JAMA Network Open.*

Eazmowplus (Em)(25)	-2.925 (-15.29;9.36)	-2.829 (-15.18;9.965)	-2.661 (-10.35;5.242)	-11 (-23.68;1.56)		-0.4704 (-1.253;0.3218)	-2.23 (-14.73;10.74)
Dapsone (Ds)(26)	-1.386 (-5.24;2.488)	-1.311 (-5.208;2.692)	0.4688 (-5.358;6.295)	-3.458 (-19.3;12.04)	4.266 (-18.95;27.47)	-0.08403 (-0.7036;0.53)	-1.375 (-4.036;1.312)
Glucocorticoide (Gc)(27)	-2.548 (-5.139;0.059)	-3.376 (-6.486;-0.2082)	-3.233 (-5.262;-1.211)	-1.715 (-10.7;7.324)	-12.16 (-32.55;7.816)	-0.2754 (-0.6668;0.114)	-1.911 (-3.987;0.1798)
Inj.methotrexate (Mx)(28)	1.01 (-2.285;4.218)	1.048 (-2.228;4.249)	-0.7943 (-2.973;1.356)	-4.782 (-16.98;7.59)		0.5223 (0.05041;0.988)	0.5853 (-1.408;2.554)
Placebo (Pl)(29)	-5.178 (-6.276;-4.078)	-5.102 (-6.202;-3.981)	-4.431 (-5.354;-3.515)	-13.34 (-16.73;-9.87)	-13.3 (-21.81;-4.967)	-0.8318 (-1.001;-0.6634)	-4.127 (-4.96;-3.282)

eFigure 1. Subgroup Analyses

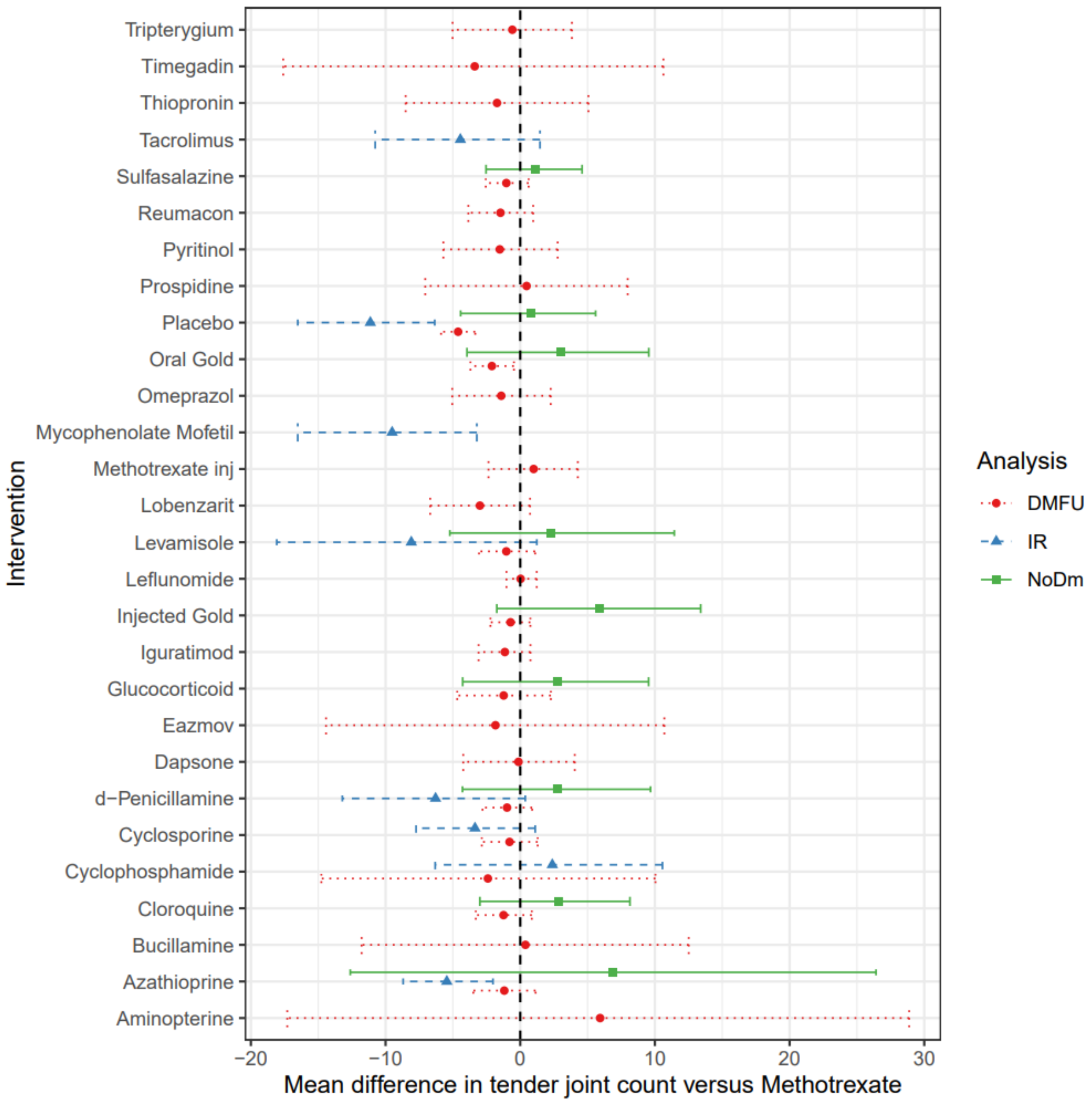

eFigure 1A IR: Previous inadequate response with a csDMARD; DMFU: Previous use of csDMARDs allowed, but fraction of patients unspecified. NoDm: No previous csDMARD use

© 2023 Guski LS et al. *JAMA Network Open.*

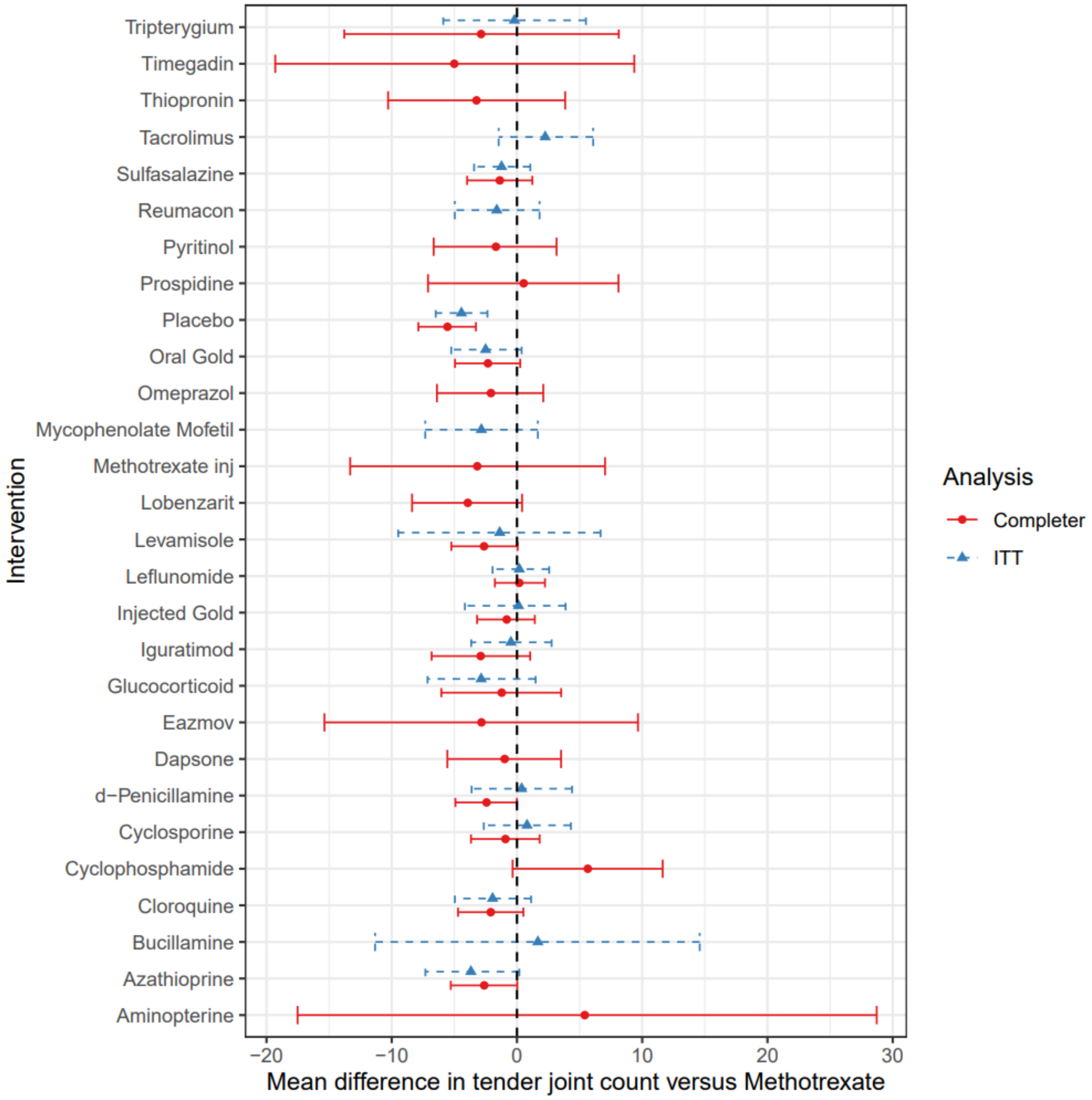

eFigure 1B: ITT: Intention to treat and completer trials

© 2023 Guski LS et al. *JAMA Network Open.*

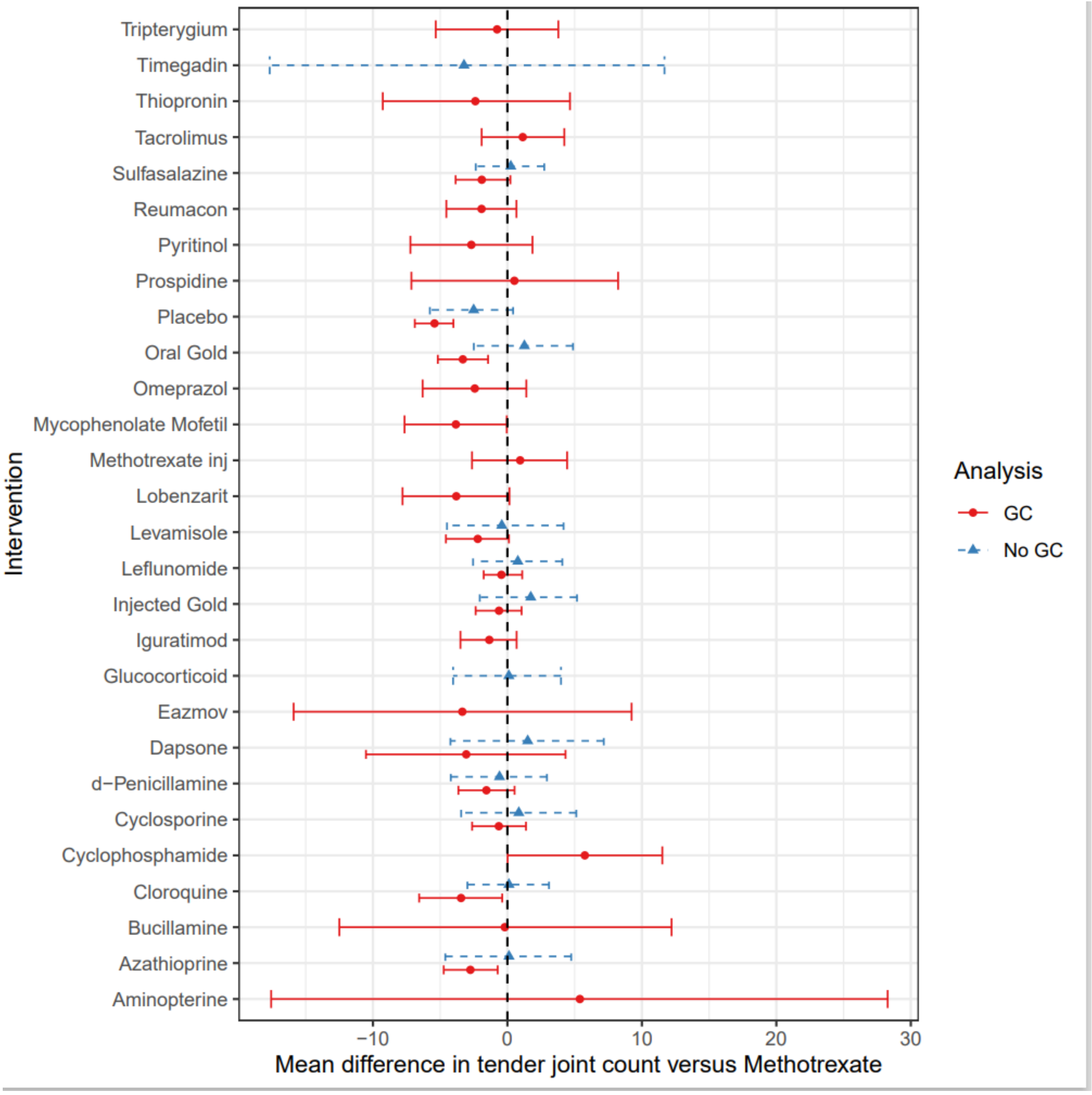

eFigure 1C: GC: glucocorticoid allowed; No GC: Glucocorticoid not allowed

© 2023 Guski LS et al. *JAMA Network Open.*

eFigure 2. Forest Plot of Primary Tender Joint Count Imputed (TJCi) Outcomes vs Peroral Methotrexate

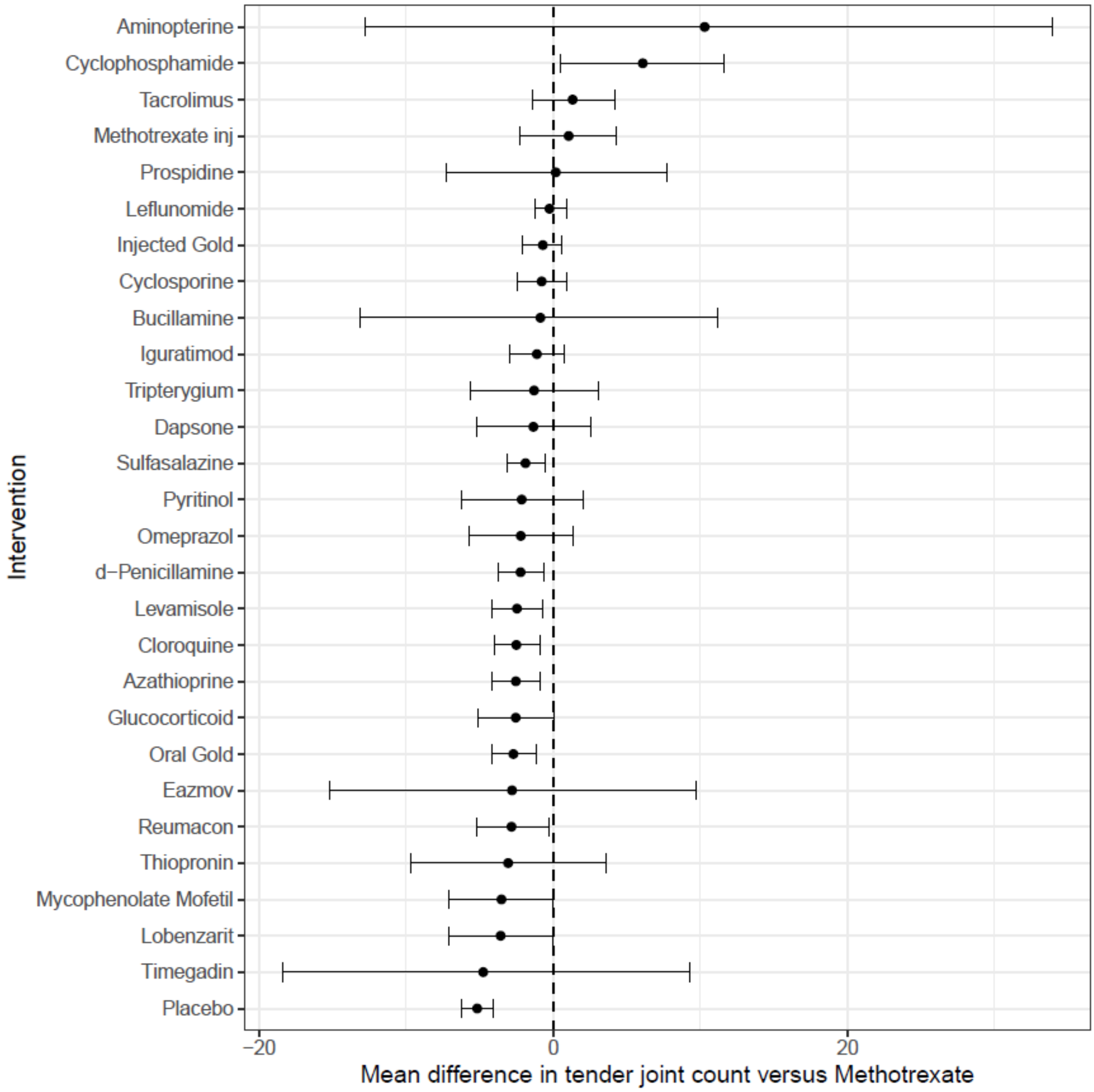

eFigure 2: Caterpillar plot: Mean treatment effect and 95% CI for each of the 29 treatments.

© 2023 Guski LS et al. *JAMA Network Open.*

eFigure 3. Rankograms of 29 Tested Interventions

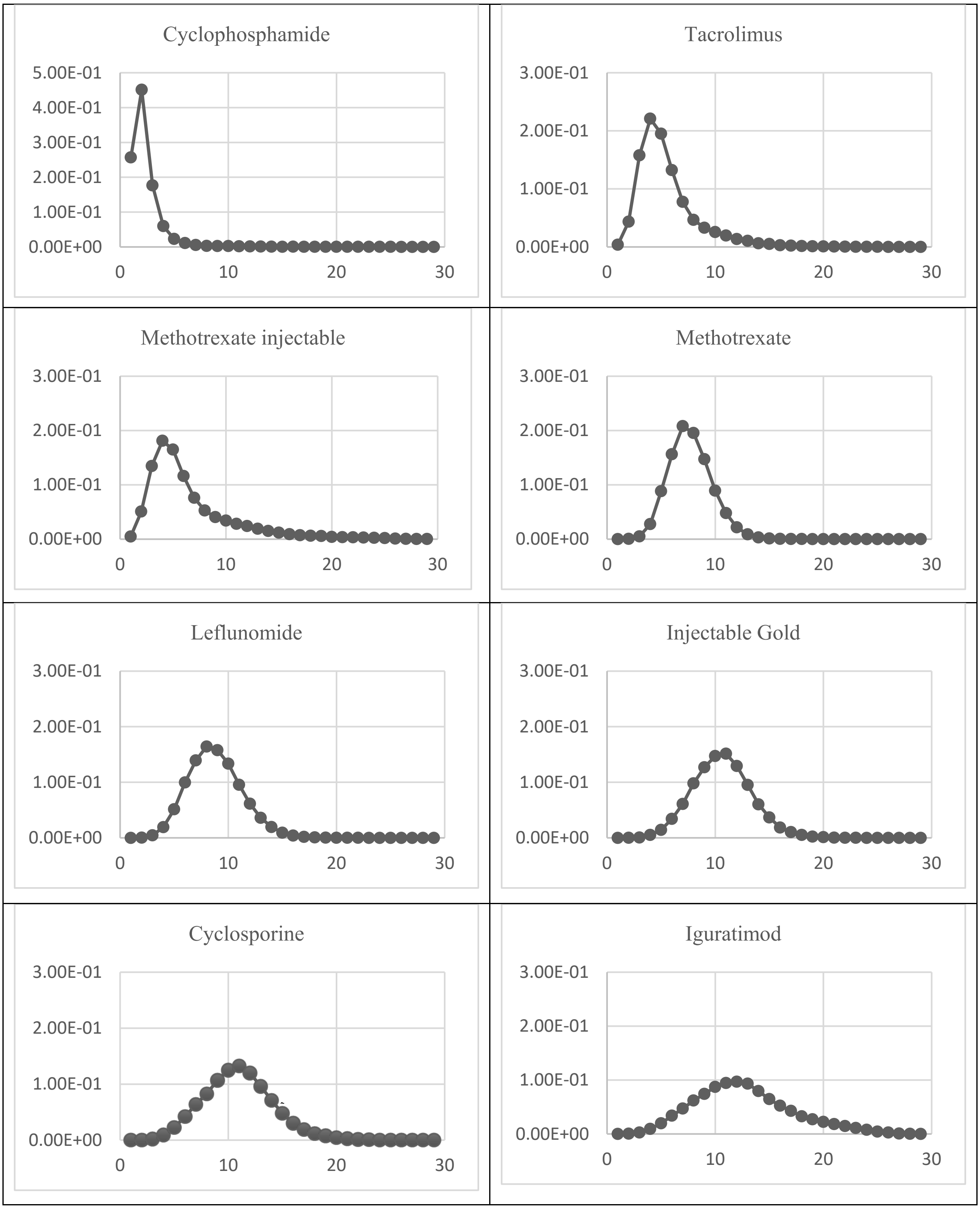

© 2023 Guski LS et al. *JAMA Network Open.*

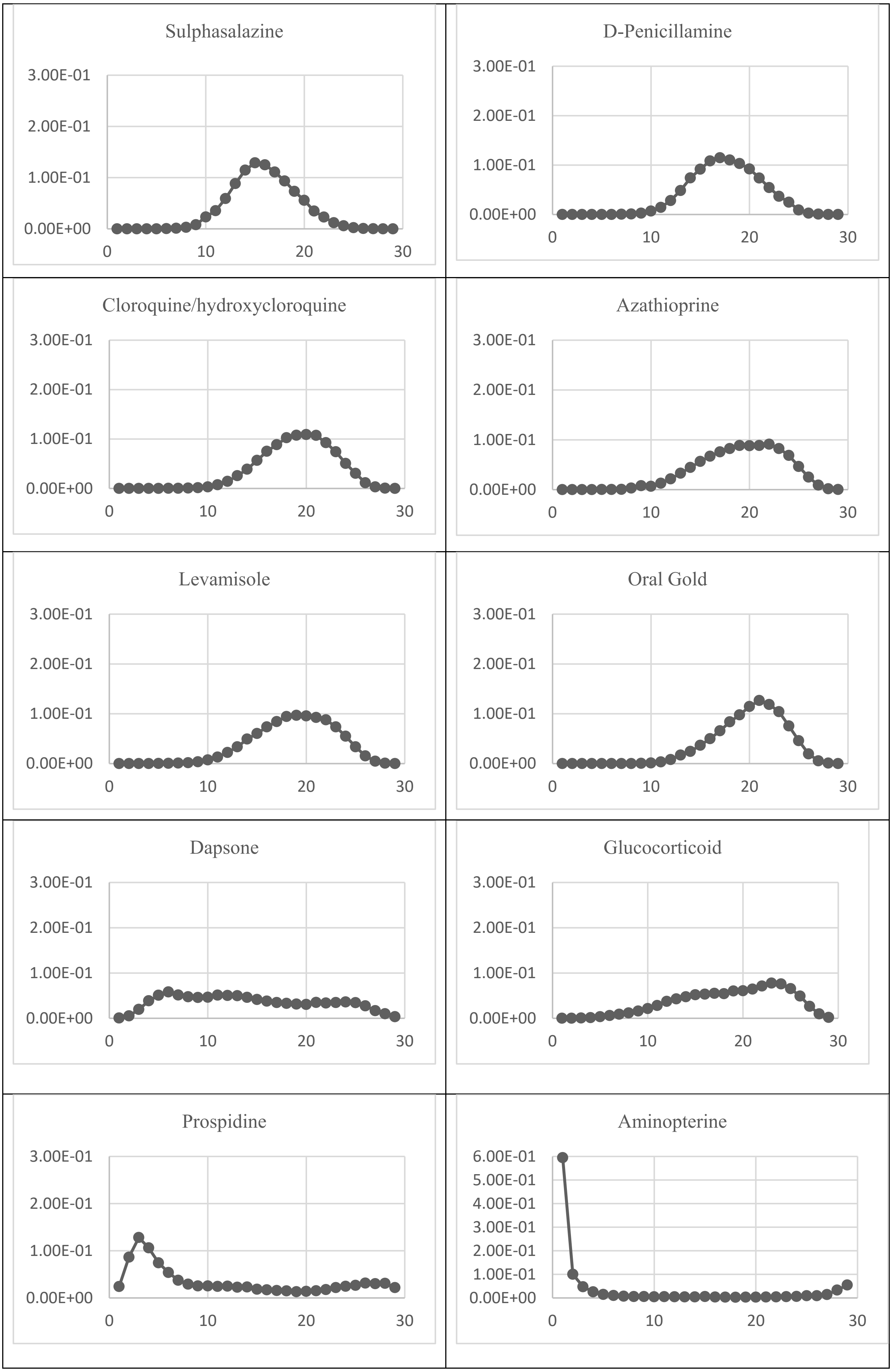

© 2023 Guski LS et al. *JAMA Network Open.*

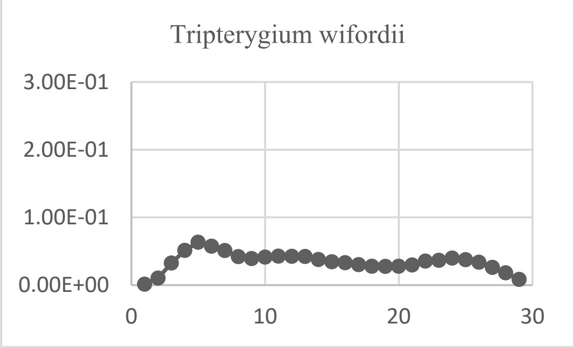

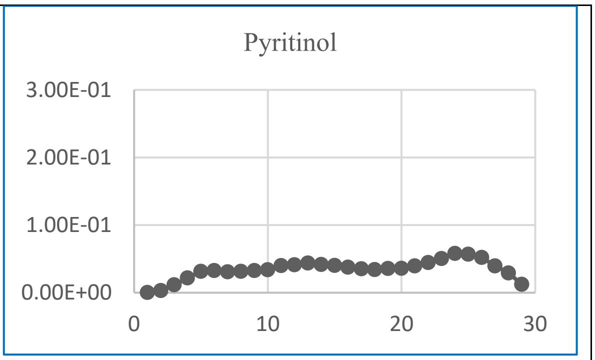

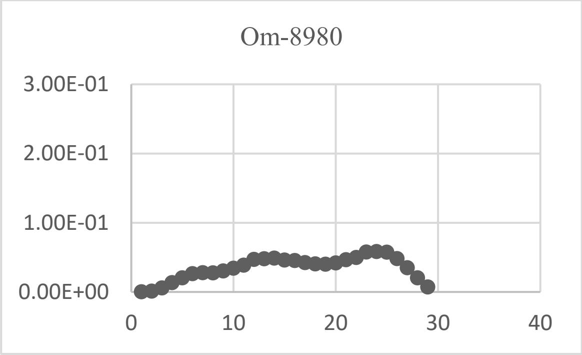

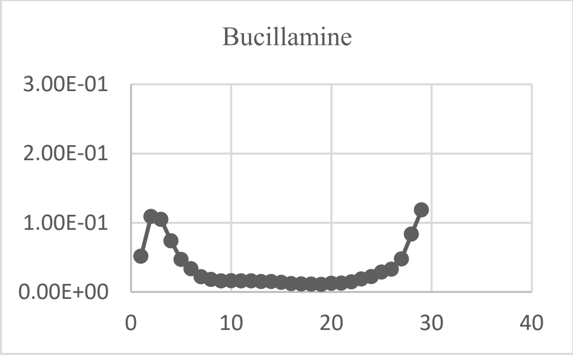

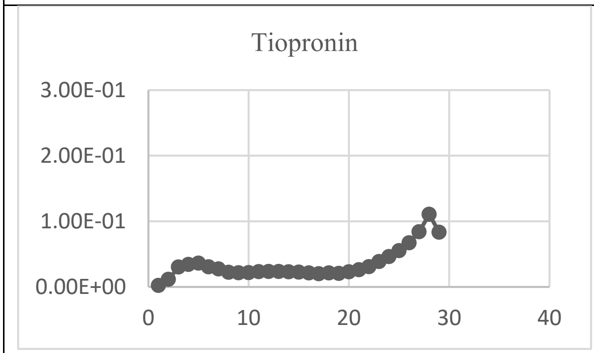

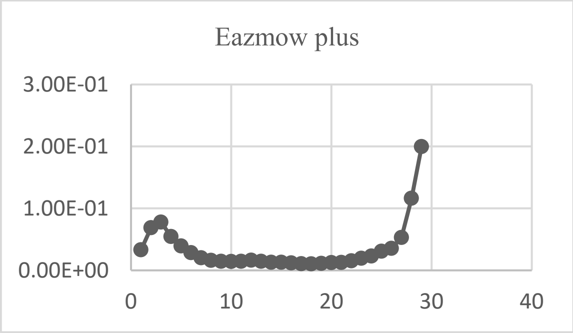

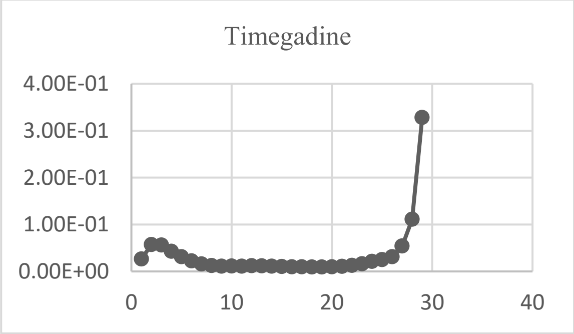

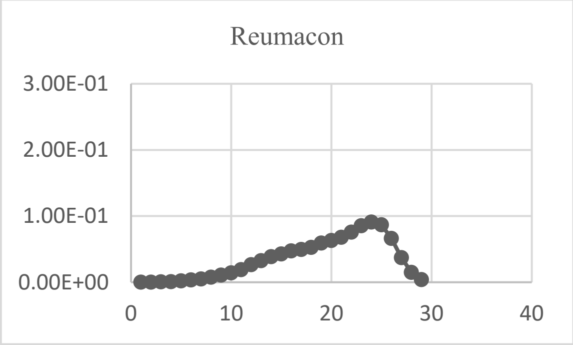

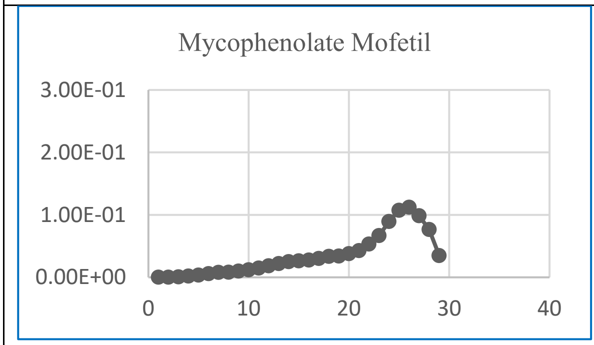

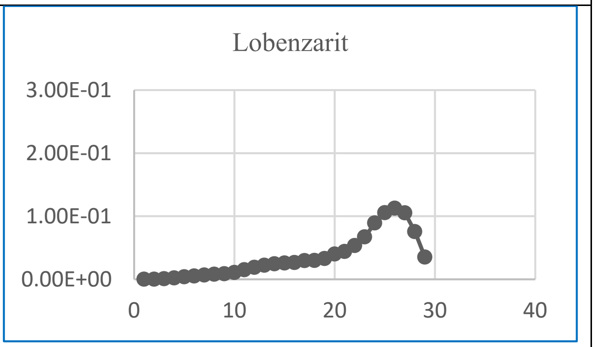

© 2023 Guski LS et al. *JAMA Network Open.*

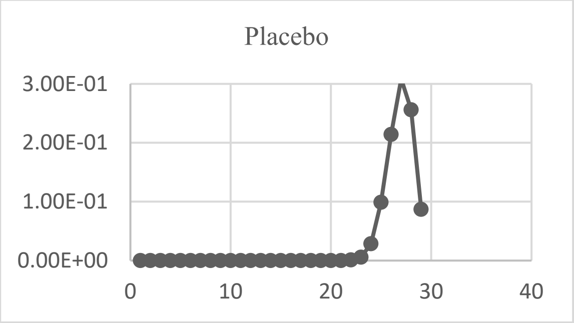

eFigure 4. dev-dev Plot of Unrelated Mean Effects Mean vs Network Meta-Analyses Mean

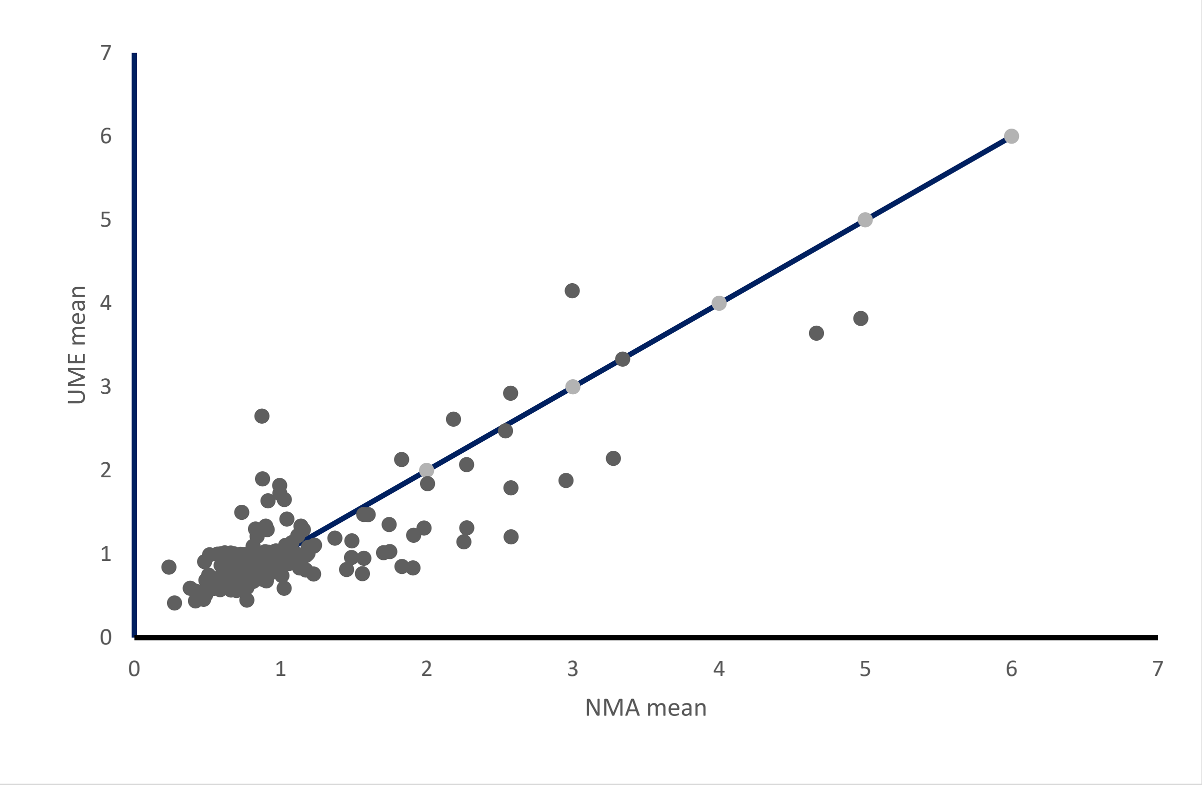

Plot showing mean in unrelated mean effects model versus mean in model with mixed treatment effects.

© 2023 Guski LS et al. *JAMA Network Open.*

© 2023 Guski LS et al. *JAMA Network Open.*
