# Supplementary material for: Monotreatment With Conventional Antirheumatic Drugs or Glucocorticoids in Rheumatoid Arthritis: A Network Meta-Analysis
Source: JAMA Netw Open. 2023 Oct 6;6(10):e2335950. doi: 10.1001/jamanetworkopen.2023.35950 (PMC10559183; doi:10.1001/jamanetworkopen.2023.35950)
Supplement: Supplement 2. — Data Sharing Statement [file jamanetwopen-e2335950-s002.pdf]

Data Sharing Statement

Guski. Mono Treatment With Conventional Antirheumatic Drugs or Glucocorticoids in Rheumatoid Arthritis: A Network Meta-Analysis. *JAMA Netw Open*. Published online September 27, 2023.
doi:10.1001/jamanetworkopen.2023.35950

Data
Data available: Yes
Data types: Data (not involving human participants)
How to access data: Data are shown in supplement etable1
When available: With publication

Supporting Documents
Document types: Other (please specify)
Additional Information: Supplement with detailed methods and supplementary results
How to access documents: Online supplement
When available: With publication

Additional Information
Who can access the data: The data are intended to be avilable in an online supplement, if possible.
Types of analyses: The data are available for control of our analyses, not for independent publications.
Mechanisms of data availability: For the purpose of control the data are available without investigator support.
Any additional restrictions: As mentoned, the availabale data cannot be used for independent publications
